# Early Nutritional Interventions for Brain and Cognitive Development in Preterm Infants: A Review of the Literature

**DOI:** 10.3390/nu9030187

**Published:** 2017-02-23

**Authors:** Nora Schneider, Clara L. Garcia-Rodenas

**Affiliations:** Nestec Ltd., Nestlé Research Center, Vers-Chez-les Blanc, 1000 Lausanne 26, Switzerland; clara.garcia@rdls.nestle.com

**Keywords:** diet, nutrition, brain, cognition, preterm infants

## Abstract

Adequate nutrition is important for neurodevelopmental outcomes in preterm-born infants. In this review, we aim to summarize the current knowledge on nutritional interventions initiated during the hospital stay targeting brain and cognitive development benefits in preterm human infants. Studies can broadly be split in general dietary intervention studies and studies investigating specific nutrients or nutritional supplements. In general, mother’s breast milk was reported to be better for preterm infants’ neurodevelopment compared to infant formula. The differences in methodologies make it difficult to conclude any effects of interventions with individual nutrients. Only protein and iron level studies showed some consistent findings regarding optimal doses; however, confirmatory studies are needed. This review does not support some widely accepted associations, such as that between long-chain polyunsaturated fatty acid supplementation and visual development. Clear nutritional recommendations cannot be made based on this review. However, the type of infant nutrition (i.e., breast milk versus formula or donor milk), the timing of the nutritional intervention, and the dose of the nutrient/supplement have been found to be relevant factors in determining the success of nutritional intervention studies in preterm infants.

## 1. Introduction

The WHO has defined ”preterm” as an infant born before 37 weeks of pregnancy are completed, and has categorized the preterm population into “extremely preterm” (EPT; <28 weeks), “very preterm” (VPT; 28 to <32 weeks) and “moderate to late preterm” (LPT; 32 to <37 weeks) subgroups [1]. In preterm infants, adequate nutrition early in life is an important factor for developmental outcomes such as neurodevelopment and later cognitive abilities. With greater survival rates, these outcomes have become more relevant as potential nutritional targets.

In this review, we focus on the impact of nutritional or dietary interventions initiated during hospitalization on brain and cognitive development in preterm infants, aiming to understand the impact of nutrients, nutritional supplements or dietary interventions on brain and cognitive development in preterm infants.

Compared to term born infants, preterm infants, especially those born with very low birth weight (VLBW, less than 1500 g) and extremely low birth weight (ELBW, less than 1000 g), have higher rates of brain damage and brain lesions [2], show decreased cortical gray matter volumes [2,3,4], alterations in subcortical structures, decreased microstructural connectivity [3,5,6], and different patterns of neuronal activation, for example regarding language processing much later in life [4]. However, such activation differences do not necessarily result in cognitive delays later in life, as the developing brain is still plastic and shows impressive compensatory abilities. For example, the use of alternative pathways has been demonstrated regarding language in preterm infants compared to term born infants studied at adolescence [7,8].

Cognitive outcomes in preterm infants depend on a variety of specific social, health and familial factors, including neurological outcomes. Therefore, VLBW and ELBW infants appear to be at a higher risk for cognitive delays and disturbances, with gestational age being an important factor [9,10]. In general, affected developmental domains in preterm infants include impaired language skills [4,11], memory [4,12] and executive functions [13]. Deficits often become more apparent during childhood and adolescence, with some causing long-term cognitive impairments. However, most disturbances are likely not domain-specific but rather indicative of impaired general cognitive performance [4]. In addition, higher rates of attention deficit and hyperactivity problems [14,15,16], emotional and socialization problems [4,17] have been reported in children and adolescents who were born preterm. Johnson and Marlow [16] suggest a “preterm behavioral phenotype”, particularly for EPT infants, characterized by an increased risk for symptoms and disorders associated with inattention, anxiety, and social difficulties. Cohort studies indicate those risks (that increase with decreasing gestational age at birth) to also have long-term social consequences, impacting education, jobs, family, and social security benefits in adult life [10].

As described cognitive and behavioral deficits may persist into school age and adolescence and may result in difficulties coping with adult life, early interventions are needed to reduce the risk for cognitive and social disturbances. One potential approach may be through nutrition, diet and feeding: The rapid growth of the developing brain during fetal and early post-natal life makes it particularly vulnerable to nutritional deficits. The effects of a deficiency or excess of a nutrient on brain and cognitive development depend on the timing, dose, duration of exposure and type of nutrient [18]. In the following sections, we review the findings from randomized and cohort studies.

## 2. Dietary Intervention Studies for Brain and Cognitive Development in Preterm Infants 

### 2.1. Enhanced Enteral and Parenteral Nutrition

One of the problems that require attention in preterm infants is extra-uterine growth failure. Adequate postnatal growth has been consistently associated with improved neurocognitive outcomes during childhood [19,20], adolescence [21] and adulthood [22,23,24], as well as with reduced risk of conditions such as cerebral palsy [25,26]. Therefore, early nutritional strategies that aim to limit body weight loss and promote growth are important and may positively impact neurodevelopment. Observational study results suggest that improving early parenteral and enteral nutrition to reduce energy and nutrient deficits may improve brain growth, brain maturation and neurodevelopment, especially in EPT and VPT infants. Even after adjusting for confounding factors such as infant gender, birth weight, co-morbidities and maternal education or socioeconomic status, greater cumulative intakes of energy, protein [27], and lipids [18] from both parenteral and enteral sources were associated with better neurodevelopment (as assessed by the Bayley Scales of Infant and Toddler Development (BSID) and Brunet-Lézine test) in these studies. Accordingly, two follow-up studies of the same randomized controlled trial (RCT) on the effect of an early (from less than 24 h of life) enriched parenteral and enteral nutrition on VLBW infant growth [28], showed enhanced white matter maturation at term-equivalent age [29] and improved visual perception at 52 weeks post-menstrual age [30]. The initial trial also showed that increased intake of fat, protein, long-chain polyunsaturated fatty acids (LCPUFA), vitamin A and phosphate resulted in enhanced body and head circumference growth, despite a higher frequency of late-onset sepsis in the intervention group [28]. By contrast, a previous RCT failed to demonstrate a significant effect of protein, fat and dextrose enriched parenteral and enteral feeds on brain volumes and cognitive outcomes (assessed by BSID) [31]. The large dropout rate in this trial (i.e., around 40% of the infants from both groups failed to complete the four-week intervention period) may explain the absence of significant effects. However, when the data from both groups were pooled, energy and protein deficits inversely correlated with brain volumes, mental and motor outcomes (assessed by BSID) at three months but not at nine months post-term [31].

In summary, the current evidence indicates some benefits of enriched enteral and parenteral nutrition in preterm infants, although the evidence from RCTs is limited. The three RCTs identified were small and did not report an appropriate power calculation for neurodevelopment; furthermore, in one study [31] the test administrator was not blinded to the feeding regimen. None of the studies defined neurodevelopment or cognition as the primary outcome.

For a summary of studies, please refer to Supplementary MaterialsTable S1.

### 2.2. Breast Milk and Infant Formula Studies 

For term-born infants, breast-feeding provides adequate nutrition to facilitate growth and development. However, the role of human milk in the development of preterm infants is less well defined as breast milk contains insufficient quantities of energy and nutrients to meet the greater nutritional needs of preterm infants. Compared with full-term infants, preterm neonates have high potential for growth but very limited nutrient reserves at birth. In addition, preterm infants are subject to a variety of physiological and metabolic stressors, such as infection, inflammation or respiratory distress, which increase their nutritional requirements [32]. The majority of preterm infants, especially very preterm infants, exhibit significant energy and nutrient deficits by the time they are discharged from the hospital [33].

Current data from two RCTs suggest that fortifying maternal breast milk for preterm infants with multi-nutrient human milk fortifier or preterm formula during the hospital stay [34,35] does not provide any significant advantage in cognitive development (measured by the Knobloch et al. Developmental Screening Inventory (BSID) [36] and Wechsler Intelligence Scale for Children (WISC)) or social maturity (measured by the Vineland Social Maturity Scale) at nine or 18 months, or eight years corrected age, compared to preterm infants fed with unfortified maternal breast milk. Both studies were performed by the same group nearly 20 years ago and should be reviewed critically. One study [34] compared feeding with unfortified breast milk to breast milk complemented with a multi-nutrient fortifier. However, milk represented only half of the feeds; the other half was preterm formula in both the fortified and unfortified groups, and the study was not powered for cognitive development as a primary outcome. In the second study [35], cognition was a priori defined as a primary outcome. Preterm or term formula was given when the mother was unable to provide enough milk, but the formula consumption in the study was actually low, as the mothers provided enough milk volume for most of the feeds. Therefore, more data are required before concluding any absence of benefit of breast milk fortification, a strategy that has been shown to have a beneficial impact on preterm growth and other health outcomes [37]. Furthermore, one of the studies suggested gender differences in the impact of preterm formula on cognitive outcomes, with a stronger effect in boys. Confounding factors may therefore be important to identify and consider in future studies.

More conclusive evidence comes from cohort studies comparing both fortified or unfortified maternal milk versus nutrient-enriched preterm formula, and unfortified maternal breast milk versus standard infant formula. Most of the available studies show breast milk to be advantageous for cognitive and behavioral outcomes (mainly assessed by BSID or Griffiths’ Mental Development Scales (GMDS)) [38,39,40,41,42,43,44,45,46], in line with the widely reported benefits of maternal milk on preterm infant health and development. However, none of the studies defined neurodevelopment or cognition as a primary outcome. Only one study failed to show a difference between breast milk and formula feeding for neurodevelopment outcomes (evaluated by BSID and Amiel-Tison Neurological Assessment) at 18 to 22 months of age, possibly due to the small sample size and the lack of statistical power as well as the relatively low contribution of breast milk volume (around 30%) to total infant intake in the milk fed group [47]. Obvious ethical reasons prevent the use of a randomized design. Although multivariate data analyses that include confounding variables confirm the benefits of maternal milk in most of the available studies [38,39,40,41,43,44], an uncontrolled confounder-related bias cannot be excluded in these studies.

In summary, evidence from association studies suggests a positive link between maternal breast milk and neurodevelopment in preterm babies that may last beyond early infancy. A significant association between the duration of breast milk feeding and cognitive abilities during infancy [41] as well as at school age [48] have been reported, suggesting benefits of extended breast-feeding for preterm children’s cognitive development. Prolonged breast-feeding after hospital discharge may thus be a mediating factor.

The benefits of donor milk on cognitive outcomes over formula are less clear, despite the fact that maternal milk and enteral feeding with donor milk have been reported to reduce infant morbidity and increase survival [49]. The three RCTs comparing unfortified banked milk to preterm formula were performed in the 1980s and early 1990s. One trial failed to find any significant difference in cognitive development as a primary outcome using BSID [50], while the other two studies showed an advantage for formula over donor milk on some secondary, short-term developmental outcomes assessed with the Knobloch et al. [36]. Developmental Screening Inventory [36] or Brazelton Neonatal Behavioral Assessment Scale (BNBAS) [51,52], with no differences in the longer term [50]. Pasteurization, which destroys neurologically active factors in donor milk, may be responsible for the poor impact of donor milk on cognitive outcomes. It is also possible that beyond the potentially bioactive molecules in the milk fluid, maternal milk provision during hospital stay can, by favoring the continuation of breast feeding after hospital discharge, promote the emotional maternal-infant bonding, a well-known positive factor for cognitive, behavioral and emotional development [53]. In any case, additional studies using the current protocols for infant clinical and nutritional management are warranted to confirm or refute these findings. To our knowledge, no studies have compared maternal to donor milk or fortified donor milk to preterm formula in terms of neurocognitive development.

Overall, the setup and quality of the donor milk RCTs can be considered good. The three studies reported appropriate randomization, blinding and similar group characteristics at baseline, although only one was adequately powered for neurodevelopment as a primary outcome [50].

For a summary of breast milk studies, please refer to Supplementary Materials Table S2.

Available evidence from four RCTs including nearly 600 infants consistently indicates that feeding preterm infants during their hospital stay with nutrient-enriched preterm formula compared to standard formula results in improved cognitive performance at 18 months corrected age (BSID) [54], as well as at eight and 16 years of age (WISC) [21,35,54,55]. This suggests that benefits of nutrient-enriched preterm formula may persist into childhood and adolescence. One potential factor for this may be the nutritional effect on early brain structural growth, as indicated by an explanatory neuroimaging study (MRI) by Isaacs et al. [55]. The study shows both a significantly higher verbal IQ and significantly larger caudate volumes. The composition of the different formulas used as interventional products in the studies above varied to some extent, but nutrient-enriched formulas were consistently higher in energy, protein, carbohydrate, lipids, vitamins and minerals than non-enriched formulas. The studies were of generally good quality, adequately randomized and blinded, but only two of the four studies [35,54] were statistically powered for neurodevelopment as a primary outcome, while the other two [21,55] did not report any specific power calculation. The study of Isaacs et al. [55] was an explanatory MRI study, so randomization and power calculation were not applied. Mediating factors of the effects in the formula studies included birth weight, duration of formula intake, and gender [21,54].

For a summary of formula studies, please refer to Supplementary Materials Table S3.

### 2.3. Studies with Specific Nutrients or Nutritional Supplements for Brain and Cognitive Development 

For an overview of nutrients and doses tested in the reviewed studies, please refer to Table 1.

#### 2.3.1. Protein and Amino Acids 

As previously mentioned, preventing weight-loss through adequate nutrition is one of the most important goals in the management of preterm infants during their hospital stay. The appropriate growth of lean body tissue, and the brain in particular, depends on neonatal protein intake [56]. Current evidence indicates that enteral intake ranging between 3 and 4 g/kg/day is safe and promotes adequate growth in preterm-born infants [57].

Most observational studies identified suggest a positive association between enteral and parenteral protein intake during the first 7–10 days of life and neurodevelopment in ELBW infants [27,58,59]. Similarly, two RCTs support a positive impact of enteral feed (breast milk or formula) fortification with protein doses of 3.8 g/kg/day [60] and 4.8 g/kg/day [55] compared to doses of 3.1 and 3.5 g/kg/day, respectively, on cognitive development, measured with developmental scales such as BNBAS and GMDS. In contrast, two follow-up studies in the 1970s [61,62] of a RCT from the 1960s [63] suggest that enteral supplementation with protein doses of 6 g/kg/day or more lead to an increase in detrimental outcomes, such as lower cognitive performance [62], fever and lethargy at 5–6 years of age. In addition to the dose, the assessment time points differ between the studies. Bhatia et al. [60] and Biasini et al. [56] report an early positive effect at 37 weeks and three months corrected age that did not remain significant at nine months corrected age. Of note, none of the identified trials report a power calculation for the neurocognitive outcomes.

In summary, results from the reviewed studies are in line with findings on physical growth [57]: an enteral intake of protein of over 3.5 g/kg/day would be required to support proper neurodevelopment in preterm-born infants, whereas intakes over 6 g/kg/day may have detrimental effects. More studies are required to confirm these results, define the optimal enteral protein dose, and assess the long-term impact on infant cognitive outcomes.

Two parenteral amino acid (AA) supplementation studies failed to show neurodevelopmental benefit as measured by the BSID at 6, 12, 18, or 24 months corrected age [64,65]. In both studies, supplementation was in the range of 3 to 4 g/kg/day, the target population was ELBW infants, and cognition was a secondary outcome. The study by Poindexter et al. [65] compared timing (early versus late provision of AAs), whereas Blanco et al. [64] compared AA doses (3 versus 4 g/kg/day). The latter demonstrated a significantly lower mental development index (BSID) score at 18 months for the early and high AA doses, but the difference was no longer evident at 24 months. In a third study, the effect of parenteral AA intervention (supplementation from birth with 2.4 g/kg/day versus a standard, more gradual AA introduction) on neurodevelopmental outcomes was gender- and outcome-dependent. While the frequency of major disabilities at two years of age (primary outcome) was smaller in boys in the intervention group, the Mental Development Index of the BSID (secondary outcome) was lower in girls in the intervention group than in the standard AA introduction group [66].

Taurine is a conditionally essential, non-protein AA in preterm infants. It is found at very high levels in neural tissue, particularly in the developing brain. It has therefore been postulated that adequate intake of taurine in the preterm infant is required to support brain development. Consistent with this, low taurine status during the neonatal period has been associated with impaired cognitive performance during childhood, i.e., lower scores on the BSID mental development index at 18 months and the WISC-R arithmetic subtest at seven years [67]. In addition, another RCT showed that taurine supplementation of pre-discharge formula (45 mg/L) versus non-supplemented formula (<5 mg/L) had a mild but positive impact on maturation of auditory brainstem-evoked responses in preterm infants [68]. However, both studies reported no further differences in cognitive, neurobehavioral or eye development outcomes (BNBAS and electroretinogram, ERG) compared to non-supplemented formula.

The supplementation effect of glutamine, another AA proposed as conditionally essential in preterm infants, has been investigated by two follow-up studies on cognition and one on brain development from a RCT [69] that originally investigated enteral glutamine supplementation in VLBW preterm infants at 0.3 g/kg per day for four weeks soon after birth. The follow-up studies on cognition indicate no particular effect on neurodevelopment (motor, cognitive and behavioral) at two [70] and 7.5 [71] years of age, despite a lower incidence of serious neonatal infections and increased brain volumes at 8.5 years [71] in the glutamine group than the alanine control group [70,71]. Current evidence is too scarce to draw any conclusions; the reviewed studies suggest no cognitive benefit of glutamine supplementation during the neonatal period in VLBW preterm infants.

None of the protein or AA studies defined neurodevelopment or cognition as a primary outcome, nor were they powered for it. For a summary of studies, please refer to Supplementary Materials Table S4.

#### 2.3.2. Long-Chain Polyunsaturated Fatty Acids

LCPUFAs are important components of the cell membranes in the human brain and retina, with docosahexaenoic acid (22:6*n*-3; DHA) and arachidonic acid (20:4*n*-6; ARA) being the major *n*-3 and *n*-6 LCPUFAs, respectively, deposited in the membranes of the developing brain and retinal photoreceptor cells during the perinatal period. In utero, the placenta selectively and substantially extracts DHA and ARA from the mother and enriches the fetal circulation. Most intrauterine DHA and ARA accumulation occurs during the last trimester of pregnancy [72]. The physiological requirements for DHA and ARA are highest during the perinatal period. Prematurely born infants are at particular risk for LCPUFA deficiency; they lose their normal placental supply of nutrients, have very low fat stores, and have to rely on immature digestive and metabolic functions [73,74]. LCPUFA supplementation of breast milk and formula has been suggested to improve both cognitive and visual development in these infants.

*Cognitive development.* Among the nutrients in the present review, LCPUFAs were the most widely investigated, with 13 intervention RCTs and three observational studies. However, evidence from these studies was mixed, making it difficult to draw unambiguous conclusions.

Two of the intervention studies from the same infant cohort suggest LCPUFA supplementation of human milk during hospital stay has a beneficial effect on short-term neurodevelopmental outcomes, e.g., including problem-solving skills, recognition memory, and attention scores assessed with the Ages and Stages Questionnaire (ASQ), the primary outcome in both studies, and free play sessions compared to unfortified human milk [75,76]. However, a recent follow-up study failed to find any significant effect of LCPUFA supplementation on brain volumes and cognitive outcomes, including Wechsler Abbreviated Scale of Intelligence (WASI), Wechsler Intelligence Scale for Children (WISC-III), California Verbal Learning Test (CVLT-II) and the Grooved Pegboard test in these children at eight years of age [77]. Positive findings have been reported for supplemented formula (i.e., double dose of fish oil or higher levels of DHA and ARA) versus non-supplemented formula (i.e., single dose of fish oil; regular levels of LCPUFAs), during hospital stay only [78], as well as before and beyond hospital discharge [73,79]. However, none of the three studies reported a sample size calculation for the cognitive outcomes (BSID). Quite a few intervention studies, on the other hand, do not support significant neurodevelopment benefits (e.g., measured by BSID), either comparing supplemented versus non-supplemented infant formula [80,81], mixed feeding [82,83,84,85], or increased levels in breast milk due to maternal supplementation [86]. Most of the studies investigated cognitive development as the primary outcome and were adequately powered for it, except for those by Isaacs et al. [84] and van Wezel-Meijler et al. [81]. Factors that may have contributed to these controversial and inconclusive results include gender-specific effects [83,84,86], degree of immaturity [85], type of infant nutrition tested (maternal breast milk versus formula or donor milk), essential fatty acid supply and status, time of supplementation, and oil source, as well as a large range of LCPUFA doses. Most of the available studies tested doses equal to or lower than 0.35% DHA. Higher doses (i.e., 1.4% of the fatty acids as DHA) showed a short-term benefit [75,76], which did not seem to persist into later childhood [77].

Evidence from the two observational studies [87,88], both based on the same cohort, found significant negative associations between Mead acid levels, EFA deficiency index in mother’s breast milk, and infant motor quality (General Movements) at 40 weeks of gestational age, and between ARA levels in breast milk and BNBAS outcomes at 40 weeks of gestational age [87], as well as between *n*-6 concentration in breast milk and motor, mental and behavioral development (General Movements and BSID) up to 18 months of age [87]. A third, smaller observational study comparing infants who received DHA supplemented parenteral feeds during the first 3-4 weeks with a historical, unsupplemented cohort failed to identify any difference in neurodevelopmental outcomes (assessed by BSID and Vocabulary Test-Comprehension of the Peabody Picture Vocabulary Test) at three years of age [89]. Neither of the observational studies defined a primary outcome, nor did they report a power calculation for the cognitive and behavioral endpoints.

In summary, the body of evidence linking early nutrition and cognitive benefits in preterm infants is probably largest for LCPUFA. The quality of the intervention studies was generally good to high, as most were adequately randomized, blinded and powered for cognitive outcomes. Many of the studies in this area explored neurodevelopment and cognition as the primary outcomes. Nevertheless, more studies are needed to determine the optimal LCPUFA dose, LCPUFA combinations, ratios and possibly oil sources, timing and length of the intervention, as well as the preterm infant populations that may benefit the most.

*Visual development.* Inconsistent findings have been reported for LCPUFAs and visual development in 14 RCTs. Studies vary greatly in design, particularly regarding nutritional intervention (different combinations of LCPUFAs, diets, doses and different control diets) and all except one lack power calculation for visual development outcomes. Only the study by Smithers et al. [90] defined visual development as a primary outcome. Interventions during the hospital stay showed some positive findings for retinal function as assessed by ERG [91] but not for visual acuity [73,81,92,93] when comparing different infant formula groups; however, positive effects on visual acuity were yielded with mixed feeding [85,90]. Interventions that started during and continued beyond the hospital stay resulted in mixed findings with some supportive evidence for DHA- and/or eicosapentaenoic acid (EPA)-supplemented infant formula for visual acuity [92,93,94,95,96], visual information processing speed [97,98], and in some electrophysiological measures (i.e., visual evoked potentials) [99], but not for retinal function [100], or brain auditory evoked potentials [99] in preterm infants.

Overall, supplementing the diet of preterm infants with a combination of LCPUFAs (ARA, DHA, EPA) rather than with single LCPUFA types appears to be most beneficial for visual outcomes, at least when compared to a diet low in *n*-3 fatty acids. However, observational data do not support associations between LCPUFA levels in breast milk and retinal function or visual acuity in very preterm infants [74]. Similar to the cognitive outcome studies, the visual development studies showed a high heterogeneity in terms of type of population, type of infant nutrition, essential fatty acid supply or LCPUFA doses. High doses of DHA may be effective (i.e., 1.0% fatty acid DHA), although only one study testing this dose was found [90].

For a summary of LCPUFA studies, please refer to Supplementary Materials Table S5.

#### 2.3.3. Micronutrients 

*Vitamin A.* Adequate vitamin A status is key for optimal growth and development. Preterm infants are often deficient in vitamin A, especially extremely preterm infants, due to very limited liver stores at birth and possibly insufficient supplementation during and after hospitalization [101]. Vitamin A deficiency may increase the risk for bronchopulmonary dysplasia (BPD, a neonatal chronic lung disease), retinopathy of prematurity (ROP) [101], and long-term neurodevelopmental delays [102].

Only one publication was identified that investigated the effect of intramuscular vitamin A supplementation (i.e., 5000 IU, three times per week) versus sham injection in ELBW infants during hospitalization. It suggests a reduction of BPD symptoms but no significant effect on neurological, mental or psychomotor development, as measured by neurodevelopmental impairment (NDI) and BSID [103]. The study publication does not report a power calculation in the trial design.

*Iron.* Iron deficiency is the most common single nutrient deficiency worldwide, and preterm infants are at particular risk because of their small iron store at birth, high growth velocity, and the iron losses caused by frequent blood sampling [104]. Iron plays an important role in the development of the central nervous system and is essential to neural myelination and neurotransmitter function. Iron deficiency anemia during infancy is associated with poor neurological development [105,106]. However, it is important to state that there are some concerns regarding iron supplementation because humans have no system for iron excretion; since preterm infants are particularly vulnerable to oxygen radical injury, iron supplementation can be harmful for the preterm brain [105,106].

Results from two RCTs at best suggest that fortifying enteral nutrition of preterm infants with 1–2 mg iron/kg/day from early life may improve some neurodevelopmental outcomes such as gross motor functioning (evaluated by the Gross Motor Function Classification System (GMFCS)) and intellectual abilities (evaluated by The Kaufman Assessment Battery for Children (K-ABC)) [106], as well as behavioral outcomes (e.g., as assessed with the Child Behavior Checklist (CBCL)) at pre-school age [107]. In contrast, no improvement in hearing nerve responses (Central Conduction Time) [105] or in cognitive performance assessed with the WPPSI (Wechsler Preschool and Primary Scale of Intelligence) at pre-school age [107] could be demonstrated with these iron doses. An intervention with higher doses, e.g., >3 mg iron/kg/day, does not appear to add any beneficial effects on motor and neurological development compared to 2 mg/kg/day [108]. Thus, overall, iron-fortified nutrition providing 1-2 mg iron/kg/day from early life on may be enough to improve cognitive development in preterm infants. The evidence is based on three properly randomized and blinded studies, of which two were adequately powered for hearing nerve response and cognition as primary outcomes. The study by Steinmacher et al. [106] investigated cognitive and behavioral development as secondary outcomes, focusing on iron status and iron deficiency as a primary outcome.

#### 2.3.4. Probiotics 

Oral administration of probiotics has been shown to be effective in reducing the incidence of necrotizing enterocolitis (NEC) in preterm-born infants. It has also been proposed that oral probiotics may improve gastrointestinal function, leading to less feeding intolerance [109]. This enhances nutrition during the neonatal period, which may contribute to improved growth as well as neurodevelopmental outcomes. In addition, recent developments in the area of the microbiota-gut-brain axis [110] suggest that modulation of the gut microbiota composition and/or metabolism via probiotic interventions may have an impact on brain development, and consequently on neurocognitive outcomes.

While the findings from one unblinded RCT suggest that oral administration of either *Lactobacillus rhamnosus* ATCC 53103 or *Lactobacillus reuteri* ATCC 55730 may help to prevent suboptimal neurological outcomes [111] in very preterm infants, the results of two other RCTs with *Lactobacillus sporogenes* [112] or the blend Infloran (*Lactobacillus acidophilus* and *Bifidobacterium infantis*) [113] did not support an effect of these strains for neurological (Hammersmith Infant Neurological Examination (HINE)) and neurodevelopmental outcomes (BSID and NDI). Only the two latest studies [111,112] were powered for the NDI as a primary outcome. More studies are required to confirm these results and to understand the potential impact of specific probiotic strains and doses.

#### 2.3.5. Prebiotics

Similar to probiotics, prebiotics can modulate the gut microbiota of preterm infants and reduce the levels of pathogenic bacteria [114]. However, to date RCTs have failed to demonstrate a benefit of prebiotic oligosaccharides on clinical outcomes such as NEC, sepsis or tolerance to enteral feeding; for review see Srinivasjois et al. [115]. Similarly, the only prebiotic RCT identified failed to demonstrate a beneficial effect of breast milk or preterm formula supplementation with a blend of neutral and acidic oligosaccharides on neuromotor development during the first year of life [116]. This study was a follow up of a blinded RCT monitoring the impact of prebiotics on infectious morbidity [117] and was not powered for neurodevelopmental outcomes.

#### 2.3.6. Sphingomyelin

Sphingomyelin is an important lipid in the structure of brain membrane cells. Only one randomized pilot study [118] has investigated the effect of sphingomyelin supplementation in a few very preterm infants so far. Findings reported some improved neurodevelopmental outcomes, including intellectual abilities (Fagan Test of Infant Intelligence), sustained attention (free play) and behavior (BSID) with formula containing 20% (of total phospholipids) sphingomyelin from milk phospholipids compared to 13% sphingomyelin from egg phospholipids. The intervention was for eight weeks immediately after birth [118]. Again, more studies are needed to validate these findings and to understand whether increased sphingomyelin doses and/or the use of milk as source of this compound could be responsible for the observed effect.

For a summary of micronutrients, probiotics, prebiotics and sphingomyelin studies, please refer to Supplementary Materials Table S6.

## 3. Discussion 

Nutritional interventions starting during hospitalization to reduce the risk of neurodevelopmental, cognitive and behavioral disturbances in preterm infants have been reviewed in this paper.

### 3.1. Nutrients, Nutritional Supplements and Dietary Interventions That Have Been Shown to Be Promising for Neurodevelopmental Effects in Preterm Infants

In general, mother’s breast milk has been shown to be superior to formula milk. This is consistent with a systematic review by Koo et al. [119] on the effect of human milk feeding on neurodevelopment outcomes in preterm VLBW infants. The review highlights that preterm VLBW children with no neurological impairment receiving human milk scored within normal ranges on standardized cognitive tests (BSID, K-ABC, and WISC). Limited evidence points towards no significant advantage of fortified over unfortified breast milk. By contrast, currently available studies do not support the superiority of unfortified donor milk over preterm formula on neurodevelopment. Whether fortified donor milk is superior to formula in terms of neurodevelopmental outcomes has not yet been studied, to our knowledge. Furthermore, nutritionally enriched preterm formula was largely shown to be superior to non-enriched term formula. This is consistent with a recent systematic review and meta-analysis by Chan et al. [120], which concluded that increased early enteral nutrition may increase the likelihood of survival without neurodevelopmental impairment in VPT and/or VLBW infants. However, the authors also highlight significant heterogeneity between study designs (*I*^2^ = 75.9%). It should also be noted that most nutrient-enriched formulas used in the reported studies supply consistently high energy, protein, carbohydrate, lipids, vitamins and minerals, as the majority of preterm infants, especially VPT, exhibit significant energy and nutrient deficits by the time they are discharged from the hospital [33]. The high supply of energy, although often linked to positive neurodevelopmental outcomes [27], may also result in undesirable side effects such as electrolyte deviations during the first week of life [28]. Other studies suggest an insignificant contribution of mean energy intake/kg/day (as compared to birth weight for example) to growth outcomes in the first year of life of preterm infants [121].

Regarding single nutrients or nutritional supplements, the variety of study designs and methods used make it difficult to give overall nutritional recommendations. Probably the most consistent positive results based on a few studies are for enteral protein and iron, though confirmatory research is required, as recommended in two recent Cochrane systematic reviews evaluating the evidence on these nutrients [57,122]. A positive impact has been also reported for the probiotics *L. rhamnosus* ATCC 53103 and *L. reuteri* ATCC 55730 (one positive study), and Sphingomyelin (one positive pilot study), although more data based on adequately powered studies are warranted to confirm these findings. Nutrients shown to be ineffective in the scope of the current review, on the other hand, include glutamine, vitamin A, parenteral AA, taurine, prebiotics (blend of neutral and acidic oligosaccharides) and the probiotics *L. sporogenes* and Infloran (*L. acidophilus* and *B. infantis*), although the number of trials investigating each of these compounds is far too low to conclude absence of benefit. In addition, only the two probiotic studies were adequately powered for a neurodevelopmental outcome (assessed by NDI), while the AA (parenteral, glutamine, and taurine), vitamin A and prebiotic studies either reported no primary outcome and/or power calculation or included cognition as a secondary outcome. The most studied nutrient group is LCPUFA, but inconsistent results have been found for both cognitive and visual outcomes. While most of the included studies have tested levels equal to or below 0.35% DHA and report inconsistent results, three studies with high doses of DHA yielded some short-term effects, two for cognition (i.e., 1.4% fatty acid DHA) [75] and one for vision (1.0% fatty acid DHA) [90]. However, the long-term sustainability of these effects remains to be demonstrated [77]. The lack of consistent efficacy for cognition is in line with the recent meta-analysis by Qawasmi et al. [123], which found no significant effects of LCPUFA supplementation on cognition in preterm infants. The analysis did not find dose effects for the whole population evaluated in the meta-analysis, which included term as well as preterm-born infants. Regarding visual acuity, a meta-analysis by the same group [124], again including both term and preterm-born infants, found that trials assessing visual acuity by using visual evoked potentials (VEP) tended to show a positive effect of supplementation, while trials that used doses of <0.32% of DHA were likely to show no significant effect on visual acuity. A moderating effect of preterm status on the association between LCPUFA supplementation and visual acuity was reported; however, the lack of consistency led the authors to discuss it as a false positive error. Results indicating efficacy of high LCPUFA doses for neurodevelopmental outcomes are in line with the emerging view that the LCPUFA requirements of preterm infants may be larger than previously estimated [125]. From this perspective, early LCPUFA supplementation with doses above the current recommendations may make it possible to match the LCPUFA fetal accretion rates, leading not only to improved cognitive and visual outcomes but also to reduced risk of preterm mortality and morbidity [126]. Nevertheless, additional studies are warranted to confirm the efficacy and ensure the safety of this approach.

### 3.2. Study Design, Expected Sample Sizes and Outcomes for Nutritional Clinical Trials among Preterm Infants to Assess and Demonstrate Changes in Neurodevelopment and Cognition

In general, there was huge variability in methods. Sample sizes of RCTs ranged from 20 to more than 600 infants. Neurocognitive development was often not the primary objective of the studies and many trials were inadequately powered for cognition-related outcomes. Comparison of the results from different trials was limited by, among other factors, the use of inclusion criteria based on either gestational age or body weight at birth and also by selecting infants with differing degrees of prematurity and/or birth body weight. Based on the reviewed studies, some factors could be identified that are highly relevant and should be considered as potential confounding or stratifying variables for future studies. These include degree of prematurity and birth weight, postnatal age at start of intervention (e.g., a few hours versus several days or weeks after birth), duration of intervention, type of infant nutrition, and length of follow-up visits (thus drop-out rate), breast-feeding duration after hospital discharge, and gender differences. The supply of energy and other nutrients during and after the intervention likely plays an important role as well, both in positively modulating neurodevelopment rates as well as in potentially leading to undesirable side effects. In addition, well-known factors such as neonatal risk, sequelae of perinatal care and risks for long-term consequences [10], socioeconomic status or maternal IQ should of course be appropriately addressed in a study design.

*Cognitive-behavioral outcomes.* The majority of the studies assessed neurodevelopment by developmental screening tools, such as the BSID, GMDS, Knobloch et al. Developmental Screening Inventory [36], and Wechsler scales. These development tests yield overall development scores (i.e., DQ, Development Index, or IQ), which are rather robust within normal ranges, as they test a child’s abilities across several developmental domains (i.e., language, motor, cognitive, and emotional skills), and resulted in very mixed findings across studies. In the context of nutritional efficacy studies, developmental screening measures display a relatively low degree of sensitivity [127], which in turn impacts interpretability and relevance of the research findings [128]. Especially in children, those cognitive tests measure behavior or performance in a specific situation rather than actual intellectual ability or competence [129], therefore interpretation of the study findings may be limited, especially across the variety of measures used in the included studies. However, analyses of more specific subcategories such as verbal intelligence or language skills seem more promising for investigating the impact of nutrition and diet on brain and cognitive development in preterm children. In line with ILSI Europe recommendations [127,128], this suggests and supports more hypothesis-driven approaches for nutritional interventions, targeted at the neurodevelopmental or cognitive domain that is expected to be impacted by the intervention, based on known or hypothesized mechanisms of action, especially for studies investigating individual nutrients or compounds. Furthermore, measures derived from MRI and EEG techniques may support more objective and specific assessments of brain benefits in nutritional studies than traditional developmental screening tools.

*Neuroimaging and electrophysiological outcomes.* Although neuroimaging provides a unique way of insight into in vivo brain development and is routinely used in preterm infants in clinical settings, few studies included neuroimaging-based outcomes. Six studies included MRI outcomes [29,31,55,71,77,81] with mixed findings. Some dietary effects on brain volumes (e.g., caudate structures, white matter, and hippocampus) and diffusivity measures (e.g., in corticospinal tract and cingulum) were reported for enriched parenteral nutrition with neuroimaging at term-equivalent age [29], glutamine supplementation with neuroimaging at 8.5 years of age [71] and high nutrient preterm formula with neuroimaging at 16 years [55]. No nutritional effects, however, were reported for enriched parenteral nutrition on total brain volume at 40 weeks postconceptional age [31], parenteral LCPUFA on white matter in infants at three and 12 months corrected age [81], or for enteral LCPUFA with neuroimaging at 8.5 years of age [77]. Nine of the reviewed studies included at least one electrophysiological outcome such as event-related potentials (ERP), VEP, auditory brainstem responses (ABR) [109], and electroretinogram (ERG). These measures were found predominantly in the context of LCPUFA and visual development and resulted in mixed findings [74,90,91,92]. These measures seem to be valuable to assess, and in some cases visualize, brain physiological changes in vivo.

*Non-nutritional interventions.* Interestingly, none of the studies reviewed here investigated the complementary effect of non-nutritional or behavioral interventions in combination with nutrition. In vulnerable populations, e.g., undernourished children, this appears a promising intervention strategy to impact brain and cognitive development [130]. Therefore, combined or holistic intervention approaches around nutrition could be an interesting area to be explored in future studies in preterm infants. This may also better reflect the multi-etiology of child development, which is influenced by genetic factors, individual factors (temperament/ personality), biological factors (e.g., health and nutritional status), environmental factors (e.g., stimulation, and quality of mother-child interaction) and cultural factors, with nutrition being only one aspect, albeit an important one.

## 4. Conclusions

To summarize, many clinical studies have investigated the benefit of early nutritional interventions for neurodevelopment and cognition in preterm infants. Maternal breast milk seems to be best for preterm infant neurodevelopment, as it is for many other preterm outcomes. Although for obvious ethical reasons maternal milk studies were all observational, most of them were well controlled for confounders related to infant risk (e.g., gender, gestational age, birth weight, and length of hospitalization) and comorbidities known to impact neurodevelopment (e.g., NEC and BPD), as well as maternal and family factors (e.g., socioeconomic status, education, and smoking). These findings are in clear contrast to those obtained from the randomized comparison of donor milk and preterm formula, which found unfortified banked milk to be disadvantageous for neurodevelopmental outcomes. The results suggest that biologically active components in unprocessed maternal milk that may be responsible for improved cognitive outcomes may be destroyed by donor milk pasteurization. Alternatively, confounding effects of non-nutritional factors associated with breastfeeding (e.g., skin contact, bonding, and maternal well-being), especially after hospital discharge, may also explain the benefits of maternal milk feeding [53].

The methodological variety of study designs makes it difficult to conclude any effect of interventions with individual nutrients. Only protein and iron level results show some consistency. Surprisingly, this review does not support scientifically established dogma that are widely accepted, such as LCPUFA supplementation of preterm formula for visual development when an adequate supply of essential fatty acids is provided. To strengthen the estimation of the intervention effects, meta-analyses for defined nutrients or nutritional approaches, e.g., LCPUFAs or amino acids, may be desirable next steps, particularly for those areas where existing individual studies are not adequately powered.

Some relevant factors that appear to determine the success of an intervention study in preterm infants include: the type of infant nutrition tested, the timing of the nutritional intervention (it seems safe to state that the earlier the intervention, the better the effect), and the dose of the nutrient/ingredient. However, higher doses do not necessarily lead to better benefits. Doses should be considered carefully for this vulnerable population due to potential toxic effects and the risk for adverse outcomes.

Suggestions for the design of future clinical trials are to test subcategories (e.g., verbal IQ instead of full IQ scales) or specific domains (e.g., motor skills, language skills, and attention skills) of neurodevelopment and cognition, based on clear hypotheses, rather than overall development scores. Moreover, gender differences and degree of infant prematurity should be considered, especially when considering these subcategories of development and specific cognitive domains.

In conclusion, preterm-born babies need proper support for brain and cognitive development. One of the influencing early life factors may be nutrition, with maternal breast milk being best for neurodevelopment in preterm infants. When maternal breast milk is not available, fortified preterm formula has been shown to be an alternative from the currently available scientific evidence. Additional studies comparing the neurodevelopmental effects of fortified donor milk to preterm formula are warranted. Until such results are available, donor milk should remain the preferred choice when maternal milk is not available, given the superiority of donor milk over formula in terms of tolerance to feeding and risk of NEC; this is supported by a systematic review by Quigley and McGuire [49]. In general, more and better quality studies are needed on the topic; the currently best investigated nutrients in many good quality studies are LCPUFAs.

## Figures and Tables

**Table 1:** Doses, feeding mode and paradigm of interventions with specific nutrients and nutritional supplements for brain and cognitive development in preterm infants

Nutrient	*N* Trials	Doses Tested	Doses Reported as Effective	Feeding Mode	Intervention Paradigm
**Protein**	4	2.6–7.2 g/kg/day	3.8–4.8 g/kg/day	Breast milk or formula	from 100 Kcal/kg/day during 2 weeks; from full enteral feeds until discharge, depending on the study
**Amino acids**	3	0–4.0 g/kg/day	Uncertain	Parenteral solution	from <2–72 h after birth during 3–20 days, depending on the study
**Taurine**	1	<5 vs. 45 mg/L	None	Formula	from 7–10 days after birth until discharge
**Glutamine**	3	0 vs. 0.3 g/kg/day	None	Breast milk or formula	from 7 to 30 days after birth
**LCPUFA:** *cognitive development*	13	DHA: 0%–1.4% FAARA: 0%–1.2% FA	Uncertain	Breast milk or formula	from between birth and 10 weeks of life until either discharge or 12 months of CA, depending on the study
**LCPUFA:** *visual development*	14	DHA: 0%–1.0% FAARA: 0%–0.68% FA	Uncertain	Breast milk or formula	from between <72 h and 25 days of life until either discharge or 12 months of CA, depending on the study
**Vitamin A**	1	5000 IU	None	Not reported	Intramuscular injection; 3 times/week; from birth to 4 weeks of age
**Iron**	3	1–3.4 mg/kg/day	1–2 mg/kg/day	Breast milk or formula	from between 2 and 9 weeks of age until between 6 months and 12 months after discharge, depending on the study
**Probiotics**					
***L. reuteri* ATCC 55730**	1	1 × 10^8^ cfu/day	1 × 10^8^ cfu/day	Not reported	from <72 h after birth until discharge
***L. rhamnosus* ATCC 53103**	1	2.6 × 10^9^ cfu/day	2.6 × 10^9^ cfu/day	Not reported	from <72 h after birth until discharge
***L. sporogenes***	1	3.5 × 10^8^ cfu/day	None	Predominantly formula	from <48 h after birth until discharge
**Infloran**	1	2 × 10^9^ cfu/kg/day	None	Breast milk	until discharge (intervention start not reported)
**Prebiotics (80% _SC_GOS/_LC_FOS + 20% pAOS)**	1	≤1.5 g/kg/day	None	Breast milk or formula	from 3 days after birth during 28 days
**Sphingomyelin**	1	13% and 20% total phospholipids	20% total phospholipids	Predominantly breast milk	from <24 h after birth during 8 weeks

LCPUFA: long-chain polyunsaturated fatty acids; _SC_GOS: short-chain galacto-oligosaccharides; _LC_FOS: long-chain fructo-oligosaccharides; pAOS: peptin-derived acid oligosaccharides; DHA: docosahexaenoic acid; ARA: arachidonic acid; FA: fatty acids; IU: international units; cfu: colony forming units; CA: corrected age.

## Supplementary Materials

The following are available online at http://www.mdpi.com/2072-6643/9/3/187/s1, Table S1: Enhanced Parenteral and Enteral Nutrition Studies, Table S2: Breast Milk Studies, Table S3: Infant Formula Studies, Table S4: Protein and Amino Acid Studies, Table S5: LC-PUFA Studies, Table S6: Micronutrient and Specific Ingredient Studies.

## Abbreviations

AA	Amino acids
ARA	Arachidonic acid
ELBW	extremely low birth weight (less than 1000 g)
EPT	extremely preterm (<28 weeks)
LPT	late preterm (32 to <37 weeks)
VLBW	very low birth weight (less than 1500 g)
VPT	very preterm (28 to <32 weeks)
