# Supplementary material for: Early Nutritional Interventions for Brain and Cognitive Development in Preterm Infants: A Review of the Literature
Source: Nutrients. 2017 Feb 23;9(3):187. doi: 10.3390/nu9030187 (PMC5372850; doi:10.3390/nu9030187)
Supplement: Supplementary file 1 [file nutrients-09-00187-s001.docx]

**Early nutritional interventions for brain and cognitive development in preterm infants: a review of the literature**

Clinical trials and cohort studies with nutritional interventions starting during hospital stay of preterm infants

TABLE OF CONTENT

1. SUPPLEMENTARY TABLE 1. ENHANCED PARENTERAL AND ENTERAL NUTRITION STUDIES
2. SUPPLEMENTARY TABLE 2. BREAST MILK STUDIES
3. SUPPLEMENTARY TABLE 3. INFANT FORMULA STUDIES
4. SUPPLEMENTARY TABLE 4. PROTEIN AND AMINO ACID STUDIES
5. SUPPLEMENTARY TABLE 5. LC-PUFA STUDIES
6. SUPPLEMENTARY TABLE 6. MICRONUTRIENT & SPECIFIC INGREDIENT STUDIES

**ABBREVIATIONS**

| AA | Amino acid | ITQ-R | Infant Temperament Questionnaire – Revised |
| --- | --- | --- | --- |
| ARA | Arachidonic acid | IVN | Intravenous Nutrition |
| ABR | Auditory Brainstem Response | K-ABC | The Kaufman Assessment Battery for Children |
| AIMS | Alberta Infant Motor Scale | Knobloch et al. | Developmental Screening Inventory by Knobloch, Passamanick, & Sherrard |
| Amiel-Tison | = Neurological assessment method for newborn infants | KRISP | Kansas Reflection-Impulsivity Scale for Preschoolers |
| ASQ | Ages and Stages Questionnaire | LBW | Low Birth Weight |
| BAEP | Brainstem acoustic evoked potential (see also ABR) | LCPUFA | Long-Chain Polyunsaturated Fatty Acids |
| BHM | Banked Human Milk | LOS-KF 18 | The Lincoln-Oseretzky Scale-Kurzform (Short Form) 18 |
| BL | Brunet-Lézine | MABC | Movement Assessment Battery for Children |
| BNBAS | Brazelton Neonatal Behavioral Assessment Scale | MCDI | The MacArthur Communicative Development Inventory |
| BPD | Bronchopulmonary Dysplasia | MDI | Mental Development Index (of BSID) |
| BRS | Behavior Rating Scale (subtest of BSID-II) | MPC | The Mental Processing Composite (of K-ABC) |
| BSID | Bayley Scales of Infant and Toddler Development | MRI | Magnetic Resonance Imaging |
| BW | Birth Weight | NCV | Peripheral Nerve Conduction Velocity |
| CA | Corrected Age | NDI | Neurodevelopmental Impairment |
| CBCL | Child Behavior Checklist | NEC | Necrotizing Enterocolitis |
| CBV | Cortical Brain Volume | NICU | Neonatal Intensive Care Unit |
| CCT | Central Conduction Time | PDBD | Parent-rated Disruptive Behavior Disorders questionnaire |
| CMS | Children’s Memory Scale | PFF-T | Predominantly Formula Fed until Term |
| CNS | Central Nervous System | PHM-T | Predominantly Human Milk Fed until Term |
| CP | Cerebral Palsy | PIQ | Performance IQ (in WAIS-III, WISC-III and WISC-R) |
| CRIB | Clinical Risk Index for Babies | PMA | Post Menstrual Age |
| DHA | Docosahexaenoic Acid | PN | Parental Nutrition |
| DQ | Developmental Quotient | RCT | Randomized Controlled Trial |
| EEG | Electroencephalogram | SDQ | Strengths and Diﬃculties Questionnaire |
| EFA | Essential Fatty Acids | STSC | Short Temperament Scale for Children |
| ELBW | Extremely Low Birth Weight | SQ | Social Quotient (of Vineland Social Maturity Scale) |
| EN | Enteral Nutrition | TAC | Teller Acuity Card procedure |
| EPA | Eicosapentaenoic Acid | TBV | Total Brain Volume |
| ERG | Electroretinogram | TDBD | Teacher-rated Disruptive Behavior Disorders questionnaire |
| ERP | Event Related Potentials | TRF | The Teacher’s Report Form (of CBCL) |
| Fagan test | The Fagan Test of Infant Intelligence | VEP | Visual Evoked Potential |
| FPL | Force-choice Preferential-looking | VIQ | Verbal IQ (in WAIS-III, WISC-III and WISC-R) |
| FSIQ | Full Scale Intelligence Quotient (in WAIS, WISC, WISC) | VLBW | Very Low Birth Weight |
| GA | Gestational Age | WAIS | Wechsler Adult Intelligence Scale |
| GMDS | Griffiths’ Mental Development Scales | WASI | Wechsler Abbreviated Scale of Intelligence |
| GM | General Movements | WIAT | Wechsler Individual Achievement Test |
| GMFCS | Gross Motor Function Classiﬁcation System | WISC(-R) | Wechsler Intelligence Scale for Children (Revised version) |
| HINE | Hammersmith Infant Neurological examination | WPPSI | Wechsler Preschool and Primary Scale of Intelligence |
|  |  |  |  |

TABLE S1. ENHANCED PARENTERAL AND ENTERAL NUTRITION STUDIES.

| Author, year, country, design | Study population | Intervention diet  (dose and formulation) | Control diet  (dose and formulation) | Intervention/ Observation period | Outcome measures | Effect of the intervention/  Findings of cohort study |
| --- | --- | --- | --- | --- | --- | --- |
| Tan et al 2008  UK  RCT | Sample:  VLBW infants, GA<29 weeks in NICU, free from major congenital abnormalities  Age & Weight:  Median values in intervention group: 915g and 27 weeks;  Median values in standard feeding group: 980g and 27 weeks  Randomized:  142; 68 in intervention and 74 in standard feeding group  Followed up:  81/142 (57%) had BSID-II at 3 mo;  71/142 (50%) had BSID-II at 9 mo;  65/142 (46%) had MRI scan at 40 weeks PMA | Dose:  Parenteral nutrition (PN) increased stepwise from 1 g amino acids and fat/kg/day to 4 g protein and lipid/kg/day over 7 days.  The enteral feeding volume of milk was increased by 6–12 ml/d until the target volume of 165 ml/kg/d.  Formulation:  Enhanced parenteral and enteral nutrition; the intervention PN contained 20% more energy (117 kcal/kg/day) than the standard feeding regimen with proportional increase in dextrose (16.3 g/kg/day), protein (4 g/kg/day) and fat (4 g/ kg/day). The micronutrients within the two PN were the same and as recommended.  Expressed breast milk (EBM) fortified with Nutricia (Cow & Gate) breast milk fortifier or preterm formula Nutriprem (Cow & Gate) | Dose:  PN increased stepwise from 1 g protein and lipid/kg/day to 3 g protein and lipid/kg/day over 5 days.  The enteral feeding volume of milk was increased by 6–12 ml/d until the target volume of 165 ml/kg/d.  Formulation:  Standard parenteral and enteral nutrition;  The standard PN contained 93 kcal/kg/day, dextrose (13.5 g/kg/day), protein (3 g/kg/day) and fat (3 g/kg/day).  Expressed breast milk (EBM) fortified with Nutricia (Cow & Gate) breast milk fortifier or Osterprem (Farleys) | Duration:  28 days  Started:  Within 7 days of life  Ended:  At 34 weeks of post-menstrual age | Primary:  Occipitofrontal circumference at 36 weeks PMA  Cognition/ Behavior:  BSID-II at 3 and 9 months PMA; MDI and PDI  Neuroimaging:  Quantitative MRI at 40 weeks PMA;  Total Brain Volume (TBV), Cortical Brain Volume (CBV) and T2 relaxation time | MDI and PDI:  No significant differences between the groups. Results were similar in subgroup analysis by sex.  When data from both groups were pooled, energy and protein deficits at 28 days correlated significantly with MDI and PDI at 3 months post-term but the correlation was not significant at 9 months post-term.  MRI:  No significant differences in the occurrence of gross abnormalities, TBV, CBV and T2 relaxation times between the two groups. Subgroup analysis by sex did not reveal significant differences.  After adjusting for confounding factors a significant relationship between TBV and T2 relaxation time and MDI was found. |
| Blakstad et al 2015  Norway  RCT | Sample:  VLBW infants, free from major congenital abnormalities  Age & Weight:  Median values in intervention group: 903g and 27.7 weeks;  Median values in standard feeding group: 1130g and 28.7 weeks  Randomized:  50; 26 in intervention and 24 in standard feeding group  Followed up:  31/50 (62%) | Dose:  Parenteral nutrition (PN) started at 3.5 g/kg/day amino acids and 2.0 g/kg/day fat. The maximum parenteral fat supply was 3.4 g/kg/d over 7 days. The protein supply was gradually increased mostly by enhanced enteral nutrition.  Human milk was increased by 10-20 mL/kg/d until the target value of 170 mL/kg/d of fortified milk. In addition, infants received a supplement of amino acids (0.6 g/100 ml; Complete Amino Acid Mix®, Nutricia, Norway), DHA (60 mg/kg/d), ARA (60 mg/kg/d) and vitamin A (1500 mg/kg/d) .  In average, infants received per kg/day 92.4 Kcal, 3.7 g protein, 4.1 g fat, 23.1 mg ARA, 56.3 mg DHA, 379.6 microg vitamin A and 0.95 mmol phosphate during the first week. Other nutrient intake was not different between groups.  On full enteral feeding, infants received 166 kcal/Kg/d energy and 4.4 g/kg/d protein.  Formulation:  Parenteral fat emulsion (SMOF® lipid, Fresenius Kabi, Norway) providing DHA and ARA.  Enteral hHuman milk fortified with 4.2 g Nutriprem®/100 mL (Nutricia, Norway), 0.6 g/100 ml Complete Amino Acid Mix® (Nutricia, Norway), DHA and ARA (Martek Biosciences Corporation, USA) and vitamin A (As Produksjonslab,Norway) | Dose:  PN started at 2.0 g/kg/d amino acids and 0.5 g/kg/day fat. The maximum parenteral fat supply was 3.4 g/kg/d over 7 days. The protein supply was gradually increased mostly by enhanced enteral nutrition.  Human milk was increased by 10-20 mL/kg/d until the target value of 170 mL/kg/d of fortified milk.  In average, infants received per kg/day 78.6 Kcal, 2.5 g protein, 3.3 g fat, 11.3 mg ARA, 16.8 mg DHA, 153.2 microg vitamin A and 0.84 mmol phosphate during the first week. Other nutrient intake was not different between groups.  On full enteral feeding, infants received 146 kcal/Kg/d energy and 3.6 g/kg/d protein.  Formulation:  Standard parenteral fat emulsion (ClinOleic® (Baxter, Norway).  Enteral human milk fortified with 4.2 g Nutriprem®/100 mL (Nutricia, Norway) | Duration:  24 weeks  Started:  Within 24 h of life  Ended:  At 52 weeks of post-menstrual age and/or body weight of 5.5 kg | Primary:  Proportion of infants discharged as growth restricted  Electrophysiology:  VEP at 5 months of CA | VEP:  The motion f1 component was larger and more centrally focused in the intervention than in the control group. In addition, the intervention group had a significant f3 motion response, which was absent in the control group. |
| Strommen et al 2015  Norway  RCT | Sample:  VLBW infants, free from major congenital abnormalities  Age & Weight:  Median values in intervention group: 932g and 27 weeks;  Median values in standard feeding group: 1166g and 29 weeks  Randomized:  50; 26 in intervention and 24 in standard feeding group  Followed up:  25/50 (50%) | Dose:  Parenteral nutrition (PN) started at 3.5 g/kg/day amino acids (AA) and 2.0 g/kg/day fat. The maximum parenteral fat supply was 3.4 g/kg/d over 7 days. The protein supply was gradually increased mostly by enhanced enteral nutrition.  Human milk was increased by 10-20 mL/kg/d until the target value of 170 mL/kg/d of fortified milk. In addition, infants received a supplement of AA (0.6 g/100 ml; Complete Amino Acid Mix®, Nutricia, Norway), DHA (60 mg/kg/d), ARA (60 mg/kg/d) and vitamin A (1500 mg/kg/d).  In average, infants received per kg/day 92.4 Kcal, 3.7 g protein, 4.1 g fat, 23.1 mg ARA, 56.3 mg DHA, 379.6 microg vitamin A and 0.95 mmol phosphate during the first week. Other nutrient intake was not different between groups.  On full enteral feeding, infants received 166 kcal/Kg/d energy and 4.4 g/kg/d protein.  Formulation:  Parenteral fat emulsion (SMOF® lipid, Fresenius Kabi, Norway) providing DHA and ARA.  Enteral Human milk fortified with 4.2 g Nutriprem®/100 mL (Nutricia, Norway), 0.6 g/100 ml Complete Amino Acid Mix® (Nutricia, Norway), DHA and ARA (Martek Biosciences  Corporation, USA) and vitamin A (As Produksjonslab, Norway). | Dose:  PN started at 2.0 g/kg/d AA and 0.5 g/kg/day fat. The maximum parenteral fat supply was 3.4 g/kg/d over 7 days. The protein supply was gradually increased mostly by enhanced enteral nutrition.  Human milk was increased by 10-20 mL/kg/d until the target value of 170 mL/kg/d of fortified milk.  In average, infants received per kg/day 78.6 Kcal, 2.5 g protein, 3.3 g fat, 11.3 mg ARA, 16.8 mg DHA, 153.2 microg vitamin A and 0.84 mmol phosphate during the first week. Other nutrient intake was not different between groups.  On full enteral feeding, infants received 146 kcal/Kg/d energy and 3.6 g/kg/d protein.  Formulation:  Standard parenteral fat emulsion (ClinOleic® (Baxter, Norway).  Enteral human milk fortified with 4.2 g Nutriprem®/100 mL (Nutricia, Norway) | Duration:  24 weeks  Started:  Within 24 h of life  Ended:  At 52 weeks of PMA and/or body weight of 5.5 kg | Primary:  Proportion of infants discharged as growth restricted  Neuroimaging:  MRI (mean diffusivity (MD) in cerebral white matter) at around term-equivalent age  Other:  Head circumference | MRI:  When non-adjusted for confounders, MD was significantly lower in cingulum, corticospinal tract, superior longitudinal fasciculi and uncinated fasciculi in the intervention group. MD values in the intervention group remained significantly lower in the superior longitudinal fasciculi when adjusted for birth weight and age when scanned. All other MD values were marginally significantly lower in the intervention group when adjusted for birth weight, age when scanned and late-onset septicemia.  Head circumference:  Marginally significantly higher head circumference growth in intervention than in control group |
| Stephens et al 2009  USA  Cohort study | Sample:  ELBW infants  Age & Weight:  Mean cohort values: 787 ±133g and 25.9±1.6 weeks;  43% boys and 13% SGA infants; Supplemental oxygen requirement: on average 57±33 days;  Ventilator support: on average 19±17 days;  NICU stay: on average 89±27 days  Non-randomized study:  148 infants    Followed up:  124 (84%) with BSID at 18 mo | Dose:  Daily total enteral and parenteral intakes during the first 4 weeks of life. Mean daily protein and energy intakes were calculated for weeks 1, 2, 3, and 4 based on what the infants actually received according to the feeding data.  Formulation:  Parenteral nutrition provided 3.4 kcal/g carbohydrate in a monohydrate form, 4.0 kcal/g protein as Trophamine, and 2.0 kcal/ml lipid as Intralipid 20% emulsion. Protein was started in the ﬁrst bag of parenteral nutrition at 1.0 g/kg per day and advanced by 0.5 g/kg per day up to 2.5 to 3.5 g/kg per day.  Enteral feeds were breast milk (nutritional content presumed that of mature breast milk with 20.0 kcal/oz and 1.54 g of protein per 100 kcal) with or without Similac Human Milk Fortiﬁer (additional 14.0 calories and 1.0 g of protein per 100 ml) or preterm formula initiated at 20.0 kcal/oz and advanced to 24 –27 kcal/oz once full enteral feeds were tolerated. The formulations of the 2 preterm formulas used provided 2.7 and 3.0 g of protein per 100 kcal. | | Duration:  4 weeks  Start:  From birth | Primary:  None defined (no power calculation)  Cognition/ Behavior:  BSID-II (MDI and PDI) at 18 months CA | BSID-II  The mean energy intake and the mean protein intake during the ﬁrst week of life contributed signiﬁcantly and independently to the MDI at 18 months.  During the first week of life each 10 kcal/kg/d increase in energy intake was associated with a 4.6-point increase in MDI, and each 1-g/kg/d increase in protein intake was associated with an 8.2-point increase in MDI at 18 months. |
| dit Trolli et al 2012  France  Cohort study | Sample:  VLBW infants, GA<28 weeks in NICU, free from malformations and metabolic disease  Age & Weight:  Mean values of the cohort: 928±197g and 26.8±1 weeks | Dose:  Cumulative intake of energy and macronutrients at days 7, 14 and 28  Formulation:  PN  *Proteins* as amino acid solutions (Vaminolact®, Fresenius Kabi France, Sèvres, France or Primène↓, Baxter, Deerﬁeld, USA) at a dose of 1 g/kg/d from the ﬁrst day of life, then increased by 0.5 to 1 g/kg/d to reach 4 g/kg/d;  *Carbohydrates* from birth at a dose of 6 to 8 g/kg/d and increased by 1 to 2 g/kg/d depending on the carbohydrate tolerance to 14 to 17 g/kg/d;  *Lipids* initiated in the form of a 20% lipid emulsion (Medialipides 20%↓, Braun Medical, Melsungen, Germany) during the ﬁrst three days of life and increased stepwise by 0.5 to 1 g/kg/d to reach 4 g/kg/d.  Starting volume 70 to 80 ml/kg/d on the ﬁrst day, increasing by 10 to 20 ml/kg /d up to a maximum of 160 ml/kg/d.  EN  *Pasteurized human milk* at a dose of 10 ml/kg/d, increasing by 10 to 15 ml/kg/d to reach 160 ml/kg/d. The transfer to maternal milk asap. | | Duration:  28 days  Started:  Immediately after birth  Ended:  28 days after birth | Primary:  None defined (no power calculation)  Cognition/ Behavior:  Brunet-Lezine (BL) at 28 days of life and at 12 months CA; Global score (= develop-mental quotient; DQ), sub-score for posture and motricity, coordination, language and socialization | BL:  Simple linear regression analyses showed a signiﬁcant correlation between the DQ and the cumulative intakes of energy and lipids at 14 days of life (coefﬁcient: 0.02, P=0.02, r² = 0.11; coefﬁcient: 0.27, P=0.01, r² = 0.13, respectively).  No signiﬁcant correlation between the DQ and the cumulative intake of carbohydrates or proteins at 7, 14 or 28 days of life.  In a multivariate analysis, the association between the cumulative intake of lipids at 14 days of life and the DQ remained statistically signiﬁcant (coefﬁcient: 0.26, P=0.04, r² = 0.42). |

TABLE S2. BREAST MILK STUDIES

| Author, year, country, design | Study population | Intervention diet  (dose and formulation) | Control diet  (dose and formulation) | Intervention/ Observation period | Outcome measures | Effect of the intervention/  Findings of cohort study | |
| --- | --- | --- | --- | --- | --- | --- | --- |
| Fortified maternal breast milk vs unfortified maternal breast milk | | | | | | | |
| Lucas et al 1996  UK  Follow-up of a RCT | Sample:  Preterm infants, BW<1850 g, GA<37 weeks, in NICU, free from major congenital malformations; mothers providing breast milk  Age & Weight:  Mean values in the fortified mother’s milk group: 1306 ±28 g and 29.8±0.2 weeks;  Mean values in the unfortified mother’s milk group: 1263±26 g and 29.8±0.2 weeks  Randomized:  275; 137 in the fortified mother’s milk and 138 in the unfortified mother’s milk (control) group  Followed up:  100% | Dose:  Fortified human milk, full enteral feed 180ml/kg/d  Formulation:  Human milk fortified with a multinutrient fortifier (Enfamil human milk fortifier; Mead Johnson, Evansville, IN) and vitamins.  A preterm formula (Osterprem; Farley Health Products Ltd, Kendal, United Kingdom) was used to bring total feed volume to the prescribed amount, reaching 180 ml/kg/d when full enteral feeds were established.  Non-nutritional:  Advice in breastfeeding | Dose:  Human milk, full enteral feed 180 ml/kg/d  Formulation:  Human milk fortified with a control supplement containing only sodium and potassium phosphate and vitamins.  A preterm formula (Osterprem; Farley Health Products Ltd, Kendal, United Kingdom) was used to bring total feed volume to the prescribed amount, reaching 180 ml/kg/d when full enteral feeds were established. | Duration:  Mean 39 days  Started:  Mean 10 days after birth  Ended:  Body weight >2000 g or discharge from the hospital | Primary:  None defined (no power calculation)  Cognition/ Behavior:  Knobloch et al Developmental Screening Inventory at 9 months CA;  BSID (MDI and PDI) at 18 months CA;  Vineland Social Maturity Scale at 18 months CA | Knobloch et al.  No significant difference between groups.  MDI and PDI and Vineland Social Maturity Scale  The test scores were non-significantly higher in the fortified group by 2.2 points for the MDI, by 2.4 points for the PDI, and by 3. 1 points for social maturity.  The study results failed to demonstrate beneficial effects of breast milk fortifiers on mental development, psychomotor development or social maturity scores either at 9 months or 18 months CA. | |
| Lucas et al 1998  UK  RCT | Sample:  Preterm infants, BW<1850 g, in NICU, free from major congenital malformations.  Age & Weight:  Mean values in different trial groups were not significantly different and varied from 1354±66 g and 30.6±0.3 days to 1425±28 g and 31.3±0.3 days  Randomized:  424; 160 in trial A, 264 in trial B;  Followed up:  360/377 of survivors (96%)  47 died; 19 in trial A, 28 in trial B | Dose:  Preterm formula according to requirement  Formulation:  A preterm formula (Osterprem, Farley Health products Ltd) containing 2.0g protein and 80 kcal/100 ml and enriched with sodium, calcium, phosphorus, copper, zinc, vitamins D; E and K, water soluble vitamins, carnitine and taurine.  Trial A as sole diet;  Trial B as a supplement to mother’s breast milk | Dose:  Standard term formula according to requirement  Formulation:  A standard term formula (Osterfeed = Ostermilk, Farley Health Products Ltd) containing 1.45 g protein and 68 kcal/100 ml;  Trial A as sole diet;  Trial B as a supplement to mother’s breast milk | Duration:  Around 30 days  (Median 26 to 33 days)  Started:  From birth; enteral feeds were increased according to tolerance to aim 180 ml/kg/d (median period 9 days).  Ended:  When the infant reached the weight of 2000 g or was discharged from NICU. | Primary:  WISC-R UK at 7.5-8 CA  Cognition/ Behavior:  WISC-R UK (VIQ, PIQ and FSIQ) | WISC-R UK  VIQ was 4.8 points higher (P=0.8) in those fed solely a preterm formula (trial A) versus standard term formula. No significant (P>0.05) differences in VIQ, PIQ and FSIQ between groups in trial A or B or in both trials combined.  A major sex difference in the impact of diet was reported. The beneficial effect of preterm formula on VIQ was seen for boys but not girls and was confined to those fed exclusively on the trial diets (trial A). Boys on the preterm formula had a 12.2 (95% CI, 3.7 to 20.6) point advantage in VIQ and a 6.3 (95% CI,− 1.5 to 14.2) point advantage in FSIQ, whereas no effect was seen in girls.  Advantages in VIQ and FSIQ were even larger among boys who received the highest intake of trial diet (trial A and trial A plus B combined). The advantage in verbal IQ was 14.4 (95% CI, 5.7 to 23.2) and in overall IQ was 9.5 (95% CI, 1.2 to 17.7) points for boys with the highest intakes of preterm formula. | |
| Fortified maternal breast milk vs nutrient enriched formula | | | | | | | |
| Bier et al 2002  USA  Cohort study | Sample:  LBW infants, BW< 2000g, no maternal illicit drug use, mental illness or HIV infection  Age & Weight:  Mean values in breast milk group: 1174±305 g and 28.6±2.9 weeks;  Mean values in formula group: 1198±170 g and 29.9±2.2 weeks  Non-randomized study:  29 in breast milk group, 10 in formula group | Dose:  During the stay in special care nursery the mean intake of breast milk was 852±429 ml/wk by gavage or bottle and the mean intake of premature infant formula 170±258 ml/wk  Formulation:  Breast milk with HMF (one 4-kcal packet/25ml breast milk)  >80% of intake was maternal breast milk  Non-nutritional:  Help with breast and bottle feeding | Dose:  Not mentioned  Formulation:  Premature infant formula, containing 24kcal/fl oz, iron fortified and 11% whey. From 32 weeks gestation, formula was iron-fortified and 22kcal/fl oz. | Duration:  Mean time of maternal breast milk = 24±20 weeks  Started:  In the first two weeks of life  Ended:  12 months of CA (the time the mothers kept the feeding diary) | Primary:  None defined (no power calculation)  Cognition/ Behavior:  AIMS at 3,7,12 months CA  MDI of the BSID at 7 and 12 months CA | AIMS  The breast milk group had a significantly higher mean score compared to the formula group at 3 months and 12 months CA; 48 ± 20% vs 35 ± 12% (P = .05) and 63 ± 20% vs 46 ± 15% (P < .05), respectively.  BSID (MDI)  At 12 months of CA, the breast milk group had a higher mean score (101 ± 11 vs 90 ± 9; P < .05) compared to the formula group. At 7 months CA the difference was not significant. | |
| O’Connor et al 2003  Chile, UK & USA  Cohort study (retrospect.) | Sample:  LBW infants, in NICU, BW between 750-1800g, GA< 33 weeks; free from congenital abnormalities  Age & Weight:  Mean values for 4 different groups varied from 1275±312 g to 1332±279 g and from 29.5±2.1 weeks to 29.9±2.0 weeks  Non-randomized study:  470 infants  Followed up:  463 infants (99%);  43 in PHM-T;  98 in >50% HM;  203 in <50%HM;  119 in PFF-T | PHM-T = predominantly human milk fed until term;  PFF-T = predominantly formula fed until term  Dose:  1. PHM-T: consumed <100 ml/kg of formula during their hospital stay and >80% as human milk at term CA  2. >50% HM: >50% of total energy as human milk  3. <50% HM: <50% of total energy as human milk  Formulation:  Human milk, which was fortified from birth until discharge with a powdered or liquid fortifier to contain 22-24 kcal/oz and to deliver a minimum of 2.8 g protein/kg/d  When the infants were weaned from breast milk they carried on with the post-discharged nutrient enriched formula  (modified NeoSurc® powder, 73 kcal/100 ml, Ross Product Division, Columbus, Ohio, USA) until 12 mo CA | Dose:  PFF-T: <100ml/kg human milk during their hospital stay and >80% as formula at term CA  Formulation:  Specially designed in-hospital preterm formula (modified Similac Special Care^®^ ready-to-feed (79 kcal/100 ml), Ross Products Division, Columbus, Ohio, USA) with or without ARA and DHA-enriched oils.  Post-discharge nutrient enriched formula (modified NeoSurc® powder, 73 kcal/100 ml, Ross Product Division, Columbus, Ohio, USA) until 12 mo of CA | Duration:  Mean hospital stay = 37±23 days to 45±25 days for different groups  Started:  From first enteral feeding, before the 28^th^ day of life  Ended:  At term CA | Primary:  None defined (no power calculation)  Cognition/ Behavior:  Teller Acuity Card Procedure at 2, 4 and 6 months CA  BSID-II (MDI and PDI) at 12 months of CA;  MCDI at 14 months of CA | Teller Acuity Card Procedure  There was a small but statistically significant effect of human milk feeding.  BSID-II  No group difference in MDI or PDI.  MCDI  No group difference at 14 months CA. | |
| Furman et al 2004  USA  Cohort study | Sample:  VLBW infants, in NICU,  singleton birth, BW 600-1499 g, GA<33 weeks, absence of positive drug screen, major congenital anomaly, and intrauterine infection  Age & Weight:  Mean BW of the cohort: 1056 g;  Mean GA of the cohort: 28 weeks  Non-randomized study:  119 VLBW infants  Followed up:  98/119 (82%);  69/98 received fortified maternal milk and preterm formula supplementation, 29 received preterm formula only.  Mean BW of the followed infants 1012± 244 g, mean GA 27±2 weeks | Dose:  44±32ml/kg/d maternal milk  Formulation:  Maternal milk was fortified by Enfamil Human Milk Fortifier (Mead Johnson Nutritionals, Evansville, IN) and preterm infant formula Similac  Natural Care^ ^®(Ross Products Division, Abbott Laboratories, Columbus, OH) offered when the infant reached an oral intake of 110 mL/kg/day.  Mean intake of maternal milk was 40% of total intake  Non-nutritional:  Strong encouragement to provide breast milk | Dose:  110ml/kg/d preterm formula?  Formulation:  Preterm infant formula, Similac  Natural Care (Ross Products Division, Abbott Laboratories, Columbus, OH) | Duration:  4 weeks  Started:  At day 2 or 3 of life  Ended:  4 weeks later | Primary:  None defined (no power calculation)  Cognition/ Behavior:  BSID-II (MDI and PDI) and  Amiel-Tison Neurological Assessment (CP and NDI) at 20 months CA | MDI, PDI, CP and NDI  No significant difference between maternal milk intake and preterm formula groups for any of the outcomes measured after multivariate analysis adjusting for neonatal risk and social risk. | |
| Vohr et al 2006  USA  Cohort study & follow-up of a clinical trial | Sample:  ELBW infants, in NICU  Age & Weight:  Mean values in breast milk group: 785±129 g and 26.5±2 weeks;  Mean values in non-breast milk group: 794±133 g and 26.7±2 weeks.  Non-randomized study:  1433 infants  Followed up:  1035 of 1159 surviving infants (89%); 775 in breast milk group and 260 in non-breast milk group | Dose:  Breast milk (BM), full enteral feeds achieved at 29.0±18 days, with energy intakes of 107.5kcal/d  Formulation:  Fresh BM supplemented with breast milk fortifier and preterm formula  (Quintiles of breast milk intake: <20^th^, 20^th^-40^th^, 40^th^-60^th^, 60^th^-80^th^ and >80^th^; the total amount of BM for every day of hospitalization per quintiles were 1.0, 7.3, 24.0, 63.8 and 110.6 ml/kg/d, respectively). | Dose:  Non-breast milk, full enteral feeds achieved at 27.4±15 days with energy intakes of 105.9 kcal/d  Formulation:  High-nutrient preterm formula | Duration:  Approx. 110 days  Started:  Asap after birth; in breast milk group at mean age of 9.3±9 days and in non-breast milk group at mean age of 7.3 days  Ended:  At discharge or 120 days | Primary (original clinical trial):  Risk of mortality  Cognition/ Behavior:  BSID-II (MDI, PDI, BRS) and  Amiel-Tison Neurological Assessment at 18 to 22 months CA | MDI, PDI, BRS  Children in the breast milk group were significantly more likely to have a MDI>85, a higher mean PDI, and higher BRS percentile scores for orientation/engagement, motor regulation, and total score.  Multiple-regression analyses adjusted for confounders showed a signiﬁcant independent association of breast milk and all outcomes.  For every 10 ml/kg/day increase in breast milk ingestion the MDI increased by 0.53 points, the PDI increased by 0.63 points, and the BRS percentile score increased by 0.82 points.  Amiel-Tison Neurological Assessment  No differences in the rates of moderate to severe NDI between the 2 study groups | |
| Vohr et al 2007  USA  Cohort study & follow-up of a clinical trial | Sample:  ELBW infants, in NICU  Age & Weight:  Mean values in breast milk group: 785±129 g and 26.5±2 weeks;  Mean values in non-breast milk group: 794±133 g and 26.7±2 weeks.  *Comment: 3 sites including 279 infants were not included in the follow-up assessment.*  Non-randomized study:  1433 infants  Followed up:  773/939 surviving infants (82%); 593 in breast milk, 180 in non-breast milk group;  Breast milk group quintiles according to breast milk ingested;  <20^th^: <23.1 ml/kg/d;  20^th^-40^th^: 23.1-53 ml/kg/d;  40^th^-60^th^: 53-83.2 ml/kg/d;  60^th^-80^th^: 83.2-112.5 ml/kg/d;  >80^th^: >112.5 ml/kg/d | Dose:  In the highest breast milk, quintile full enteral feeds were achieved at mean 21.2 days, with energy intakes of 110 kcal/d  Formulation:  Fresh breast milk supplemented with breast milk fortifier and preterm formula | Dose:  Non-breast milk, full enteral feeds were achieved at mean 28.4 days with energy intakes of 110 kcal/d  Formulation:  High-nutrient preterm formula | Duration:  Mean = 85 days (breast milk group); mean = 97.6 days (non-breast milk group)  Started:  Asap after birth; in the highest breast milk quintile at mean age of 6.1 days and in non-breast milk group at mean age of 7.2 days  Ended:  At discharge or 120 days | Primary:  Risk of mortality  Cognition/ Behavior:  BSID-II (MDI, PDI, BRS) and  Amiel-Tison Neurological Assessment at 30 months CA | BSID-II (MDI and PDI)  The scores in the 3 highest quintiles of breast milk intake were signiﬁcantly higher (P< 0.05) than in the non-breast milk group.  BSID-II (BRS)  The emotional regulation score was signiﬁcantly higher (P<0.05) in 2 of 3 highest quintiles. Motor quality was signiﬁcantly higher (P<0.05) in the 2 highest quintiles, and the total behavior score was signiﬁcantly higher or trended higher than the no-BM group in the 3 highest BM intake quintiles. |  |
| Gibertoni et al 2015  Italy  Cohort study | Sample:  VLBW infants (<1500 g and/or ≤32 weeks GA) without severe congenital malformations, in NICU  Age & Weight:  1149±341 g and 29.0±2.3 weeks  Non-randomized study:  316 infants  Followed up:  87% (276/316); 109 in breast milk, 114 in mixed milk, 93 in formula milk  *Comments: group size information is given in reference to the initial cohort. No group size information is provided for the studied population. Information on feeding regime was collected at discharge and assumed to mimic the regime used along infant hospitalization* | Dose:  Breast milk group: 100% feedings as breast-milk  Mixed milk: >50% feedings as breast milk),  Formulation:  Fresh breast milk supplemented with breast milk fortifier when daily enteral intake reached 100 ml/Kg  Breast milk fortification was recommended after discharge until 3.5 Kg body weight | Dose:  Formula milk: 100% feedings as preterm formula  Formulation:  Preterm formula, containing 80–90 kcal/100 ml and 2–2.3 g protein/100 ml  Post-discharge formula was recommended after discharge until 3.5 Kg body weight | Duration:  Not reported  Started:  Not reported  Ended:  Not reported | Primary:  None defined (no power calculation)  Cognition/ Behavior:  Revised GMDS at 2 years CA | GMDS  Both breast and mixed milk groups had higher General Quotient scores than formula milk group at 2 years CA.  Breast milk consumption was significantly associated with higher General Quotient scores in the univariate analysis and also after adjusting for potential confounders. |  |
| Unfortified maternal breast milk vs infant formula | | | | | | |  |
| Eidelman & Feldman 2004  Israel  Cohort study | Sample:  LBW infants, BW<1750 g, GA<33 weeks, free from grade 3 and 4 IVH, perinatal asphyxia, metabolic or genetic disease  Age & Weight:  Mean values: 1298 ±336 g and 30.5±3 weeks  Non-randomized study:  86 infants; 34 in the highest breast milk group, 21 in the medium and 31 in the minimal breast milk group  Followed up:  All the infants are included in the outcome results | Dose:  1. >75% breast milk  2. 25-75% breast milk  Formulation:  Breast milk and formula;  Feeding from breast <5% | Dose:  <25% breast milk  Formulation:  Breast milk and formula | Duration:  4 weeks  Started:  Asap after birth  Ended:  At discharge, 37 weeks of gestation | Primary:  None defined (no power calculation)  Cognition/ Behavior  BNBAS (habituation, motor maturity, range of states) at 37 weeks of PMA  BSID (MDI and PDI) at 6 months CA | BNBAS  Significant group differences in motor maturity and range of states but not in habituation. Dose response pattern for motor maturity.  BSID (MDI and PDI)  Significant differences between the highest breast milk group and the other two groups. |  |
| Morley et al 1988  UK  Epidemio-logical comparison study  (Partly the same study population as in Lucas et al 1992) | Sample:  Preterm infants, BW<1850g, in NICU, free from major congenital malformations  Age & Weight:  Mean values not reported  Non-randomized study:  834 infants  Followed up:  89% for BSID at 18 mo; 91% for Academic Scale of Developmental Profile at 18 mo | Dose:  According to the requirement  Formulation:  Maternal breast milk, supplemented with preterm (Osterprem, Farley Health Products Ltd) or term formula  (Osterfeed = Ostermilk, Farley Health Products Ltd) | Dose:  According to the requirement  Formulation:  Preterm (Osterprem, Farley Health Products Ltd) or term formula (Osterfeed = Ostermilk, Farley Health Products Ltd) | Duration:  4 weeks  Started:  Immediately after birth  Ended:  When the infant reached the weight of 2000 g or was discharged from NICU | Primary:  None defined (no power calculation)  Cognition/ Behavior  BSID (MDI) at 18 months CA  The Academic Scale of Developmental Profile II (IQ equivalent) at 18 months CA | MDI and IQ  Both MDI and IQ equivalent were significantly higher for the group of infants whose mothers chose to provide breast milk, with an advantage of 8 points in MDI and 7 points in IQ equivalent.  After adjusting for confounding variables a significant advantage of 4.3 points (P<0.01) remained in MDI and IQ equivalent for babies whose mothers chose to provide breast milk. |  |
| Lucas et al 1992  UK  Epidemio-logical comparison study  (same study population as in Morley et al 1988) | Sample:  Preterm infants, BW<1850 g, in NICU, free from major congenital malformations  Age & Weight:  Mean values in mother’s milk group: 1440±20 g and 31.4±0.2 weeks;  Mean values in no mother’s milk group: 1420±30g and 31.4±0.3 weeks  Non-randomized:  313 surviving children were invited to be tested at the age of 7.5-8 years of CA  Followed up:  300 children tested (96%); 210 in mother’s milk group, 90 in no mother’s milk group | Dose:  According to the requirement  Formulation:  Maternal breast milk, supplemented with preterm (Osterprem, Farley Health Products Ltd) or term formula  (Osterfeed = Ostermilk, Farley Health Products Ltd) or pasteurised donor breast milk  Proportions of preterm formula, pasteurized donor breast milk and term formula were 50% (n=105), 31% (65) and 19% (40). | Dose:  According to the requirement  Formulation:  Preterm (Osterprem, Farley Health Products Ltd) or term formula (Osterfeed = Ostermilk, Farley Health Products Ltd) or pasteurised donor breast milk  Proportions of preterm formula, pasteurized donor breast milk and term formula were 51% (n=46), 31% (28) and 18% (16). | Duration:  Mean = 28 days (mother’s milk group);  Mean = 30 days (for no mother’s milk group)  Started:  Immediately after birth  Ended:  When the infant reached the weight of 2000 g or was discharged from NICU | Primary:  None defined (no power calculation)  Cognition/ Behavior:  WISC-R (VIQ, PIQ, FSIQ) at 7.5-8 years CA | WISC-R  Children in mother’s milk group had significantly higher VIQ, PIQ, FSIQ scores compared to children who did not receive mother’s milk. The difference remained after adjusting for confounding factors, with an advantage of around 8 points in the mother’s milk group. |  |
| Tanaka et al 2009  Japan  Follow up study of a clinical trial | Sample:  Preterm infants  Age & Weight:  Mean values breast milk group: 1016.4 ±302.2 g, 28.7±3.2 weeks;  Mean values formula group: 1188.0±296.3 g, 30.7±1.6 weeks  Randomized:  38 infants enrolled  Followed up:  10 in breast milk group, 8 in formula group (18/28= 69%) | Dose:  Breast milk > 80% of total feed  Formulation:  Mother’s breast milk and formula milk  RBC membrane DHA level at 4 weeks of age as a measure of intake of breast milk | Dose:  Formula milk, the proportion of breast milk< 80% of total feed  Formulation:  Formula milk and mother’s breast milk  RBC membrane DHA level at 4 weeks of age as a measure of intake of breast milk | Duration:  4 weeks  Started:  Immediately after birth  Ended:  After 4 weeks | Primary:  None defined (no power calculation)  Cognition/ Behavior  1.KABC  2.Day–Night Test  3.KRISP  4.Motor Planning Test  5.SDQ  At 5 years of age  Biological samples  DHA concentration of RBC membrane at 4 weeks of age | KABC  Mean values for sequential processing were significantly (P<0.05) higher in breast milk vs formula group; no group differences in simultaneous processing and mental processing  Day–Night Test, KRISP, Motor Planning Test  Mean values were significantly (P<0.05) higher in the breast milk than in the formula group.  SDQ  Mean values for hyperactivity, emotional, total scores were significantly (P=0.05) lower (=better) in the breast milk than in the formula group. |  |
| Pinelli et al 2003  Canada  Follow up study of a clinical trial | Sample:  VLBW infants, BW<1500 g  Age & Weight:  Mean values breast milk group: 1130 ±244 g and 29 ±3 weeks;  Mean values formula group: 1090±273 g and 29±3 weeks  Non-Randomized:  148 infants  Followed up:  117 in breast milk group, 20 in formula group (93%) | Dose:  Breast milk feeding >80%  Formulation:  Only 50% (n = 64) received BM exclusively in the NICU. Others received preterm formula supplements in varying amounts (SMA Preemie 24,Wyeth Inc, Markham, Ontario, Canada), vitamins (Polyvisol; Mead Johnson, Ottawa, Ontario, Canada) or mineral supplements (calcium and phosphorus supplement prepared in hospital pharmacy), and human milk fortification (Enfamil Human Milk Fortifier; Mead Johnson, Evansville, IN) during their time in the NICU. | Dose:  Preterm formula (breast milk feeding<80%)  Formulation:  In addition to breast milk preterm formula supplements in varying amounts (SMA Preemie 24,Wyeth Inc, Markham, Ontario, Canada), vitamins (Polyvisol; Mead Johnson, Ottawa, Ontario, Canada) or mineral supplements (calcium and phosphorus supplement prepared in hospital pharmacy), and human milk fortification (Enfamil Human Milk Fortifier; Mead Johnson, Evansville, IN) during their time in the NICU. | Duration:  Not reported  Started:  Immediately after birth  Ended:  At term 46% mothers were providing >80% breast milk, at 1 month of CA 40%, at 3 mo 30%, at 6 mo 15% and at 12 mo 4%. | Primary (original trial):  Success rate of breast feeding at 3 months CA  Cognition/ Behavior  BSID-II (MDI, PDI);  ITQ-R;  at 6 and 12 months CA | MDI and PDI  No differences in outcomes between diet groups.  ITQ-R  No significant differences between diet groups in the distribution of infants within the 5 temperament categories. |  |
| Banked breast milk vs preterm formula | | | | | | |  |
| Lucas et al 1989  UK  RCT | Sample:  Preterm infants, BW<1850 g, in NICU, free from major congenital malformations  Age &Weight:  Mean values in different trial groups were not significantly different and varied from 1364±300 g and 30.8±2.8 days to 1392±298 g and 31.2±2.8 days.  Randomized:  502; 83 sole banked milk, 76 sole preterm formula, 170 banked breast milk + mother’s milk, and 173 preterm formula + mother’s milk.  Followed up: From 84% to 89% between groups. | Dose: Preterm formula containing 2 g protein, 80 kcal, 35 mg phosphorus, 70 mg calcium, and 45 mg sodium per 100 ml.  Formulation:  Preterm formula as sole diet or in combination with mother’s own milk. The proportion of the supplement in the combination diet was 43.8% | Dose:  Banked donor breast milk  Formulation:  Banked donor breast milk  as sole diet or in combination with mother’s own milk.  The proportion of the banked milk in the combination diet was 51.3% | Duration:  Around 30 days;  Median days = 31 days (formula group); Median days = 28days (donor milk)  Started:  Immediately after birth when enteral feed was possible.  The median time for full enteral feeds between groups was 6-9 days.  Ended:  At the time of discharge from NICU or at a weight of 2000 g | Primary  None defined (no power calculation)  Cognition/ Behavior:  Knobloch et al Developmental Screening Inventory (5 scales of behavior: adaptive, gross motor, fine motor, language, and personal-social) at 9 months CA  Amiel-Tison Neurological Assessment at 9 months CA | Knobloch et al.  When the results of two trials were combined the infants receiving preterm formula, either as sole diet or as supplement to their mothers' milk, had higher mean scores in all areas of development than those given donor milk as sole diet or supplement.  Significant differences were seen in adaptive, fine motor, language, and personal-social areas, and in overall developmental quotient (95% CI for difference: 0.4 to 4.6, P<0.02).  Amiel-Tison  5% of the infants in the banked breast milk and 9% of the infants in the preterm formula group were neurologically impaired at 9 months CA. |  |
| Lucas et al 1994  UK  RCT | Sample:  Preterm infants, BW<1850 g, in NICU, free from major congenital malformations  Age & Weight:  Mean values in different trial groups were not significantly different and varied from 1385±26 g and 31.0± 0.4 days to 1414±37g and 31.5±0.4 days  Randomized:  502; 83 sole banked milk, 76 sole preterm formula, 170 banked breast milk + mother’s milk, and 173 preterm formula + mother’s milk.  Followed up: 96% (438/457 survivors). | Dose:  The preterm formula containing 2.0 g protein and 80 kcal/100 ml and enriched in sodium, calcium, phosphorus, copper, zinc, vitamins D, E, K, water-soluble vitamins, camitine, and taurine.  Formulation:  The preterm formula (Farley's Osterprem; Crookes Health Care) as sole diet (trial A) or as a supplement with mother’s milk (trial B).  The median intake of maternal milk in trial B was 55%. | Dose:  Banked breast milk with mean protein content of 1.1 g/100 ml and a mean energy content <50 kcal/100 ml.  Formulation:  Pasteurised banked breast milk as sole diet (trial A) or as a supplement with mother’s own milk (trial B).  The median intake of maternal milk in trial B was 50%. | Duration:  Around 30 days;  Median = 27 days (preterm formula group); Median = 31 days (banked breast milk group)    Started:  Immediately after birth when enteral feed was possible.  The median time to full enteral feeds in the whole study population was 7 days.  Ended:  At the time of discharge from NICU or at a weight of 2000 g | Primary:  BSID (MDI and PDI) at 18 months of CA | BSID  No differences in outcome measures between the two diet groups, but a low nutrient content of donor milk in relation to the preterm formula and to the estimated needs of preterm infants was reported. |  |
| Tyson et al 1983  USA  RCT | Sample:  Healthy LBW infants whose mothers were not breastfeeding by day 10 of life, BW<1500 g  Age & Weight:  Mean values in banked human milk: 1238 ±190 g, 29.4±3.1 weeks;  Mean values in preterm formula group: 1226±197 g, 29.4±2.4 weeks  Randomized:  76; 34 in BHM group and 42 in preterm formula group  Followed up:  100% | Dose:  According to the appetite of the infant.  The mean intake of preterm formula 165 ml/kg/d containing 87 kcal, 2.22 g protein, 4.04 g fat and 8.84 g carbohydrates 100 ml.  Formulation:  Similac Special Care formula by Ross Laboratories.  Plus daily 0.4 ml Aquasol, supplying 20 IU vitamin E, and 1.0 ml multivitamin preparation (Poly- Vi-Sol, Mead Johnson). | Dose:  According to the appetite of the infant.  Mean intake of BHM 197 ml/kg/d containing 60 kcal, 1.09 g protein, 2.21 g fat and 7.72 g carbohydrates per 100 ml.  Formulation:  Frozen banked human milk by unpaid volunteers.  Plus daily 0.4 ml Aquasol, supplying 20 IU vitamin E, and 1.0 ml multivitamin preparation (Poly- Vi-Sol, Mead Johnson). | Duration:  Approx. 20 days  Started:  10^th^ day after birth;  During the first 9 days after birth infants were routinely fed Similac PM 60/40 (Ross Laboratories) or their own mother’s milk.  Ended:  When the weight of 2000 g was reached or 30 days after birth;  Standard 20 kcal/oz formula feedings were initiated after the intervention time was up. | Primary:  Physical growth: length and weight at 37 weeks PMA  Cognition/ Behavior:  BNBAS at 37 weeks PMA | BNBAS  The mean score for the orientation scales that measure alertness and responsiveness to auditory and visual stimuli was 3.4±1.4 for the formula group and 2.6±1.0 for the BHM group (P<0.10).  The two groups differed significantly with respect to the orientation items assessing response to inanimate objects (e.g., bell and ball).  The mean total score for the inanimate stimuli was 7.5±3.0 for the formula group and 5.0±2.1 for the BHM group (P<0.02). |  |

| Duration of breast milk feeding and cognitive ability | | | | | | |
| --- | --- | --- | --- | --- | --- | --- |
| Horwood et al 2001  New Zealand  Cohort study (retrospect.) | Sample:  VLBW infants, in NICU  Age & Weight:  Not reported  Non-randomized study:  413 infants  Followed up:  326 (79%) infants survived until age of 7;  298 (91%) of surviving children were assessed at 7 yrs;  The analysis is based on data from 280 (86%) children who completed the assessment. | Duration of breast milk given;  1. < 4 months (99 infants)  2. 4-7 months (46 infants)  3. > 8 months (59 infants)  Mothers were asked retrospectively to report whether they had provided breast milk at birth, and for how long the infant had been breastfed. Mothers’ recall was crosschecked against the records of infant feeding practice. | No breast milk given (76 infants) | Duration:  Varied according to the time breast milk given  Started:  From birth  Ended:  At the time when breast milk was stopped | Primary  None defined (no power calculation)  Cognition/ Behavior:  WISC-R (VIQ and PIQ) at 7 yrs of age | WISC-R  After adjusting for social and perinatal factors there was a signiﬁcant association between duration of receipt of breast milk and VIQ (β=0.12, P<0.05), with a 6-point advantage for infants who received breast milk for min. eight months compared with those who did not receive breast milk.  There was a trend for a higher PIQ with longer duration of receipt of breast milk but this did not reach signiﬁcance (β= 0.08, P>0.15). |

TABLE S3. INFANT FORMULA STUDIES

| Author, year, country, design | Study population | Intervention diet  (dose and formulation) | Control diet  (dose and formulation) | Intervention/ Observation period | Outcome measures | Effect of the intervention/  Findings of cohort study |
| --- | --- | --- | --- | --- | --- | --- |
| Isaacs et al 2009  UK  RCT  (Follow-up of a subgroup from Lucas et al 1998) | Sample:  LBW infants, GA<30 weeks, neurologically normal at 8 yrs, with neuroimaging performed at 16 yrs  Age & Weight:  Mean values in high-nutrient group: 1250±283 g, 28.7±1.1 weeks;  Mean values in standard nutrient group: 1140±234 g, 28.3±1.3 weeks  Randomized:  236 infants; 123 males, 113 females  Followed up:  IQ data at 8 and 16 yrs from 95 infants; 95/236 (40%);  51 males,44 females;  46 in standard nutrient diet, 49 in high-nutrient diet group | Dose:  As required  Formulation:  A preterm formula (Osterprem, Farley Health Products Ltd); 2.0g protein and 80kcal/100ml, enriched with sodium, calcium, phosphorus, copper, zinc, vit D, E and K, water soluble vit, carnitine and taurine.  As a sole diet or as a supplement  to mother’s breast milk  2.5 g protein per 100 kcal | Dose:  As required  Formulation:  A standard term formula (Osterfeed = Ostermilk, Farley Health Products Ltd); 1.45 g protein and 68 kcal per 100 ml. As sole diet or as a supplement to mother’s breast milk. High-nutrient group received 40% more protein and 20% more energy than standard nutrient group.  2.1 g protein per 100 kcal | Duration:  Mean = 6 weeks  Started:  From birth; enteral feeds were increased according to tolerance to aim 180 ml/kg/d.  Ended:  When the infant reached the weight of 2000 g or was discharged from NICU. | Primary  None defined (no power calculation)  Cognition/ Behavior:  WISC-III, WAIS-III (VIQ, PIQ, FSIQ) at 8 and 16 yrs  Neuroimaging:  The results are reported in the study by Isaacs et al 2008 | VIQ, PIQ and FSIQ  At 8 yrs, the high-nutrient group had signiﬁcantly higher scores in all 3 IQ measures compared with the standard-nutrient group. At 16 y, the signiﬁcant effects of diet on VIQ persisted (101.0±2.0 vs. 94.8±2.0, P=0.03), with a trend-level effect on FSIQ but no longer for PIQ in the high-nutrient group.  Both diet groups scored signiﬁcantly lower (P<.001) on all 3 IQ measures at 16 yrs compared with 8 yrs. |
| Isaacs et al 2008  UK  Explanatory RCT  (Follow-up of a subgroup from Lucas et al 1998) | Sample:  LBW infants, GA<30 weeks, neurologically normal at 8 yrs  Age & Weight:  Mean values in high nutrient group: 1276±270 g, 28.9±1.0 weeks;  Mean values in standard nutrient group: 1156±234 g, 28.4±1.3 weeks  Followed up:  MRI scans and IQ data was available for 76 adolescents; 38 in the high nutrient, 38 in the standard nutrient group | Dose:  High-nutrient diet according to requirement  Formulation:  A preterm formula (Osterprem, Farley Health Products Ltd) containing 2.0 g protein and 80 kcal/100 ml and enriched with sodium, calcium, phosphorus, copper, zinc, vitamins D, E and K, water soluble vitamins, carnitine and taurine.  2.5 g protein/100 kcal | Dose:  Standard nutrient diet according to requirement  Formulation:  A standard term formula (Osterfeed = Ostermilk, Farley Health Products Ltd) containing 1.45 g protein and 68 kcal/100 ml or unfortified banked breast milk.  High-nutrient group received approximately 40% more protein and 20% more energy than standard nutrient group;  2.1 g protein/100 kcal | Duration:  Mean = 6 weeks  Started:  From birth; enteral feeds were increased according to tolerance to aim 180 ml/kg/d.  Ended:  When the infant reached the weight of 2000 g or was discharged from NICU. | Primary  None defined (no power calculation)  Cognition/ Behavior:  WISC-III, WAIS-III (VIQ, PIQ, FSIQ) at 8 and 16 yrs  Neuroimaging:  MRI;  Volumes for total brain, a series of subcortical gray matter structures, including the caudate nuclei and hippocampi. | VIQ and PIQ  The high-nutrient group had a signiﬁcantly higher VIQ (8 points) than the standard-nutrient group with no signiﬁcant differences in PIQ.  MRI  Diet was signiﬁcantly related to caudate volume only, the high-nutrient diet resulting in signiﬁcantly larger caudate nuclei bilaterally.  Left and right caudate nuclei showed strong relationships with VIQ but no signiﬁcant relationship with PIQ. |
| Lucas et al 1990  UK  RCT | Sample:  Preterm infants, BW<1850 g, in NICU, free from major congenital malformations.  Age & Weight:  Mean values in preterm formula group: 1379±43 g, 30.7±0.4 weeks; Mean values in standard term formula group: 1365±40 g, 30.8±0.4 weeks.  Randomized:  160; 79 in control group, 81 in intervention group  Followed up:  55/71 control group (82%) and 61/70 (87%) intervention group  8 deaths in control group, 11 deaths in intervention group | Dose:  Preterm formula containing 2.0 g protein and 80 kcal/100 ml and enriched with sodium, calcium, phosphorus, copper, zinc, vitamins D, E and K, water soluble vitamins, carnitine and taurine.  Formulation:  A preterm formula (Osterprem, Farley Health Products Ltd) as sole diet.  2.5 g protein/100 kcal | Dose:  Standard term formula containing 1.45 g protein and 68 kcal/100 ml.  Formulation:  A standard term formula (Osterfeed = Ostermilk, Farley Health Products Ltd) as sole diet.  2.1 g protein/100 kcal | Duration:  Approx. 30 days;  Median = 30 days (Control group), Median = 26 days (intervention group)  Started:  From birth; enteral feeds were increased according to tolerance to aim 180 ml/kg/d  Ended:  At 2000 g body weight or at discharge from NICU. | Primary  BSID at 18 months CA  Cognition/ Behavior  BSID (MDI and PDI);  Vineland Social Maturity Scale (SQ) at 18 months CA | BSID and SQ  Preterm formula fed children had consistently higher MDI, PDI and SQ values than term formula fed children. However, only PDI was statistically significant different between the groups, with a 14.7-point advantage (P<0.001) to the preterm formula fed group. |
| Lucas et al 1998  UK  RCT  (in trial A same study population as in Lucas et al 1990) | Sample:  Preterm infants, BW<1850 g, in NICU, free from major congenital malformations.  Age & Weight:  Mean values in different trial groups were not statistically significant and varied from 1354±66 g and 30.6±0.3 days to1425±28 g and 31.3±0.3 days  Randomized:  424, 160 in trial A, 264 in trial B  Followed up:  360/377 of survivors (96%)  47 who died, 19 were in trial A and 28 in trial B. | Dose:  Preterm formula according to requirement  Formulation:  A preterm formula (Osterprem, Farley Health Products Ltd) containing 2.0g protein and 80 kcal/100 ml; enriched with Na, Ca, P, Cu, Zn, vitamins D, E and K, water soluble vitamins, carnitine and taurine.  Trial A as sole diet;  Trial B as a supplement to mother’s breast milk;  2.5 g protein/100 kcal | Dose:  Standard term formula according to requirement  Formulation:  A standard term formula (Osterfeed = Ostermilk, Farley Health Products Ltd) containing 1.45 g protein and 68 kcal/100 ml;  Trial A as sole diet;  Trial B as a supplement to mother’s breast milk;  2.1 g protein/100 kcal | Duration:  Approx. 30 days;  Median = 26 to 33days  Started:  From birth; enteral feeds were increased according to tolerance to aim 180 ml/kg/d  Ended:  At 2000 g body weight or at discharge from NICU. | Primary:  Overall IQ at 7.5 to 8 years  Cognition/ Behavior:  WISC-R UK (VIQ, PIQ, FSIQ) at 7.5 to 8 yrs | WISC-R UK  VIQ was 4.8 points higher (P=0.8) in those fed solely a preterm formula (trial A) than in those fed standard term formula. No significant (P>0.05) group differences in VIQ, PIQ or FSIQ in trial A, B or both trials combined.  A major sex difference in the impact of diet was found. The beneficial effect of preterm formula on VIQ was seen for boys but not girls and was confined to those fed exclusively on the trial diets (trial A). Boys on the preterm formula had a 12.2 (95% CI, 3.7 to 20.6) point advantage in VIQ and a 6.3 (95% CI − 1.5 to 14.2) point advantage in FSIQ, whereas no effect was seen in girls.  Advantages in VIQ and FSIQ were even larger among boys who received the highest intake of trial diet (trial A and trial A plus B combined). The advantage was 14.4 (95% CI, 5.7 to 23.2) in VIQ and 9.5 (95% CI, 1.2 to 17.7) points in FSIQ for boys with the highest intakes of preterm formula. |

TABLE S4. PROTEIN AND AMINO ACID STUDIES

| Author, year, country, design | Study population | Intervention diet  (dose and formulation) | Control diet  (dose and formulation) | Intervention/ Observation period | Outcome measures | Effect of the intervention/  Findings of cohort study |
| --- | --- | --- | --- | --- | --- | --- |
| Protein fortification of infant formulas | | | | | | |
| Bhatia et al 1991  USA  RCT | Sample:  Premature infants, BW <1550 g, no major congenital anomalies, congestive heart failure, O_2_ requirement <40% inspired oxygen on study entry and no supplemental O_2_ on the day achieving 100 kcal/kg/d;  Age & Weight:  Mean values high protein group: 1335±142 g, 31.7±1.5 weeks;  medium protein group: 1335±138, 31.5±2.1 weeks;  control group: 1463±58 g, 32.7±1.3 weeks  Randomized:  26; 23 completed the feeding  Followed up:  18 were tested with BNBAS, 15 completed it; 6 in control group, 4 in low protein group, 5 in high protein group (58% (15/26)) | Dose:  High protein: 3.2 g protein/100 kcal or 3.8 g/Kg/day  Mid protein: 2.7 g protein/100 kcal or 3.1 g/Kg/day  Formulation:  Standard premature infant formula (Similac Special Care, Ross Laboratories, Columbus, Ohio)  Differences in protein content were adjusted by carbohydrate content. | Dose:  Low protein: 2.2 g protein /100 kcal or 2.6 g/Kg/day  Formulation:  Standard premature infant formula (Similac Special Care, Ross Laboratories, Columbus, Ohio) | Duration:  2 weeks  Started:  Enteral energy intake reached 100 kcal/kg/d;  at <21 days of age | Primary:  Growth, protein nutritional status at 37 weeks CA (within 5 days after intervention finished)  Cognition/ Behavior:  BNBAS (orientation, habituation, stability, range of state, regulation of state and motor) | BNBAS  Infants in the high protein group (3.2 g/100 kcal) performed significantly better on the orientation and habituation clusters of the BNBAS than infants fed 2.7 and 2.2 g protein/100 kcal.  The high protein group scored also significantly higher on the autonomic stability cluster of the BNBAS than the other two groups. |
| Goldman et al 1971  USA  RCT  (Follow-up study of Goldman et al 1969) | Sample:  Infants with BW<2000 g in the premature nursery free from major congenital, intestinal obstructions and Rh disease  Age & Weight:  Mean values not given;  199 infants with BW 1500-2000 g;  91 infants with BW 1000-1499 g;  14 infants with BW <1000 g  Randomized:  304; 152 in high protein and 152 in low protein group  Followed up:  234/293 surviving children (80%) | Dose:  Cow’s milk formula containing 4% protein, providing 6-7.2 g protein/kg/d; the feeds increased 15 ml /d until the full dose/d 150-180 ml  Formulation:  A formula (Similac) based on cow’s milk and containing 2% protein + 2% casein | Dose:  Cow’s milk formula containing 2% protein, providing 3-3.6 g protein/kg/d; the feeds increased 15 ml/ d until full dose/d 150-180 ml  Formulation:  A formula (Similac) based on cow’s milk containing 2% protein | Duration:  Approx. 4 weeks  Started:  At 24-72 hours of life  Ended:  At the reach of weight of 2200 g | Primary:  None defined (no power calculation)  Cognition/ Behavior:  Stanford-Binet test at 3 yrs chronological age | Stanford-Binet test (IQ)  High protein group: 41% of children had IQ scores <90 versus 31% in the low protein group.  Children with a BW<1300 g: 76% of children in the high protein group had IQ scores < 90 versus 23% in the low protein group (P<0.01).  Growth restricted children: more children with IQ scores <90 were in the high protein group than in the low protein group (47% vs 23%; P<0.05) |
| Goldman et al 1974  USA  RCT  (Follow-up study of Goldman et al 1969) | Sample:  Infants with BW<2000 g in the premature nursery free from major congenital, intestinal obstructions and Rh disease  Age & Weight:  Mean values not given;  199 infants with BW 1500-2000 g;  91 infants with BW 1000-1499 g;  14 infants with BW <1000 g  Randomized:  304; 152 in high protein and 152 in low protein group  Followed up:  237/292 surviving children (81%);  119 in the high protein, 118 in the low protein group | Dose:  Cow’s milk formula containing 4% protein, providing 6-7.2 g protein/kg/d; the feeds increased 15 ml /d until the full dose/d 150-180 ml  Formulation:  A formula (Similac) based on cow’s milk and containing 2% protein + 2% casein | Dose:  Cow’s milk formula containing 2% protein, providing 3-3.6 g protein/kg/d; the feeds increased 15 ml/ d until full dose/d 150-180 ml  Formulation:  A formula (Similac) based on cow’s milk containing 2% protein | Duration:  4 weeks  Started:  At 24-72 hours of life  Ended:  At the reach of weight of 2200 g | Primary:  None defined (no power calculation)  Cognition/ Behavior:  Stanford-Binet test at 5 -6 yrs of chronological age | Stanford-Binet test (IQ)  The number of IQ scores <90 was higher in the high protein group than in the low protein group (40% vs 29%; P>0.05)  Amongst children with a BW<1300g, 74% had IQ scores < 90 in the high protein group versus to 23% in the low protein group (P=0.01). |
| Protein fortification of breast milk | | | | | | |
| Biasini et al 2012  Italy  (R)CT | Sample:  ELBW infants  Age &Weight:  BW range: 580-1250 g, GA range: 23-32 weeks  Randomized?  61; 34 in high protein and 27 in control group;  Followed up:  19/34 (56%) in high protein group and 13/27 (48%) in control group  Cognitive assessment in ELBW infants weighing 580-980 g and GA 23-30 weeks. | Dose:  4.8 g protein/kg/d  (estimated max intake)  Formulation:  Fortified maternal or banked breast milk, breast milk fortifier (BMF) Aptamil, up to 5% plus 1% Protifar, Nutricia.  The energy intake 141 kcal/d | Dose:  3.5 g protein/kg/d.  Formulation:  Fortified maternal or banked breast milk, breast milk fortifier (BMF) Aptamil up to 5%  The energy intake 135 kcal/d | Duration:  Not reported  Started:  The first day of full enteral feeding.  Ended:  At the time of discharge or when the infant ingested> 50% of his/her prescribed quantity directly from the breast of the mother. | Primary:  None defined (no power calculation)  Neuroimaging (for screening):  MRI at 40 weeks of gestational age in 25/34 infants (high protein group) and 24/27 infants (control group)  To measure absence or presence neurological impairments: CP, index score< 80 at GMDS, deaf/hearing loss requiring amplification in either ears or bilateral blind. | GMDS  No significant difference between the two groups at 9 months of CA;  At 3 months of CA, the high protein group demonstrated a significant advantage (adjusted for CRIB) in the performance subscale (P=0.04).  MRI  One neonate in the control group demonstrated pathological images and later neurological impairments |
| Protein fortification of parenteral and enteral feeding | | | | | | |
| Cormack et al 2011  Australia  Cohort study | Sample:  Infants with BW <1500 g, NICU admission within 24h of birth and min NICU stay of 30 days;  Two cohorts: babies born before and after the change of IVN solutions (‘before’ group and ‘after’ group).  Age & Weight:  Mean values ‘before’ group: 893 g, 26.9±2.2 weeks;  Mean values ‘after group’: 946 g, 27.1±2.2 weeks  Non-randomized study:  80 infants; 40 infants per group  Followed up:  Bayley scores were available for 27/36 (75%) ‘before’ group infants and 37/40 (93%) ‘after’ group infants who survived to 18 months corrected age | Dose:  ‘After group’  IVN solution providing 103 kcal and 3.5 g protein per 180 ml/kg/d.  Formulation:  IVN solution (P100): TrophAmine as the amino acid (B Braun Medical Inc, Irvine, CA, USA) and containing amino acids, minerals and electrolytes more than standard IVN.  Enteral feed was aimed to start within 24 hours of birth and breast milk fortiﬁer, or preterm formula, was started once full enteral feeds were reached. | Dose:  ‘Before group’  IVN solution providing 100 kcal and 3.3 g protein per 180 ml/kg/d.  Formulation:  A standard IVN solution (P10) made up in 10% dextrose solution and containing amino acids, minerals and electrolytes.  Enteral feed was aimed to start within 24 hours of birth and breast milk fortiﬁer, or preterm formula, was started once full enteral feeds were reached. | Duration:  4 weeks  Started:  From birth  Full enteral feed ‘before’ group 10 days (8-12) and ‘after’ group 11 days (9-14) | Primary:  None defined (no power calculation)  Cognition/ Behavior:  BSID-III at 18 months CA | BSID-III  No differences in mean cognitive, motor development or language scores between the groups.    In the first two weeks, protein intakes were signiﬁcantly higher in the ‘after’ than in the ‘before’ group.  Mean enteral protein intake in the ﬁrst 2 weeks was positively associated with cognitive (r^2^ =0.13; P=0.03) and motor development (r^2^ =0.27; P=0.001), but not with language scores (P=0.08). |
| Cester et al 2015  New Zealand  Cohort study | Sample:  Infants with BW <1000 g, NICU admission within 24h of birth and min NICU stay of 30 days;  Two cohorts: babies born before and after the change of nutritional policy aiming to comply with the international consensus recommendations (‘before’ group and ‘after’ group).  Age & Weight:  Mean values ‘before’ group: 790 (520, 980) g, 26 (23, 31) weeks;  Mean values ‘after group’: 756 (470, 990) g, 26 (23, 31) weeks  Non-randomized study:  100 infants; 50 infants per group  Followed up:  37 infants (79% of survivors) in the ‘before’ group and32 (65% of survivors) infants in the ‘after’ group were available for the assessment of Bayley-III scores at 2 years CA | Dose/formulation:  ‘After group’: parenteral and enteral feeding started within 24 hours after birth (if not contraindicated).  The parenteral solution was enriched with amino acids and provided a maximum of 4 g AA/kg/day, the milk fortifier increased the milk protein content by 1 g protein/100 mL and was used once enteral feeds reached 5 mL per feed. Preterm formula (2.5 g protein/100 ml) was used for infants where mother milk was not available.  Average protein intake over the first month after birth was 3.8±0.3 g/kg/day | Dose/formulation:  ‘Before group’: parenteral and enteral feeding started within 24 hours after birth (if not contraindicated).  The parenteral solution provided a maximum of 3.8 g AA/kg/day; the milk fortifier increased the milk protein content by 0.8 g/100 ml and was used once full enteral feeds were attained. Preterm formula (2.0 g protein/100 ml) was used for infants where mother milk was not available.  Average protein intake over the first month after birth was 3.3±0.3 g/kg/day | Duration:  4 weeks  Started:  From birth  Full enteral feed in the ‘before’ group attained at 14 ± 7 days and in the ‘after’ group at 12 ± 4 days | Primary:  None defined (no power calculation)  Cognition/ Behavior:  BSID-III at 2 years CA | BSID-III  No differences in mean cognitive, motor development or language scores between the groups.  No significant associations between nutrient intakes and neurodevelopmental outcomes. |
| Stephens et al 2009  USA  Cohort study | Sample:  ELBW infants  Age & Weight:  Mean cohort values: 787 ±133g and 25.9±1.6 weeks;  43% boys and 13% SGA infants; Supplemental oxygen requirement: on average 57±33 days;  Ventilator support: on average 19±17 days;  NICU stay: on average 89±27 days  Non-randomized study:  148 infants    Followed up:  124 (84%) with BSID at 18 mo | Dose:  Daily total enteral and parenteral intakes during the first 4 weeks of life. Mean daily protein and energy intakes were calculated for weeks 1, 2, 3, and 4 based on what the infants actually received according to the feeding data.  Formulation:  Parenteral nutrition provided 3.4 kcal/g carbohydrate in a monohydrate form, 4.0 kcal/g protein as Trophamine, and 2.0 kcal/ml lipid as Intralipid 20% emulsion. Protein was started in the ﬁrst bag of parenteral nutrition at 1.0 g/kg per day and advanced by 0.5 g/kg per day up to 2.5 to 3.5 g/kg per day.  Enteral feeds were breast milk (nutritional content presumed that of mature breast milk with 20.0 kcal/oz and 1.54 g of protein per 100 kcal) with or without Similac Human Milk Fortiﬁer (additional 14.0 calories and 1.0 g of protein per 100 ml) or preterm formula initiated at 20.0 kcal/oz and advanced to 24 –27 kcal/oz once full enteral feeds were tolerated. The formulations of the 2 preterm formulas used provided 2.7 and 3.0 g of protein per 100 kcal. | | Duration:  4 weeks  Start:  From birth | Primary:  None defined (no power calculation)  Cognition/ Behavior:  BSID-II (MDI and PDI) at 18 months CA | BSID-II  The mean energy intake and the mean protein intake during the ﬁrst week of life contributed signiﬁcantly and independently to the MDI at 18 months.  During the first week of life each 10 kcal/kg/d increase in energy intake was associated with a 4.6-point increase in MDI, and each 1-g/kg/d increase in protein intake was associated with an 8.2-point increase in MDI at 18 months. |
| Early parenteral amino acid supplementation | | | | | | |
| Blanco et al 2012  USA  RCT | Sample:  ELBW infants, BW<1000 g, age <12hours, GA>24 weeks, free from major anomalies and imminent death  Age & Weight:  Mean values of the followed-up infants in the early and high AA group: 820±133 g, 26.5±1.9 weeks;  Mean values of the followed-up standard AA group: 805±145 g, 26.3±1.5 weeks  Randomized:  61; 31 in the standard AA and 30 in the early and high AA group  Followed up:  At 6 and 12 mo 43; 22 in the standard AA and 21in high and early AA group (69%);  At 18 and 24 mo 32; 16 in the standard AA and 16 in the high and early AA group (52%) | Dose and Formulation:  Early and high AA group  2.0 g/kg/d IV AA with 40 mg/kg/d of cystine hydrochloride in the total parenteral nutrition (TPN) solution, increasing 1 g/kg/d up to a maximum of 4 g/kg/d. TPN contained also lipids (Intralipid 20%, Fresenius Kabi AG Clayton R&D, NC), glucose, minerals, trace elements, and vitamins.  After the intervention both groups received TPN with 3.5 g/kg/d of AA | Dose and Formulation:  Standard AA group  0.5 g/kg/d IV AA (Aminosyn PF, Abbott Laboratories, Chicago, IL, USA) with 40 mg/kg/d of cystine hydrochloride in the total parenteral nutrition (TPN) solution; increasing 0.5 g/kg/d up to a maximum of 3 g/kg/d. TPN contained also lipids (Intralipid 20%, Fresenius Kabi AG Clayton R&D, NC), glucose, minerals, trace elements, and vitamins. | Duration:  7 days  Started:  During the first day of life  Ended:  After 7 days of life | Primary:  Reduction of potassium levels  Cognition/ Behavior:  BSID-II (MDI and PDI) at 6, 12, 18 and 24 months CA | BSID (MDI and PDI)  The test scores were similar in both groups at all test visits, except at 18 months: the MDI score was significantly lower in the early and high AA group, but this difference did not persist to 24 months. |
| Poindexter et al 2006  USA  Secondary analyses of a RCT | Sample:  ELBW infants, BW 401 g-1000 g, survived 36 weeks’ PMA  Age & Weight:  Mean values in early AA group: 805±128 g, 26.1±1.5 weeks;  Mean values in late AA group: 791±127 g, 26.0±1.5 weeks  Randomized:  A cohort of 1018 ELBW infants stratified according to early or late parenteral AA intake; 182 in the early AA and 836 in the late AA group  Followed up:  868/1018 (85%); 154/182 (85%) in the early AA group and 714/836 (85%) in the late AA group | Dose and Formulation:  Early provision of parenteral AA, i.e. >3 g AA/kg/d at<5 days of life; | Dose and Formulation:  Late provision of parenteral AA, i.e. parenteral AA later than at 5 days of life | Duration:  20 days  Started:  Within 72 hours from birth  Ended:  20 days later | Primary:  Risk of mortality and late-onset sepsis (in the original clinical trial)  Cognition/ Behavior:  BSID (MDI and PDI) at 18 months CA | BSID (MDI and PDI)  No group differences |

| Van den Akker et al 2014  The Netherlands  Secondary analyses of a RCT | | Sample:  VLBW infants, BW <1500, <2hours, GA<32 weeks  Age & Weight:  Not reported  Randomized:  A cohort of 135 VLBW infants randomized according to early or late parenteral AA intake: 66 in the early AA and 69 in the late AA group; 132 born <32 weeks GA were included in this study  Followed up:  111/114 survivors (96%); 54/56 (85%) in the early AA group and 57/58 (98%) in the late AA group for the primary outcome.  MDI was assessed in 37 (80%) and 36 (73%) of the nondisabled children in the late and early AA groups, respectively.  PDI results available in 34 (74%) and 32 (65%) of the nondisabled children in the late and early AA groups, respectively. | Dose and Formulation:  Early provision of glucose and parenteral AA, i.e. 2.4 g AA/kg/d from < 2 hours to 96 h after birth. Thereafter, infants in both groups received the same standard nutrition protocol | Dose and Formulation:  Late provision of parenteral AA, i.e. glucose alone during the first 24 to 48 postnatal hours, then the parenteral feeds were enriched with 1.2 g AA/kg/day; 24 hours later, the AA dose was increased to 1.2 g /kg/day | Duration:  4 days  Started:  Within 2 hours from birth  Ended:  3 days later | Primary:  Survival without major  disabilities at 2 years CA  Cognition/ Behavior:  BSID (MDI and PDI) at 2 years CA in those children without major disabilities | Disabilities:  No significant group differences when the complete cohort was considered. However, boys in the early AA group had higher odds of achieving a normal outcome.  MDI:  No significant group differences when the complete cohort was considered. However, in girls MDI scores were significantly lower in the early AA group after adjusting for potential confounders.  PDI:  No group differences. |
| --- | --- | --- | --- | --- | --- | --- | --- |
| TAURINE STUDIES | | | | | | | |
| Tyson et al 1989  USA  RCT | Sample:  Relatively healthy, formula fed preterm infants with BW < 1300 g;  Age &Weight:  Mean values intervention group: 1116±121 g, 30±2 weeks;  Mean values control group: 1154±109 g, 30±2 weeks  Randomized:  47 (23 intervention and 24 control group)  Followed up:  37 (19 intervention and 18 control group); (79%) | | Dose:  45 mg taurine/l  Formulation:  Similac Special Care formula (24 kcal/30 ml and 2.71 g of protein, 5.43 g of fat, and 10.6 g of carbohydrate per 100 kcal).  The volume of formula was determined by each infant’s tolerance and appetite.  Mean formula intake at study entry 120±42 ml | Dose:  < 5 mg taurine/l  Formulation:  Similac Special Care formula (24 kcal/30 ml and 2.71 g of protein, 5.43 g of fat, and 10.6 g of carbohydrate per 100 kcal).  The volume of formula was determined by each infant’s tolerance and appetite.  Mean formula intake at study entry 123±32 ml | Duration:  Approx. 4 weeks  Started:  7-10 days after birth (postnatal age)  Ended:  At discharge from hospital or the weight reached 2500 g | Primary:  Not well reported; Trial interrupted before reaching the planned sample size  Cognition/ Behavior:  BNBAS at 37 weeks CA  Electrophysiology:  ERG, ABR | BNBAS  Mean scores for taurine-supplemented and non-supplemented group were similar for all scales.  ERG  Similar responses were observed in the two feeding groups. Latency was not reduced in infants receiving higher taurine for wave A (cornea negative potential) or wave B (cornea positive potential).  ABR  The amplitude of waves was similar for both feeding groups. There was a modest but consistent difference between the two feeding groups (0.2 to 0.5 ms) in the latency at both stimulation rates. Latency was shorter in the higher taurine group. |
| Wharton et al 2004  UK  Epidemio-logical correlation study | Sample:  Preterm infants, BW<1850g, in NICU, free from major congenital malformations, with neonatal plasma taurine concentrations, BSID at 18 mo and WISC-R at 7 yrs available  Age & Weight:  Mean values in the cohort 1398±277 g and 31±2.4 weeks  157 children had the following information available: neonatal plasma taurine concentration, BSID at 18 mo and WISC-R at 7 yrs | | The study evaluated the minimum plasma taurine concentration as quartiles and the relationship with BSID and WISC-R | | The minimum plasma taurine concentration was measured during the neonatal period | Primary:  None defined (no power calculation)  Cognition/ Behavior:  BSID (MDI and PDI) and WISC-R at 18 months and 7 yrs CA | BSID and WISC-R  Minimum plasma taurine concentrations correlated with MDI (r=0.28, P<0.001) and the WISC-R arithmetic subtest score (r=0.22, P = 0.006). The associations remained significant after adjusting for possible confounding factors.  Minimum taurine was not related to the PDI nor to the other subtests of the WISC-R after adjusting for confounding factors.  Neither maximum nor mean taurine concentration was related to cognition.  The positive association of neurodevelopment with own mother’s milk, described previously, did not remain significant: partial correlations with MDI (r=0.03; P=0.70) and the arithmetic subtest (r=0.09; P=0.24) were found. |
| GLUTAMINE STUDIES | | | | | | | |
| van Zwol et al 2008  Netherlands  RCT  (Follow-up study from van den Berg et al 2005) | | Sample:  VLBW infants with GA< 32 weeks and/or BW< 1500 g in level 3 NICU  Age & Weight:  Mean values in glutamine group: 1180±370 g, 29.4±1.7 weeks;  Mean values in control group: 1170±310 g, 28.8±1.6 weeks  Randomized:  102; 52 in glutamine group, 50 in the control group  Followed up:  72 of the surviving 88 (82%).  40 in glutamine group, 32 in the control group | Dose:  0.3 g glutamine /kg/day  Formulation:  Enteral glutamine supplementation;  Glutamine powder (82% L-glutamine, 18% glucose (15.5% N, 371kcal/100g) added to breast milk or preterm formula | Dose:  0.3 alanine g/kg/day (isonitrogenous to glutamine)  Formulation:  Enteral alanine (isonitrogenous) supplementation;  Glutamine powder (82% L-glutamine, 18% glucose (15.5% N, 371 kcal/100 g) added to breast milk or preterm formula | Duration:  27 days  Started:  3^rd^ day of life  Ended:  30 day of life | Primary (original study):  Time to full enteral feeding  Cognition/ Behavior:  BSID-II (MDI and PDI) at 24 months CA | MDI and PDI:  No significant group differences, although MDI and PDI score were lower in the glutamine group.  Adjustment for sex, GA, BW, serious neonatal infection and postnatal corticosteroids did not change these results. |
| De Kievit et al 2012  Netherlands  RCT  (Follow-up study from van den Berg et al 2005) | | Sample:  Very preterm infants, GA <32 weeks and/or BW < 1500 g  Age & Weight:  Mean values intervention group: 1301± 380 g, 29.7±1.6 weeks; Mean values control group: 1204± 334 g, 29.0±1.6 weeks  Randomized:  102  Followed up:  64/89 survivors (72%) completed the tests | Dose:  0.3 g glutamine/kg/day  Formulation:  Enteral glutamine supplementation;  Glutamine powder (82% L-glutamine, 18% glucose (15.5% N, 371 kcal/100 g) added to breast milk or preterm formula. | Dose:  0.3 g alanine/kg/day  Formulation:  Enteral alanine (isonitrogenous) supplementation;  Alanine powder (100% L-alanine, 15.7% N, 435kcal/100g) added to breast milk or preterm formula. | Duration:  27 days  Started:  3^rd^ day of life  Ended:  30 day of life | Primary (original study):  Time to full enteral feeding  Cognition/ Behavior:  WISC-III at 7.5 yrs  MABC (dexterity, balance skills, ball skills)  CBCL, PDBD, TRF and TDBD | MABC  After adjusting for SES, GA, and serious neonatal infections, the glutamine group showed poorer ball skills (P = 0·002; Cohen’s d = 0·67).  WISC-III, CBCL, PDBD, TRF and TDBD:  No other signiﬁcant differences between the study groups for any other cognitive, motor or behavioral measures. |
| De Kievit et al 2012  Netherlands  RCT  (Follow-up study from van den Berg et al 2005) | | Sample:  Very preterm infants, GA <32 weeks and/or BW < 1500 g  Age & Weight:  Mean values intervention group: 1252± 380 g, 29.4±1.7 weeks; Mean values control group: 1186± 336 g, 28.9±1.7 weeks  Randomized:  102  Followed up:  53/89 survivors (60%) completed the MRI | Dose:  0.3 g glutamine/kg/day  Formulation:  Enteral glutamine supplementation;  Glutamine powder (82% L-glutamine, 18% glucose (15.5% N, 371kcal/100g) added to breast milk or preterm formula. | Dose:  0.3 g alanine/kg/day  Formulation:  Enteral alanine (isonitrogenous) supplementation;  Alanine powder (100% L-alanine, 15.7% N, 435 kcal/100 g) added to breast milk or preterm formula. | Duration:  27 days  Started:  3^rd^ day of life  Ended:  30 day of life | Primary (original study):  Time to full enteral feeding  Neuroimaging:  Brain volumes (MRI) and white matter integrity (fractional anisotropy-FA; DTI) at 8.5 years | MRI – Brain volumes:  After adjusting for age, gender, and BW for GA, the glutamine group had higher volumes of white matter, brain stem, and hippocampus than the placebo group. The glutamine group had also marginally significant higher putamen, total subcortical, and total intracranial volumes.  MRI - White matter integrity:  No significant group effect on the white matter FA values after adjusting for age, gender, and BW for GA. In an exploratory analysis without correction for multiple testing, the glutamine group had increased FA values in clusters located in the left and right cingulum  hippocampal tract. |

TABLE S5. LCPUFA STUDIES

| Author, year, country, design | Study population | Intervention diet  (dose and formulation) | Control diet  (dose and formulation) | Intervention/ Observation period | Outcome measures | Effect of the intervention/  Findings of cohort study |
| --- | --- | --- | --- | --- | --- | --- |
| LCPUFA and cognitive development | | | | | | |
| Fewtrell et al 2002  UK  RCT | Sample:  Preterm infants, BW<1750 g, GA <37 weeks; free from congenital malformations, tolerating enteral feedings at day 10, no breast milk.  Breast fed infants as a reference group.  Age & Weight:  Mean values in LCPUFA group: 1336±284 g, 30.4±2.3 weeks;  Mean values in the control group: 1353±274 g, 30.3±2.4 weeks  Randomized:  195; 95 in the LCPUFA group, 100 in the control group  Followed up:  175 (90%) completed at least 3 weeks of intervention;  158 (81%) at 9 mo, 158 (81%) at 18 mo | Dose:  11mg/100ml ARA and 6 mg/100ml DHA;  0.31% ARA and 0.17%DHA of total fatty acids  Formulation:  A supplemented formula (Prematil, with Milupan: LCPUFA-supplemented formula) containing a fat blend with vegetable oils (palm coconut, soya, sunflower) and milk fat with derivatives of linoleic and α-linolenic acid sourced from evening primrose oil (γ-linolenic acid) and egg lipids (ARA and DHA). | Dose:  No ARA or DHA  Formulation:  A preterm infant formula without additional LCPUFA (Prematil, Milupa: control formula) | Duration:  Mean duration = 33±17 days (control group) and 31±21 days (LCPUFA supplemented group)  Started:  At 10 days of life  Ended:  At discharge from hospital | Primary:  BSID-II (MDI and PDI) at 18 months post-term  Cognition/ Behavior:  Knobloch et al. Developmental Screening Inventory at 9 months post-term | BSID-II (MDI and PDI)  No significant difference between groups, although infants from the LCPUFA group had a 2.6-point advantage in Bayley MDI and a 2-point advantage in PDI.  Exploratory analyses showed a greater benefit of LCPUFA-supplemented formula in the most immature infants, i.e. those with GA <30 weeks. Infants had 4.5 point higher MDI and 5.8 point higher PDI scores than the control group.  Knobloch et al.  No significant difference between groups. |
| Woltil et al 1999  Netherlands  RCT | Sample:  Low risk infants with BW<2500 g  Age & Weight:  Mean values in formula group without LCPUFA: 2137 g, 36 weeks;  In formula group with LCPUFA: 2188 g, 37 weeks;  In the human milk group: 2030 g, 35 weeks  Randomized:  128; 75 in formula group without LCPUFA; 26 in formula with LCPUFA; 27 in the human milk group.  Followed up:  100% | Dose:  1. 0.17 mol EPA, 0.02 mol DPA, 0.20 mol DHA per 100 ml;  2. 0.34 mol EPA, 0.03 mol DPA and 0.43 mol DHA per 100 ml;  3. Maternal breast milk fed infants  Formulation:  Preterm formulas from Friesland Nutrition (Leeurwarden, NL); no differences in FA compositions of the formulas except LCPUFA; formula with LCPUFA supplemented with evening primrose oil (0.32%) and a single (n=13; 0.38%) or double (n=13; 0.80%) dosage of purified fish oil.  Single dosage: 0.17 mol EPA, 0.02 mol DPA and 0.20 mol DHA per 100 ml; double dosage: 0.34 mol EPA, 0.03 mol DPA and 0.43 mol DHA per 100 ml. | Dose:  No added LCPUFA in the preterm formula  Formulation:  All preterm formulas were from Friesland Nutrition (Leeurwarden, NL). There were no differences in FA compositions of the formulas except LCPUFA | Duration:  Mean duration = 35±7 days (preterm formula group without LCPUFA);  41±3 days (preterm formula with LCPUFA); 42±2 days (human milk group)  Started:  At birth  Ended:  42 days of life | Primary:  None defined (no power calculation)  Cognition/ Behavior:  BSID-II (MDI and PDI) at 19 months  Biological samples:  RBC fatty acid composition at 42 days | BSID (MDI and PDI):  No significant group difference in MDI.  PDI was significantly higher in infants who received preterm formula with double dosage of fish oil (n=13; 116±8) or breast milk (n=27; 113±11) compared to infants who received formula with single dosage of fish oil (n=13; 102±11).  PDI was also significantly correlated with n-3 LCPUFA (EPA and DHA) intake during days 0-42 in the infants who received the LCPUFA supplemented formula (n=26).  For infants who received formula (n=101) no correlation between MDI and RBC content of LCPUFA was found at day 42, but a significant correlation between PDI and RBC DHA and n-3 LCPUFA including DPA and EPA.  No significant correlations of BSID indices with RBC LCPUFA status in other groups. |
| Clandinin et al 2005  Canada  RCT | Sample:  Until discharge from hospital, preterm infants, GA <35 weeks; after discharge only infants with BW<1500g.  Age & Weight:  Mean values algal-DHA group:  1207±37 g, 29.7±0.3 weeks;  Mean values fish-DHA group:  1110±33 g, 28.9±0.3 weeks;  Mean values control group:  1179±35 g, 29.7±0.3 weeks  Randomized:  361; 112 in algal-DHA, 130 in fish-DHA and 119 in control group;  At discharge: 72 in algal-DHA, 90 fish-DHA and 83 in control group.  Followed up:  56 infants dropped out before discharge; 60 were excluded from the study at discharge.  245/361 (68%) continued after discharge;  179/245 (73%) completed the study (52 algal-DHA, 65 fish-DHA and 62 control) | Dose:  17mg DHA and 34mg ARA/100 kcal;  0.3% DHA and 0.6% ARA of total fatty acids (equivalent to the content of breast milk)  Formulation:  Standard ready to use formulas; premature formula (24 kcal/ oz) until discharge, discharge formula (22 kcal/oz) until 3 months after term, and term formula (20 kcal/oz) until 12 months after term.  1. algal-DHA group: DHA from algal oil and ARA from fungal oil (Martek Biosciences, Columbia, MD)  2. fish-DHA group: DHA from tuna fish oil (Roche Vitamins Inc, Parsippany, NJ) and ARA from fungal oil.  The algal-DHA formula similar to Enfamil Premature LIPIL with Iron, EnfaCare LIPIL, and Enfamil LIPIL with Iron (Mead Johnson & Company, Evansville, IN). | Dose:  No added DHA or ARA  Formulation:  Standard ready to use formulas; premature formula (24 kcal/ oz) until discharge, discharge formula (22 kcal/oz) until 3 months after term, and term formula (20 kcal/oz) until 12 months after term. | Duration:  12 months  Started:  Within the first 10 days of life, around 30 weeks of PMA  Ended:  At 92 weeks of PMA (12 months after term) | Primary:  Non-cognitive  Cognition/ Behavior:  BSID-II (MDI and PDI) at 118 weeks PMA | MDI and PDI:  The fish-DHA group had significantly higher mean Bayley MDI and PDI scores than did the control group. The algal-DHA group scored significantly better in the PDI whereas the higher scores in MDI compared to the control group did not reach the significant level. |
| Fang et al 2005^1^  Taiwan  RCT | Sample:  Healthy preterm infants, GA 30-37 weeks, no human milk given  Age & Weight:  Mean values LCPUFA group: 1980±110 g, 33.3±0.5 weeks;  Mean values control group: 1990±120 g, 33.0±0.5 weeks  Randomized:  28; 16 in the LCPUFA group, 11 in the control group (1 infant excluded after randomization)  Followed up:  BSID-II: 25/27 (93%) at 6 mo, 22/27 (81%) at 12 mo;  VEP: 24/27 (89%) at 4 mo, 23/27 (85%) at 6 mo;  Lea grating acuity cards,  Hiding Heidi ‘FACE’ cards: 27/27 (100%) | Dose:  Formula providing 0.05% DHA and 0.10% ARA  Formulation:  Supplemented formula, Neoangelac Plus by Multipower Enterprise providing 0.05% of DHA and 0.10% ARA.  Infants given >110 kcal/kg/d during the first 4 months and >70 kcal/kg/d from 4 to 6 months. | Dose:  No added DHA and ARA  Formulation:  Unsupplemented formula, Neoangelac by Multipower Enterprise providing an adequate ratio of linoleic acid: α-linolenic acid (10:1).  Infants given >110 kcal/kg/d during the first 4 months and >70 kcal/kg/d from 4 to 6 months. | Duration:  6 months  Started:  Approximately 2 weeks after birth;  Mean GA at study entry in the LCPUFA group 35.6±0.2 weeks and in the control group 35.5±0.2  Ended:  6 months later | Primary:  Not defined (no sample size calculation)  Cognition/ Behavior:  BSID-II; MDI and PDI at 6 and 12 months after study entry  Electrophysiology/ Vision:  VEP, Lea grating acuity cards,  Hiding Heidi ‘FACE’ cards at 4 and 6 months after study entry | BSID-II  At 6 mo and 12 mo MDI and PDI scores were significantly higher (by repeated measures of ANOVA) in the LCPUFA group than in the control group.  VEP, Lea grating acuity cards,  Hiding Heidi ‘FACE’ cards  No significant differences between feeding groups at 4 mo or at 6 mo. |
| Van Wezel-Meijler, 2002^1^  Netherlands  RCT | Sample:  Preterm infants in NICU, GA<34 wk, BW<1750 g, with normal neurological examination, free from significant cerebral damage, retinopathy, chronic disease or feeding problems; mothers not breast feeding  Age & Weight:  Mean values in LCPUFA group: 1282±316 g, 30.4±1.5 wk;  Mean values in the control group: 1306±257 g, 30.4±1.6 wk  Randomized:  42  Followed up:  42/42 (100%) | Dose:  0.34% DHA and 0.68% ARA of total fat.  Formulation:  Parenteral nutrition with negligible amounts of LCPUFA before starting enteral feeding with preterm formula.  LCPUFA (DHASCO oil, produced by microalgae and ARASCO oil, produced by fungi; Martek Inc., Columbia, USA) supplemented preterm formula (Nutricia, Zoetermeer, The Netherlands) until weight of 3000g.  Thereafter LCPUFA supplemented term infant formula. | Dose:  No added DHA or ARA  Formulation:  Parenteral nutrition with negligible amounts of LCPUFA before starting enteral feeding with preterm formula.  Standard preterm formula (Nutricia, Zoetermeer, The Netherlands) until weight of 3000 g.  Thereafter standard unsupplemented infant formula | Duration:  8-9 months  Started:  3-7 days from birth  (full enteral feeding was achieved between 28 and 35 days of life)  Ended:  6 months of CA | Primary:  Not defined (no sample size calculation)  Cognition/ Behavior:  BSID (MDI, PDI) at 3, 6, 12 and 24 mo CA  Electrophysiolog/ Vision:  Flash-VEP at 3 and 12 mo CA;  Visual acuity by Teller card procedure at 3, 6, 12 and 24 mo CA  Neuroimaging:  MRI (global and cerebral visual system myelination) at 3 and 12 mo CA | MRI:  No significant differences in global or visual myelination at 3 mo or 12 mo or the progress of myelination from 3 mo to 12 mo between DHA and control groups.  Flash-VEP:  No significant differences at either test age between the DHA and control groups.  BSID:  There was a tendency towards better scores for MDI and PDI in the control group. Based on repeated measurement analysis the differences were not significant for MDI, whereas there was a systematic significant difference between the two groups with respect to PDI (P = 0.04) in favor of the control group, which disappeared after adjustment for BW and number of small-for-gestational-age infants (P=0.8).  Visual acuity:  At all test ages mean acuity values were slightly higher, but not statistically significant, for the DHA versus the control group. Group differences did not change between 3 and 24 mo CA, so visual acuity did not increase more rapidly in the DHA group. |
| Smithers et al 2010  Australia  RCT  (same study population as in Smithers et al 2008,  a pilot study to Makrides et al 2009) | Sample:  Infants with GA<33 weeks, free from major congenital or chromosomal abnormalities; infants with medical comorbidities common to preterm infants were included.  Randomized:  143; 74 in the high DHA group and 69 in the control group  Followed up:  128/143 (90%) at 26 mo;  125/143 (87%) at 3-5 yrs | Dose:  1% DHA  Formulation:  A combination of breast milk and formula  Breast milk: Lactating mothers consumed six 500-mg DHA-rich tuna oil capsules/d to achieve a breast milk DHA concentration approximately 1% of total fatty acids without altering the naturally occurring concentration of ARA in breast milk.  or  Preterm formula: containing approx. 1.0% DHA and 0.6% ARA. | Dose:  0.2-0.3% DHA  Formulation:  A combination of breast milk and formula  Breast milk: Lactating mothers consumed six 500-mg soy oil capsules that did not change the fat content or fatty acid composition of their milk.  or  Standard preterm formula: containing approximately 0.2-0.3% DHA and 0.6% ARA. | Duration:  Approx. 10 weeks  Started:  Asap after birth but within 5 days of receiving any enteral feeds  Ended:  At term expected delivery date | Primary:  Powered for MCDI Vocabulary Production subscale at 26 months and SDQ Total Difficulties score at 3-5 yrs  Cognition/ Behavior:  MCDI at 26 mo;  SDQ and STSC at 3-5 yrs | MCDI:  No significant difference between the high DHA and control group in the Vocabulary Production score or in any other MCDI subscale scores.  SDQ:  No significant difference between the high DHA and control group in the Total Difficulties scores or in any other SDQ subscales.  STSC:  No significant difference in the number of children with difficult temperament between the groups. |
| Makrides et al 2009  Australia  RCT | Sample:  Infants with GA<33 weeks, free from major congenital or chromosomal abnormalities  Age & Weight:  Mean BW and median GA in the high DHA diet group: 1308±423 g, and 30 weeks;  Mean BW and median GA in the standard DHA diet group: 1307±415 g, 30 weeks  Randomized:  657; 322 in the high DHA diet group and 335 in the standard DHA diet group  Followed up:  614/657 (93.5%); 298/322 (92.5%) in the high DHA diet group;  316/335 (94.3%) in the standard DHA diet group | Dose:  1% DHA of total fatty acids  (Measured DHA in breast milk 0.85±0.39% and in preterm formula 1.11±0.29%)  Formulation:  Breast milk: Lactating mothers consumed six 500-mg DHA-rich tuna oil capsules/d (Clover Corporation, Sydney, Australia) to achieve a breast milk DHA concentration approximately 1% of total fatty acids without altering the naturally occurring concentration of ARA in breast milk.  or  Preterm formula: containing approximately 1.0% DHA and 0.6% ARA. | Dose:  0.35% DHA of total fatty acids  (Measured DHA in breast milk 0.25±0.13% and in preterm formula 0.42±0.05%)  Formulation:  Breast milk: Lactating mothers consumed six 500-mg soy oil capsules (Clover Corporation, Sydney, Australia) that did not change the fat content or fatty acid composition of their milk.  or  Standard preterm formula: containing approximately 0.35% DHA and 0.6% ARA. | Duration:  Approx. 10 weeks  Started:  Day 2-4 of life  Ended:  At term of CA | Primary:  BSID-II (MDI) at 18 months CA  Cognition/ Behavior:  BSID-II (PDI);  Home Screening Questionnaire  At 18 months CA | MDI:  No significant difference between the high DHA and standard DHA group.  There were interactions between dietary treatment and sex; the MDI score among girls fed high DHA diet was significantly higher than among girls fed the standard DHA diet; adjusted mean difference 4.5(0.5-8.5)  PDI:  No significant difference between diet groups.  Home Screening Questionnaire  No significant difference between diet groups. |
| Fewtrell et al 2004  UK  RCT | Sample:  Unselected population (human milk and formula milk fed) of preterm infants, BW<2000 g, GA < 35 weeks, in NICU, free from congenital malformations, receiving at least some of their enteral feeds as formula milk during their hospital stay.  Age & Weight:  Mean values in the LCPUFA group: 1487±342 g and 31.2±2.1 weeks;  Mean values in the control group: 1510±326 g and 31.1±1.9 weeks  Randomized:  238; 122 in the LCPUFA group and 116 in the control group  Followed up:  199/238 (84%);  106/122 (87%) in the LCPUFA group and 93/116 (80%) in the control group | Dose:  0.9% GLA and 0.5% DHA of total fat in formulas  41 mg GLA/100 ml and 23 mg DHA/100 ml in preterm formula  36 mg GLA/100 ml and 20 mg DHA/100 ml in post-discharge formula  Formulation:  Preterm infant formula, OsterPrem [H. J. Heinz Co, Ltd, Hayes, Middlesex, UK] with LCPUFA, until weight 2 kg or discharge from hospital.  After discharge a nutrient-enriched post-discharge formula, Farley’s PremCare with LCPUFA.  The fat was a blend of vegetable oils (high oleic sunﬂower oil, palmolein, palm kernel oil, and canola oil).  LCPUFA were sourced from borage (starﬂower) oil (GLA) and tuna ﬁsh oil, providing a high DHA/EPA ratio. | Dose:  No added GLA or DHA  Formulation:  Preterm infant formula, OsterPrem [H. J. Heinz Co, Ltd, Hayes, Middlesex, UK] until weight 2kg or discharge from hospital.  After discharge, a nutrient-enriched post-discharge formula, Farley’s PremCare.  The fat was a blend of vegetable oils (high oleic sunﬂower oil, palmolein, palm kernel oil, and canola oil). | Duration:  Approx. 10 months  Started:  When the infants were able to tolerate formula.  Mean age of days in the LCPUFA group 14.3±9.6 days and in the control group 13.9±10.4 days  Ended:  9 months after term | Primary:  BSID (MDI, PDI) at 18 months post-term  Cognition/ Behavior:  BSID (MDI, PDI);  Knobloch et al. Developmental Screening Inventory at 18 months post-term | BSID-II  No significant difference in MDI and PDI scores between the LCPUFA and control group.  Boys in the LCPUFA group had signiﬁcantly higher MDI scores than boys in the control group (difference, 5.7 points [95% CI, 0.3-11.1; P=0.04), and there was a signiﬁcant interaction between diet and sex on MDI score (P=0.05).  Knobloch et al. Developmental Screening Inventory  There were no significant differences in overall developmental scores and individual subscale scores between randomized groups. |
| Isaacs et al 2011  UK  RCT  (10-year follow up study of Fewtrell et al 2004) | Sample:  Preterm infants, BW<2000 g, GA<35 weeks, in NICU, no congenital malformations and receiving at least some feedings as formula milk during hospitalization  Age & Weight:  Mean values in the LCPUFA group: 1454±369 g, 30.6±2.3 weeks;  Mean values in the control group: 1512±338 g, 30.9±2.0 weeks  Randomized:  238; 122 in the LCPUFA group and 116 in the control group  Followed up:  107; 50 in the LCPUFA group and 57 in the control group;  107/238 (45%) | Dose:  Formulas containing 0.5% DHA and 0.9% GLA  Formulation:  Preterm infant formula, OsterPrem [H. J. Heinz Co, Ltd, Hayes, Middlesex, UK] with LCPUFA, until weight 2 kg or discharge from hospital.  After discharge a nutrient-enriched post-discharge formula, Farley’s PremCare with LCPUFA.  The fat was a blend of vegetable oils (high oleic sunﬂower oil, palmolein, palm kernel oil, and canola oil).  LCPUFA were sourced from borage (starﬂower) oil (GLA) and tuna ﬁsh oil, providing a high DHA/eicosapentaenoic acid ratio. | Dose:  No added DHA or GLA in formula  Formulation:  Preterm infant formula, OsterPrem [H. J. Heinz Co, Ltd, Hayes, Middlesex, UK] until weight 2kg or discharge from hospital.  After discharge, a nutrient-enriched post-discharge formula, Farley’s PremCare.  The fat was a blend of vegetable oils (high oleic sunﬂower oil, palmolein, palm kernel oil, and canola oil). | Duration:  Around 10 months  Started:  When the infants were able to tolerate formula.  Mean age of days in the LCPUFA group 14.3±9.6 days and in the control group 13.9±10.4 days  Ended:  9 months after term | Primary:  None  Cognition/ Behavior:  BSID (MDI, PDI) at 18 months post-term;  WASI (VIQ, PIQ, FSIQ), the Neuropsychological Test for Children, CMS, WIAT-II, the Test of Everyday Attention for Children, the Behavioral Assessment of the Dysexecutive Syndrome for Children at 10 months | WASI (VIQ, FSIQ) and CMS  For children who had received only formulas in infancy the LCPUFA group had significantly higher scores in VIQ and FSIQ and word pair learning scores of the CMS than the control group.  WIAT-II  Signiﬁcant differences between diet groups were seen only for girls and only for 2 measures of academic attainments; word reading (P=0.07) and spelling score (P=0.02)  Other cognitive tests  There were no significant differences between the randomized control and LCPUFA groups with respect to general and speciﬁc cognitive outcomes. |
| O’Connor et al. 2001^1^  USA, UK, Chile  RCT | Sample:  Preterm infants, GA<33 wk, BW 750 -1805g, free from serious congenital abnormalities, periventricular or intraventricular hemorrhage and no major surgery or maternal incapacity, liquid ventilation, asphyxia resulting in severe and permanent neurologic damage, or uncontrolled systemic infection at the time of enrolment.  Age & Weight:  Mean values control group: 1287±272 g, 29.6±1.9 wk;  Mean values fish/fungal oil group: 1305±293 g, 29.8±2.1wk;  Mean values egg-TG/fish group: 1309±286 g and 29.7±2.0wk  Randomized:  470  Followed up:  376/470 (80%) at 12 mo CA | Dose:  From birth to term:  1. 0.43% ARA + 0.27% DHA and 0.08% EPA (fish/fungal oil)  2. 0.41% ARA + 0.24% DHA and 0% EPA (egg-TG/fish oil)  From term to 12 mo of CA:  1. 0.43% ARA + 0.16% DHA and 0% EPA (fish/fungal oil)  2. 0.41% ARA + 0.15% DHA and 0% EPA (egg-TG/fish oil)  Formulation:  Human milk and/or the assigned in-hospital pre-term formula (modified version of Similac Special Care ready-to-feed [24 kcal/fl oz]; SSC) with ARA- and DHA-enriched oils until term CA.  From term CA, infants received post-discharge nutrient-enriched formula (modified version of NeoSure powder [22 kcal/fl oz]) with the same sources of ARA+DHA and/or human milk. | Dose:  No supplemental ARA or DHA  Formulation:  Human milk and/or the assigned in-hospital pre-term formula (modified version of Similac Special Care ready-to-feed [24 kcal/fl oz]; SSC) without ARA- and DHA-enriched oils until term CA.  From term CA, infants received post-discharge nutrient-enriched formula (modified version of NeoSure powder [22 kcal/fl oz]) without the sources of ARA+DHA and/or human milk. | Duration:  Around 14 months  Started:  Within 72 hours of the first enteral feeding (infants could be enrolled as long as enteral feeding was initiated by the 28th day of life).  Ended:  12 months of CA | Primary:  BSID (MDI and PDI) at 12 months of CA  Cognition/ Behavior:  The Fagan Test of Infant Intelligence at 6 and 9 mo CA;  MCDI vocabulary checklist at (9 and 14 mo CA  Electrophysiology/ Vision:  VEP at 4 and 6 mo CA;  Teller Acuity Card Procedure at 2, 4, and 6 mo CA | Teller Acuity Card Procedure:  No significant group difference at 4 and 6 mo CA.  VEP:  No significant group difference at 4 mo CA. At 6 mo CA, the mean VEP acuity was significantly greater in both intervention (ARA + DHA) groups than in the control group. The mean VEP acuity of infants in the ARA + DHA-supplemented group increased between 4 and 6 mo CA, but the mean VEP acuity of those in the control group did not.  BSID:  No significant differences between groups for MDI. A statistically significant feeding by BW stratum interaction was found for PDI (P=0.005) in infants who consumed >80% of their feeding as study formula and/or human milk.    Fagan Test of Infant Intelligence:  The mean novelty preference was significantly greater in the ARA + DHA (egg-TG/fish)-group than in the control and ARA + DHA (fish/fungal)-group at 6 mo CA.  MCDI:  No significant group difference at 9 and 14 mo CA. |
| Henriksen et al 2008  Norway  RCT  (same study population as in Westerberg et al 2011) | Sample:  VLBW infants, BW<1500g, receiving human milk, free from major congenital abnormalities and cerebral hemorrhage  Age & Weight:  Median values in the LCPUFA group: 1090 g, 28.4 weeks  Median values in the control group: 1090 g, 28.9 weeks  Randomized:  141; 68 in the LCPUFA group, 73 in the control group  Followed up:  129 (91%) completed the intervention; 62 in the LCPUFA group and 67 in the control group;  105 (74%) completed the ASQ; 50 in the LCPUFA group, 55 in the control group;  EEG recordings were received from 81 (57%) infants | Dose:  DHA and ARA (48 mg/kg/d of each);  Mean daily intake of DHA 59 mg/kg and 47 mg/kg of ARA  (≈1.4% DHA and ≈1.2% ARA of total fatty acids)  Formulation:  Breast milk from mother or donor;  ARA and DHA as triacylglycerol (Martek Biosciences, Columbia, MD) mixed with soy oil and medium-chain triglyceride oil containing 6.9% (wt/wt) DHA and 6.9% (wt/wt) ARA, and providing 32 mg of DHA and 31 mg of ARA in 0.5 ml/100 ml of human milk. | Dose:  No added ARA and DHA;  Mean daily intake of DHA 32mg/kg and 22mg/kg of ARA  (0.7% DHA and 0.5% ARA of total fatty acids)  Formulation:  Breast milk from mother or donor;  The same mixture of soy oil and medium-chain triglyceride oil as the study group but without DHA or ARA. | Duration:  Mean = 9 weeks  Started:  When the enteral feeding was at least 100ml/kg/d (within the first week of life)  Ended:  At discharge from hospital | Primary:  ASQ at 6 months CA  Electrophysiology:  EEG; assessment of ERP at 6 months CA | ASQ  The intervention group scored higher on the problem-solving scale than the control group (53.4 vs 49.5 points; P=0.02). A non-signiﬁcantly higher total score (221 vs 215 points) was found.  EEG/ERP  Infants in the intervention group had signiﬁcantly lower (more-negative) amplitudes the standard image compared to the control group (P=0.01).  Presentations of novel images (deviants) did not show any group difference (P=0.14). |
| Westerberg et al 2011  Norway  RCT  (same study population as in Henriksen et al 2008) | Sample:  VLBW infants, BW<1500 g, receiving human milk, free from major congenital abnormalities and cerebral hemorrhage  Age & Weight:  Mean values in LCPUFA group: 1042±273 g, 28.7±2.9wk;  Mean values in the control group: 1067±300 g, 28.9±2.7wk.  Randomized:  141; 68 in the LCPUFA group and 73 in the control group  Followed up:  129 (91%) completed the intervention, 62 in the LCPUFA group and 67 in the control group;  92/129 (71%) participated in the cognitive tests;  80/129 (62%) completed the ‘free-play sessions’. | Dose:  Mean daily intake of DHA 59 mg/kg and 47 mg/kg of ARA  (≈1.4% DHA and ≈1.2% ARA of total fatty acids)  Formulation:  Breast milk from mother or donor;  ARA and DHA as triacylglycerol (Martek Biosciences, Columbia, MD) mixed with soy oil and medium-chain triglyceride oil containing 6.9% (wt/wt) DHA and 6.9% (wt/wt) ARA, and providing 32 mg of DHA and 31 mg of ARA in 0.5 ml/100 ml of human milk. | Dose:  Mean daily intake of DHA 32 mg/kg and 22 mg/kg of ARA  (0.7% DHA and 0.5% ARA of total fatty acids)  Formulation:  Breast milk from mother or donor;  The same mixture of soy oil and medium-chain triglyceride oil as the study group but without DHA or ARA. | Duration:  Mean = 9 weeks  Started:  When the enteral feeding was at least 100ml/kg/d (within the first week of life)  Ended:  At discharge from hospital | Primary:  ASQ at 20 months CA  Cognition/ Behavior:  Attention capacity by two ‘free-play sessions’;  BSID-II (MDI) at 20 months chronological age and CA | ASQ:  No significant differences between the LCPUFA and control group for sub-scores or total score.  Free-play sessions:  The intervention group had significantly more time sequences with a high level of attention and improved ability to Sustained Attention towards the play objects than the control group.  A positive correlation was found between the maximum number of sequences in a row with a high level of attention and plasma level of DHA at discharge (n = 52, r=0.29; P=0.04).  BSID-II (MDI):  No significant group difference. |
| Almaas et al 2015  Norway  RCT  (same study population as in Henriksen et al 2008) | Sample:  VLBW infants, BW<1500 g, receiving human milk, free from major congenital abnormalities and cerebral hemorrhage  Age & Weight:  Mean values in LCPUFA group: 1042±273 g, 28.7±2.9wk;  Mean values in the control group: 1067±300 g, 28.9±2.7wk.  Randomized:  141; 68 in the LCPUFA group and 73 in the control group  Followed up:  129 (91%) completed the intervention, 62 in the LCPUFA group and 67 in the control group;  98/129 (76%) participated in the cognitive tests;  84/129 (62%) participated to the MRI | Dose:  Mean daily intake of DHA 59mg/kg and 47mg/kg of ARA  (≈1.4% DHA and ≈1.2% ARA of total fatty acids)  Formulation:  Breast milk from mother or donor;  ARA and DHA as triacylglycerol (Martek Biosciences, Columbia, MD) mixed with soy oil and medium-chain triglyceride oil containing 6.9% (wt/wt) DHA and 6.9% (wt/wt) ARA, and providing 32 mg of DHA and 31 mg of ARA in 0.5 ml/100 ml of human milk. | Dose:  Mean daily intake of DHA 32 mg/kg and 22 mg/kg of ARA  (0.7% DHA and 0.5% ARA of total fatty acids)  Formulation:  Breast milk from mother or donor;  The same mixture of soy oil and medium-chain triglyceride oil as the study group but without DHA or ARA. | Duration:  Mean = 9 weeks  Started:  When the enteral feeding was at least 100ml/kg/d (within the first week of life)  Ended:  At discharge from hospital | Primary:  None defined (no posthoc power calculation)  Cognition/ Behavior:  WASI (VIQ, PIQ, FSIQ), WISC-III Digit Span, CVLT-II, the Grooved Pegboard test at 8.5 years  Neuroimaging:  MRI (neuroanatomical volumes; cortical volume, surface area, and  thickness) at 8.5 years | Cognition/Behavior:  No significant differences between the LCPUFA and control group in any of the tests.  MRI:  No significant group differences in any of the studied parameters. |
| Lundqvist-Persson et al 2010  Sweden  Cohort study  (same cohort as in Sabel et al 2012) | Sample:  Preterm infants, GA 24-36 weeks,  Age & Weight:  Mean values in the cohort: 2072 g, 34 weeks  Non-randomized study:  51 infants; 23 boys and 28 girls  Followed up:  44/51 (86%) tested with GM at 40 wk;  29/51 (57%) tested with BNBAS at 40 wk;  12/51 (24%) tested with BNBAS at 44 wk. | The main EFA concentrations in breast milk, and in mothers’ and infants’ plasma phospholipids during early post-natal period.  At term age (40 weeks of GA), corresponding to 3–15 weeks of age in the infants, 90% of the infants were still fully breast fed and the remaining 10% got at least 80% of their intake as breast milk. Four weeks later at 44 weeks of GA, 78% of the infants were fully breast-fed and the remaining infants were breast fed to less than 25%, or were fully formula fed. | | Started:  Immediately after birth  Ended:  44 weeks of GA | Primary:  None defined (no power calculation)  Cognition/ Behavior:  General Movements (GM) at 40 weeks GA;  BNBAS at 40 and 44 weeks GA;  Self-Regulation Scale at 40 and 44 weeks GA | GM:  The motor quality was negatively associated with Mead acid and EFA deficiency index (Mead acid/ARA) in breast milk.  BNBAS:  ARA in breast milk was negatively associated with Orientation and Range of States.  Self-Regulation Scale:  No significant increase in the level of self-regulation. |
| Sabel et al 2012  Sweden  Cohort study  (same cohort as in Lundqvist-Persson et al 2010) | Sample:  Preterm infants, GA 32-35 weeks; 57% of infants were late preterm with GA 34-36.9 weeks  Age & Weight:  Median GA for the group: 34 weeks  Non-randomized study:  51 non-randomized infants; 23 boys and 28 girls  Followed up:  Breast milk from 50/51 (98%) mothers;  45/51 (88%) plasma samples from infants at 44 wk of GA | 1. the concentrations of essential fatty acids (EFA) and LCPUFA in early breast milk one week after delivery  2. the concentrations of essential fatty acids (EFA) and LCPUFA in infants’ plasma phospholipids at 44 weeks of GA | | The median length of mother’s exclusive breast feeding was 3 months (IQR 0-6 mo)  and  partly breastfeeding 6 months (IQR 3-9.5 mo) | Primary:  None defined (no power calculation)  Cognition/ Behavior:  GM at 3 months CA;  BSID-II (MDI, PDI and BRS) at 3, 6, 10 and 18 months CA | BSID-II:  LA, the major n-6/n-3 FA ratios, Mead acid and the EFA deficiency index in early breast milk were negatively associated with development up to 18 months of age. DHA and ARA, respectively, in infants’ plasma phospholipids were positively but the ARA/DHA ratio negatively associated with development from 6 to 18 months of age.  GM:  No significant association in multiple regression analysis when adjusted for confounders. |
| Bialecka-Pikul et al 2014  Poland  Cohort study | Sample:  Preterm infants, GA 24-28 weeks  Age & Weight:  Median weight for the group: 998 g  Non-randomized study:  36 non-randomized infants; 20 boys and 16 girls; the study group (n=16-23, born between April and October 2008) was compared to a control group (n=9-13, born between October 2007 and April 2008)  Followed up:  38 months of GA | Parenteral lipid emulsion with DHA during the first 3-4 weeks | | Parenteral lipid emulsion without DHA during the first 3-4 weeks | Primary:  None defined (no power calculation)  Cognition/ Behavior:  Social-Emotional and Adaptive Behavior Scales of the BSID, Children’s Developmental Scale (Polish version of the BISD), Vocabulary Test-Comprehension of the Peabody Picture Vocabulary Test at 38 months CA | No significant differences between the DHA and non-DHA supplemented groups in any of the tests. |
| LCPUFA and visual development | | | | | | |
| Bougle et al 1999  France  RCT | Sample:  Healthy infants, GA<34 weeks; free from respiratory, metabolic or neurological disease, malformation and infection; enteral feeding in place within the first 7 days of life.  Age & Weight:  Mean values in breast milk group: 1847±394 g, 32.3±1.3 weeks;  Mean values in PUFA group (A): 1663±377 g, 32.2±1.2 weeks,  Mean values in LCPUFA group (B): 1835±438 g, 32.9±1.3 weeks  Randomized:  40 enrolled; 25 randomized (11 in group A and 14 in group B) and 15 in breast milk group  Followed up:  33/40 (83%) (11 in breast milk, 9 in A and 13 in B group) | Dose:  1. 14.1% LA and 1.3% ALA of total fatty acids  2. 17.7% LA, 0.1 % ARA, 1.2% ALA, 0.1% EPA and 0.6% DHA of total fatty acids  Formulation:  1. preterm formula with PUFA (group A)  2. preterm formula with LCPUFA (group B)  Intakes were recorded daily, supplementation of breast milk and formulas with dextrins, proteins or minerals, but not with lipids was allowed as required. | Dose:  14.1% LA, 0.9% ARA, 0.5% ALA and 0.5% DHA  Formulation:  Breast milk  All the 3 groups were given a daily supplementation of 1200 U of Vit D and 4.5mg of Vit E (Uvesterol ADEC, Laboratoires Crinex | Duration:  At least 30 days  Started:  Within the first 2 days of enteral feeding (within the first week of life).  Ended:  At the expected date of delivery (>36 weeks of PMA) after infants had been fed for at least 30 days on the study diet. | Primary:  None defined (no power calculation)  Electrophysiology/ CNS maturation:  VEP, BAEP and NCV at around 37.5 days of life | VEP and BAEP:  No significant differences between breast milk, PUFA or LCPUFA formula groups.  NCV:  The maturation of motor nerve conduction velocity was significantly slower in the LCPUFA group than in the PUFA or breast milk group. |
| Innis et al 2002  USA/Canada  RCT | Sample:  Healthy VLBW infants, BW 846-1560 g;  A group of breast-fed term infants with GA 38-42 weeks as reference.  Age & Weight:  Mean values in the control group: 1230±180 g, 29.5±1.7weeks;  Mean values in the DHA group: 1270±170 g, 30.0±1.4 weeks;  Mean values in the DHA+ARA group: 1280±180 g, 29.7±1.7 weeks  Randomized:  194; 62 in the control, 66 in the DHA and 66 in the DHA+ARA group;  A non-blinded breast-fed group of term infants as reference group with human milk intake >85% for 4 months.  Followed up:  173/194 (89%) completed the formula feeding phase | Dose:  1. 0.34% DHA of total fatty acids (~0.15% of energy)  2. 0.33% DHA and 0.60% ARA of total fatty acids (0.14% and 0.27% of energy)  Formulation:  Formulas were similar to Enfamil Premature Formula (Mead Johnson Nutritionals, Evansville, Ind) providing 81.3kcal/100ml with 3 g protein, 11.1 g carbohydrate, and 5.1 g fat/100kcal, enriched with DHA or DHA and ARA  DHA was provided as oil from the alga Crypthecodinium cohnii and ARA as oil from the fungus Mortierella alpina (Martek Biosciences, Columbia, Md). Both oils are >95% triglycerides and essentially devoid of EPA.  The remainder of the fat was identical in the 3 formulas, with 40% medium-chain triglycerides, 21-22% LA and 3-3.1% LNA.  After hospital discharge term formula without DHA and ARA; no solid food. | Dose:  No added DHA or ARA  Formulation:  Formula was similar to Enfamil Premature Formula (Mead Johnson Nutritionals, Evansville, Ind) providing 81.3 kcal/100 ml with 3 g protein, 11.1 g carbohydrate, and 5.1 g fat/100 kcal  After hospital discharge term formula without DHA and ARA; no solid food. | Duration:  Median = 30 days  Started:  When enteral feed of 90 kcal/kg/d was reached; 17 days after birth  Ended:  At hospital discharge | Primary:  Non cognitive  Vision:  Visual acuity (Binocular preferential looking) by Teller Acuity Card procedure at 48 and 57 weeks (4 mo of post term) post-menstrual age | Visual acuity:  No significant difference between the 3 formula groups at 48 or 57 weeks PMA.  Visual acuity was significantly higher in the breast-fed term infants than in the premature infants at 57 weeks PMA, but not at 48 weeks PMA. |
| Uauy et al 1990  USA  RCT | Sample:  VLBW infants, BW 1000-1500 g, free from major neonatal morbidity, able to receive enteral feedings 70-120 kcal/kg by day 10 of life.    Age & Weight:  Mean values in breast milk group: 1308±117 g, 30.4±1.1 weeks;  In formula A group: 1340±106 g, 30.9±1.6 weeks;  In formula B group: 1224±92 g, 29.6±1.6 weeks; In formula C group: 1281±101, 30.7±1.2 weeks.  Randomized:  44 infants; 32 in 3 formula groups: 10 in formula A, 10 in formula B and 12 in formula C;  10 in breast milk group.  Followed up:  42/44 (95%) | Dose:  A) 24% LA and 0.5 % ALA of total fat;  B) 21% LA and 2.7% ALA of total fat;  C) 20% LA, 1.4 % ALA, 0.65% EPA, and 0.35% DHA of total fat.  Formulation:  All formulas contained 2.4g protein (whey:casein 60/40), 8.9 g carbohydrates and 4.1 g fat per 100 ml with varying amounts of EFA.  Formula A: low in all n-3 fatty acids  Formula B: contained ALA but no n-3 LCPUFA  Formula C: ALA and n-3 LCPUFA | Dose:  12.7% LA, 0.8% ALA, 0.5% ARA, 0.1% EPA, 0.29% DHA (breast milk)  Formulation:  Breast milk, infant’s mother’s milk fortified with human milk fortifier (Enfamil, Mead Johnson Bristol-Myers Company, Evansville, IN)  Mean intake of breast milk was >75%, and was supplemented with formula C if needed. | Duration:  20-35 days; approx. 6 weeks  Started:  Day 10 of life  Ended:  Day 45 of life; 36 weeks of post-conception or at discharge from hospital | Primary:  None defined (no power calculation)  Electrophysiology:  Retinal function (full-field ERG) at 36 weeks post-conception | ERG:  Rod b-wave thresholds were significantly higher in the n-3 deficient group (formula A) than in the groups receiving long-chain n-3 (human milk and formula C). Rod thresholds were identical in infants receiving EPA+DHA supplemented formula (formula C) and human milk-fed infants.  Maximum amplitudes (log Vmax) were lowest in infants given formula A, which provided predominantly n-6 FA, and highest in infants fed human milk or long-chain n-3-enriched formula.  Post-hoc comparisons of Vmax for group B receiving solely 18:3 n-3 versus the human milk-fed group gave a p value of<0.05, and for group B versus formula C gave a p value of <0.06. |
| Birch et al 1992  USA  RCT | Sample:  VLBW AGA infants, BW 1000-1500 g, free from major neonatal morbidity and able to receive enteral feedings 70-120 kcal/kg by day 10 of life.  Age & Weight:  Mean GA of the randomized infants 30.4±1.5 weeks  Randomized:  71 were randomized to three different test diets:  19 in formula A, 24 in formula B and 28 in formula C;  10 in breast milk group (non-randomized).  Followed up:  81/81 (100% at 36 weeks of post-conception);  52/81 (64%, at 57 weeks of post-conception) | Dose:  A) 24% LA and 0.5 % ALA of total fat;  B) 21% LA and 2.7% ALA of total fat;  C) 20% LA, 1.4 % ALA, 0.65% EPA, and 0.35% DHA of total fat.  Formulation:  Formula A: corn oil based, low in all n-3 FAs, corresponded to the fat composition of Enfamil Preterm (Mead-Johnson) in 1987.  Formula B: soy oil-based, ample 18:3 n-3 but no lc n-3 (DHA).  Formula C: supplemented with 18:3 n-3 and marine oils, comparable to human milk in DHA content (0.29% versus 0.35%).  All formulas prepared especially for this study and provided by Mead-Johnson Nutritional Group, Evansville, IL | Dose:  12.7% LA, 0.8% ALA, 0.5% ARA, 0.1% EPA, 0.29% DHA of total fat  Formulation:  Breast milk from the infant’s mother;  >75% of the diet as breast milk  Supplemental HM fortifier Enfamil, Mead-Johnson and soy/marine oil formula if the mothers were unable to provide all the needed breast milk. | Duration:  6 months  Started:  At 10 days of life  Ended:  At 57 weeks of post-conception | Primary:  None defined (no power calculation)  Electrophysiology:  Retinal function (ERG) at 36 and 56 weeks post-conception | ERG:  At 36 weeks of post-conception infants fed formula without n-3 and LCPUFA had significantly higher rod thresholds than infants receiving n-3 and LCPUFA supplemented formula or breast milk, but no significant differences at 57 weeks of post-conception.  No significant differences in cone function between different diet groups at 36 or 57 weeks of post-conception. |
| Birch EE et al 1992  USA  RCT | Sample:  Healthy VLBW AGA infants, BW 1000-1500g, GA 27-33 weeks post-conception  Age & Weight:  Mean values in corn oil group: 1342 g, 30.5 weeks;  Mean values in soy oil group:  1277 g, 30.1 weeks;  Mean values soy/marine oil group: 1305 g, 30.4 weeks;  Mean values breast milk group: 1265 g, 30.0 weeks.  Randomized:  83;  73 were randomized to three different formula groups;  10 in breast milk group (non-randomized).  Followed up:  50/83 (60%) at 57 weeks post-conception (Hoffman et al 1993)  No data at 36 weeks post-conception | Dose:  A) 24% LA and 0.5 % ALA of total fat;  B) 21% LA and 2.7% ALA of total fat;  C) 20% LA, 1.4 % ALA, 0.65% EPA, and 0.35% DHA of total fat.  Formulation:  1. Corn oil, based on medium-chain triglyceride (MCT), coconut oil, and corn oil and provided only LA (18:2 n-6) as EFA (corresponded to the 1987 formulation of Enfamil Premature, Mead Johnson, Evansville, IN)  2. Soy oil, based on MCT, coconut oil, and soy oil, provided LA (18:2 n-6) and ALA (18:3 n-3)  3. Soy/marine oil, an experimental product similar to the soy oil formula but supplemented with marine oils, provided DHA (0.4%) similar to that found in preterm human milk. | Dose:  12.7% LA, 0.8% ALA, 0.5% ARA, 0.1% EPA, 0.29% DHA  Formulation:  Breast milk;  >75% of the diet as breast milk  Supplemental HM fortifier Enfamil, Mead-Johnson and soy/marine oil formula if the mothers were unable to provide all the needed breast milk. | Duration:  26-27 weeks  Started:  At 10 days of life  Ended:  At 57 weeks of post-conception (equivalent to 4 months of post term) | Primary:  None defined (no power calculation)  Electrophysiology/ Vision:  VEP at 36 and 57 weeks post-conception;  Forced-choice preferential-looking (FPL) at 57 weeks post-conception | VEP and FPL:  Soy/marine oil group had significantly better VEP acuity at 36 and 57 weeks and significantly better FPL acuity at 57 weeks than infants in corn oil group.  At 36 weeks the soy oil group had significantly poorer VEP acuity than the human milk group.  At 57 weeks the soy oil group had significantly poorer VEP acuity than the soy/marine oil group. |
| Carlson et al 1993  USA  RCT | Sample:  VLBW AGA infants, BW 748-1398 g, no mechanical ventilation, intraventricular hemorrhage, retinopathy of prematurity or NEC, no maternal substance abuse; able to tolerate enteral intakes >110 kcal/kg/d for 5-7 days.  Age & Weight:  Mean values in the control group: 1074±193 g and 29±2;  Supplemented group:  1133±163 g, 29±2 weeks  Randomized:  79  Followed up:  67/79 (85%);  34 in the control group and 33 in the supplemented group | Dose:  0.3% EPA and 0.2% DHA (of total FAs) and >3% LNA  Formulation:  Commercially available preterm formula (Similac Special Care, Ross products Division) until discharge (1800g) and term formula (Similac with Iron, Ross products division) from discharge to 9 mo past term.  Both formulas contained >3% LNA (of total FAs) and were enriched with marine oil to contain 0.3% EPA and 0.2% DHA (of total FAs). | Dose:  No LCPUFA, >3% LNA  Formulation:  Commercially available preterm formula (Similac Special Care, Ross products Division) until discharge (1800 g) and term formula (Similac with Iron, Ross products division) from discharge to 9 mo past term.  Both formulas contained >3% LNA (of total FAs). | Duration:  Around 11 months  Started:  At around 3 weeks after birth (when infants tolerated >110 kcal/kg/d of formula)  Ended:  9 months past term | Primary:  Unclear reporting  Vision:  Visual acuity (Teller Acuity Card procedure) at 2, 4, 6.5, 9 and 12 months CA | Visual Acuity:  At 2 and 4 months resolution acuity was significantly better in the marine oil supplemented group compared to the control group.  The positive effect of marine oil supplementation was not seen between 6.5 and 12 months. |
| Werkman & Carlson 1996  USA  RCT  (same study population as in Carlson et al 1993) | Sample:  VLBW AGA infants, BW 748-1398 g, no mechanical ventilation, intraventricular hemorrhage, retinopathy of prematurity or NEC, no maternal substance abuse; able to tolerate enteral intakes >110 kcal/kg/d for 5-7 days.  Age & Weight:  Mean values in the control group: 1074±193 g, 29±2;  In the supplemented group: 1133±163 g, 29±2 weeks  Randomized:  67  Followed up:  64/67 (96%) | Dose:  0.2% DHA (of total FAs) and >3% ALA  Formulation:  Commercially available preterm formula (Similac Special Care, Ross products Division) until discharge (1800g) and term formula (Similac with Iron, Ross products division) from discharge to 9 mo past term.  Both formulas contained >3% ALA (of total FAs) and were enriched with marine oil to contain 0.3% EPA and 0.2% DHA (of total FAs). | Dose:  No LCPUFA, >3% LNA  Formulation:  Commercially available preterm formula (Similac Special Care, Ross products Division) until discharge (1800 g) and term formula (Similac with Iron, Ross products division) from discharge to 9 mo past term.  Both formulas contained >3% ALA (of total FAs). | Duration:  Around 11 months  Started:  At mean postnatal age of 25 days  Ended:  9 months past term | Primary:  None defined (no power calculation)  Vision:  Novelty preference (Visual recognition memory);  Visual attention (Fagan Test of Infant Intelligence) at 29 weeks, 39 weeks and 52 weeks CA | Novelty preference and Visual attention:  In paired comparisons of novel and familiar stimuli, DHA-supplemented and control infants had the same novelty preference, but supplemented infants had more discrete looks to both novel (P< 0.03) and familiar (P< 0.02) stimuli and a shorter overall look duration (P< 0.03) at all study ages. |
| Carlson et al 1996  USA  RCT | Sample:  VLBW AGA infants, BW 747-1275 g, no intraventricular or periventricular hemorrhage, congenital anomalies,no maternal substance abuse; infants who required long periods of supplemental oxygen and developed bronchopulmonary dysplasia (BPD) were included; infants remained in the study if achieved full enteral feeding of 100 kcal/kg/d by 6 wk of age  Age & Weight:  Mean values in the control group: 1112±106 g, 28.6±1.3 wk (no BPD); 975±151 g, 27.5±1.6 wk (BPD);  In the DHA supplemented group: 1069±153 g, 28.5±1.2 wk (No BPD); 947±130 g and 27.0±1.1 wk (BPD)  Randomized:  94  Followed up:  59/94 (63%) completed the study through at least 2 months past term;  51/94 (54%) at 12 mo assessment | Dose:  0.2% DHA, 0.06% EPA and 2.5% ALA (of total FAs)  Formulation:  Standard preterm formula (Similac Special Care, Ross Product Division, Columbus, OH) enriched with marine oil  After 2 mo of CA the infants in both groups were provided a standard, un-supplemented formula (Similac With Iron, Ross Laboratories) | Dose:  2.5% ALA (of total FAs)  Formulation:  Standard preterm formula (Similac Special Care, Ross Product Division, Columbus, OH)  The basic oil blend of preterm formula consisted of 50% medium-chain triacylglycerol, 30% soybean oil and 20% coconut oil. | Duration:  5 months  Started:  At 3-5 day of life  Ended:  2 months of CA (2 months after expected term) | Primary:  Non cognitive  Vision:  Teller Acuity Card procedure at 0, 2, 4, 6, 9 and 12 months CA | Visual acuity:  Marine oil supplemented infants without BPD had significantly improved acuity at 2 mo; whereas infants with BPD had poorer acuity at 2 and 4 mo than the infants fed control formula.  At 6, 9 and 12 mo neither experimental diet nor BPD were related to visual acuity. |
| Carlson & Werkman 1996  USA  RCT  (same study population as in Carlson et al 1996) | Sample:  VLBW AGA infants,BW 747-1275 g, no intraventricular or periventricular hemorrhage, congenital anomalies, no maternal substance abuse; infants who required long periods of supplemental oxygen and developed bronchopulmonary dysplasia (BPD) were included;  Age & Weight:  Mean values in the control group: 1050±149 g and 28.2±1.5 wk;  In the DHA supplemented group: 1027±153 g and 27.9±1.5 wk.  Randomized:  59  Followed up:  27/59 (46%); 12 in the control group (5 with BPD), 15 in the DHA supplemented group (6 with BPD) | Dose:  0.2% DHA, 0.06% EPA and 3% LNA (of total FAs)  Formulation:  Preterm formula (Similac Special Care, Ross Product Division, Abbot Laboratories, Columbus, OH) enriched with marine oil with high ratio of DHA to EPA  From 2 mo to 12 mo of CA the infants in both groups were provided a commercially available formula (Similac With Iron) LNA as an only source of n-3 FAs | Dose:  3% LNA (of total FAs)  Formulation:  Preterm formula (Similac Special Care, Ross Product Division, Abbot Laboratories, Columbus, OH) | Duration:  5 months  Started:  At 2-5 days of life  Ended:  2 months past term | Primary:  Non-cognitive  Vision:  Novelty preference (Visual recognition memory);  Visual attention (Fagan Test of Infant Intelligence) at 12 months CA | Novelty preference and Visual attention:  The DHA-supplemented group compared with the control group had significantly more and shorter duration looks in comparisons of familiar and novel stimuli.  Infants with BPD differed from those without BPD in that they had significantly fewer total looks during familiarization with the Fagan Test. |
| Faldella et al 1996  Italy  RCT | Sample:  Preterm infants, GA<33 weeks, AGA, no malformations, visual, neurological, acoustic, gastro-intestinal illnesses, or perinatal asphyxia; normal fundus oculi.  Age & Weight:  Mean values in breast milk group: 1590±406 g and 31.8±1.7 weeks;  For LCPUFA-enriched preterm formula group:  1583±310 g and 31.1±1.2 weeks;  For preterm formula group: 1463±273 g and 31.3±1.2 weeks  Randomized:  49; 23 in LCPUFA-enriched group, 26 in preterm formula group;  17 in breast milk group (non-randomized)  Followed up:  21/23 (91%) in LCPUFA-enriched group; 25/26 (96%) in preterm formula group; 12/17 (71%) in breast milk group | Dose:  1. DHA concentration 0.23% of total lipids (0.08% EPA; 0.35% ARA, 0.40%LNA, 12.2% LA)  2. No detectable DHA  (0.04% EPA; 0.01% ARA, 0.25% LNA, 18.6% LA)  Formulation:  1. Preterm formula enriched with LCPUFA (Preaptamil with Milupan, Milupa AG, Friedrichsdorf, Germany)  2. Traditional preterm formula  Formula groups received less than 25% of their caloric intake from breast milk. | Dose:  DHA concentration 1.22% of total lipids;  (1.23% ARA, 0.66% LNA, 12.1% LA)  Formulation:  Mother’s breast milk, at least 75% of the intake + LCPUFA enriched preterm formula | Duration:  Around 5 months  Started:  10 day of life  Ended:  At 52 weeks post-conceptional age | Primary:  None defined (no power calculation)  Electrophysiology:  Flash VEP, ERG and Flash BAEP at 52 weeks post-conceptional age | VEP:  Significant differences in the morphological patterns of responses of the three diet groups.  The latencies of late component waves N4 and P4 were significantly longer in the traditional preterm formula group than in preterm formula enriched with LCPUFA or breast milk group (which showed similar N4 and P4 latencies).  ERG and BAEP:  No significant differences between the three groups in recordings. |
| Fang et al 2005^1^  Taiwan  RCT | Sample:  Healthy, AGA, preterm infants, GA 30-37 weeks, no human milk given  Age & Weight:  Mean values in the LCPUFA group: 1980±110 g and 33.3±0.5 weeks;  Mean values in the control group: 1990±120 g and 33.0±0.5 weeks  Randomized:  28; 16 in the LCPUFA group and 11 in the control group (1 infant was excluded after randomization)  Followed up:  BSID-II: at 6 mo 25/27 (93%) and at 12 mo 22/27 (81%);  VEP: at 4 mo 24/27 (89%) and at 6 mo 23/27 (85%);  Lea grating acuity cards, Hiding Heidi ‘FACE’ cards: 27/27 (100%) | Dose:  Formula providing 0.05% DHA and 0.10% ARA  Formulation:  Supplemented formula, Neoangelac Plus by Multipower Enterprise providing 0.05% of DHA and 0.10% ARA.  Infants given >110 kcal/kg/d during the first 4 months and >70 kcal/kg/d from 4 to 6 months. | Dose:  No added DHA and ARA  Formulation:  Unsupplemented formula, Neoangelac by Multipower Enterprise providing an adequate ratio of linoleic acid:α-linolenic acid (10:1).  Infants given >110 kcal/kg/d during the first 4 months and >70 kcal/kg/d from 4 to 6 months. | Duration:  6 months  Started:  Approximately 2 weeks after birth;  Mean GA at study entry in the LCPUFA group 35.6±0.2 weeks and in the control group 35.5±0.2  Ended:  6 months later | Primary:  None defined (no power calculation)  Cognition/ Behavior:  BSID-II (MDI, PDI) at 6 and 12 months after study entry;  Electrophysiology/ Vision:  VEP (steady-state visual evoked potential) at 4 and 6 months after study entry;  Lea grating acuity cards and Hiding Heidi ‘FACE’ cards at 4 and 6 months after study entry | BSID-II  At 6 mo and 12 mo MDI and PDI scores were significantly higher (by repeated measures of ANOVA) in the LCPUFA group than in the control group.  VEP, Lea grating acuity cards,  Hiding Heidi ‘FACE’ cards  No significant differences between feeding groups at 4 mo or at 6 mo. |
| Van Wezel- Meijler et al 2002^1^  Netherlands  RCT | Sample:  Preterm infants in NICU, GA<34 wk, BW<1750 g, with normal neurological examination, no significant cerebral damage, retinopathy, chronic disease or feeding problems; mothers not breast feeding  Age & Weight:  Mean values in LCPUFA group: 1282±316 g and 30.4±1.5 wk;  Mean values in the control group: 1306±257 g and 30.4±1.6wk  Randomized:  42; 22 in the LCPUFA supplemented group and 20 in the un-supplemented group  Followed up:  42/42 (100%);  (not all outcome measures were available from all randomized infants although all completed the intervention period) | Dose:  0.34% DHA and 0.68% ARA of total fat.  Formulation:  Parenteral nutrition with negligible amounts of LCPUFA before starting enteral feeding with preterm formula.  Preterm formula (Nutricia, Zoetermeer, NL) supplemented with LCPUFA (DHASCO oil, produced by microalgae and ARASCO oil, produced by fungi; Martek Inc., Columbia, USA) until a weight of 3000 g.  Thereafter term infant formula supplemented with LCPUFA | Dose:  No added DHA or ARA  Formulation:  Parenteral nutrition with negligible amounts of LCPUFA before starting enteral feeding with preterm formula.  Standard un-supplemented preterm formula (Nutricia, Zoetermeer, The Netherlands) until a weight of 3000 g.  Thereafter standard un-supplemented term infant formula | Duration:  Around 8-9 months  Started:  3-7 days from birth  (full enteral feeding was achieved between 28 and 35 days of life)  Ended:  6 months of CA | Primary:  None defined (no power calculation)  Cognition/ Behavior:  BSID (MDI, PDI) at 3, 6, 12 and 24 mo CA  Electrophysiology/ Vision:  Visual acuity by Teller card procedure at 3, 6, 12 and 24 mo CA;  Flash-VEP at 3 and 12 mo CA  Neuroimaging:  MRI (global and cerebral visual system myelination) | BSID:  There was a tendency towards better scores for MDI and PDI in the control group. Based on repeated measurement analysis the differences were not significant for MDI, whereas there was a systematic significant difference between the two groups with respect to PDI (P=0.04) in favor of the control group, which disappeared after adjustment for BW and number of small-for-gestational-age infants (P=0.8).  Flash-VEP:  No significant differences at either test age (3 or 12 mo) between the DHA and control groups.  Visual acuity:  At all test ages mean acuity values were slightly higher, but not statistically significant in the DHA than in the control group. Group differences did not change between 3 and 24 mo of CA, so visual acuity did not increase more rapidly in the DHA group.  MRI:  No significant differences in global or visual myelination at 3 mo or 12 mo or in the progress of myelination from 3 mo to 12 mo between DHA and control groups. |
| Smithers et al 2008  Australia  RCT  (a pilot study to Makrides et al 2009) | Sample:  Infants with GA<33 weeks, free from major congenital or chromosomal abnormalities; infants with medical comorbidities common to preterm infants were included.  Age & Weight:  Mean values in high DHA group: 1312±439 g, 29.1±2.6 weeks;  Mean values in control group: 1358±433 g, 29.5±2.2 weeks  Randomized:  143; 74 in the high DHA group and 69 in the control group  Followed up:  At 2 mo: 64/74 (86%) in the high DHA group and 61/69 (88%) in the control group;  At 4 mo: 64/74 (86%) in the high DHA group and 63/69 (91%) in the control group | Dose:  1% DHA  Formulation:  A combination of breast milk and formula  Breast milk: Lactating mothers consumed six 500-mg DHA-rich tuna oil capsules/d to achieve a breast milk DHA concentration approximately 1% of total fatty acids without altering the naturally occurring concentration of ARA in breast milk.  or  Preterm formula: containing approximately 1.0% DHA and 0.6% ARA. | Dose:  0.3% DHA  Formulation:  A combination of breast milk and formula  Breast milk: Lactating mothers consumed six 500-mg soy oil capsules that did not change the fat content or fatty acid composition of their milk.  or  Standard preterm formula: containing approximately 0.3% DHA and 0.6% ARA. | Duration:  Approx. 10 weeks  Started:  After birth and within 5 days of receiving any enteral feeds  Ended:  At term expected delivery date | Primary (visual acuity):  Sweep VEP at 4 months CA  Electrophysiology:  VEP acuity and latency at 2 and 4 months CA | VEP acuity:  No difference in acuity between the high DHA and control group at 2 months.  At 4 months, acuity was significantly higher in the high DHA group than in the control group.  VEP latency:  Latencies were not significantly different between high DHA and control groups at 2 months or 4 months. |
| O’Connor et al. 2001^1^  USA, UK, Chile  RCT | Sample:  Preterm infants, GA<33 wk, BW 750 -1805 g, free from serious congenital abnormalities, periventricular or intraventricular hemorrhage and no major surgery or maternal incapacity, liquid ventilation, asphyxia resulting in severe and permanent neurologic damage, or uncontrolled systemic infection at the time of enrolment.  Age & Weight:  Mean values control group: 1287±272 g, 29.6±1.9 wk;  Mean values fish/fungal oil group: 1305±293 g, 29.8±2.1 wk;  Mean values egg-TG/fish group: 1309±286 g and 29.7±2.0 wk  Randomized:  470  Followed up:  376/470 (80%) at 12 mo CA | Dose:  From birth to term:  1. 0.43% ARA + 0.27%DHA and 0.08% EPA (fish/fungal oil)  2. 0.41% ARA + 0.24% DHA and 0% EPA (egg-TG/fish oil)  From term to 12 mo of CA:  1. 0.43% ARA + 0.16% DHA and 0% EPA (fish/fungal oil)  2. 0.41% ARA + 0.15% DHA and 0% EPA (egg-TG/fish oil)  Formulation:  Human milk and/or the assigned in-hospital pre-term formula (modified version of Similac Special Care ready-to-feed [24 kcal/fl oz]; SSC) with AA- and DHA-enriched oils until term CA.  From term CA, infants received post-discharge nutrient-enriched formula (modified version of NeoSure powder [22 kcal/fl oz]) with the same sources of AA+DHA and/or human milk. | Dose:  No supplemental ARA or DHA  Formulation:  Human milk and/or the assigned in-hospital pre-term formula (modified version of Similac Special Care ready-to-feed [24 kcal/fl oz]; SSC) without ARA- and DHA-enriched oils until term CA.  From term CA, infants received post-discharge nutrient-enriched formula (modified version of NeoSure powder [22 kcal/fl oz]) without the sources of ARA+DHA and/or human milk. | Duration:  Around 14 months  Started:  Within 72 hours of the first enteral feeding (infants could be enrolled as long as enteral feeding was initiated by the 28th day of life).  Ended:  12 months of CA | Primary:  BSID (MDI and PDI) at 12 months of CA  Cognition/ Behavior:  The Fagan Test of Infant Intelligence at 6 and 9 mo CA;  MCDI vocabulary checklist at (9 and 14 mo CA  Electrophysiology/ Vision:  VEP at 4 and 6 mo CA;  Teller Acuity Card Procedure at 2, 4, and 6 mo CA | Teller Acuity Card Procedure:  No significant group difference at 4, 6 mo CA.  VEP:  No significant group difference at 4 mo CA. At 6 mo CA, the mean VEP acuity was significantly greater in both intervention (ARA + DHA) groups than in the control group. The mean VEP acuity of infants in the ARA + DHA-supplemented group increased between 4 and 6 mo CA, but the mean VEP acuity of those in the control group did not.  BSID:  No significant differences between groups for MDI. A statistically significant feeding by BW stratum interaction was found for PDI (P=0.005) in infants who consumed >80% of their feeding as study formula and/or human milk.    Fagan Test of Infant Intelligence:  The mean novelty preference was significantly greater in the ARA+DHA (egg-TG/fish)-group than in the control and ARA+DHA (fish/fungal)-group at 6 mo CA.  MCDI:  No significant group difference at 9, 14 mo CA. |
| Leaf et al 1996  Australia  Observational study | Sample:  Infants born with GA <32 weeks; free from congenital anomaly and severe intra-/periventricular hemorrhage  Age and Weight:  Mean values in the high breast milk group: 1157±301 g, 28.5±2.4 weeks;  Mean values in the low breast milk group: 1211±340 g, 28.4±2.5 weeks  Non-randomized study:  18 infants; 9 in the high breast milk group, 9 in low breast milk group  Followed up:  Satisfactory ERG recordings obtained in 16/18 (89%) infants; satisfactory visual acuity measurements in 15/18 (83%) infants | Dose:  ARA 32mg/kg/day and DHA 17mg/kg/d when 150 ml/kg/d was consumed  Formulation:  In high breast milk group (HBM): mainly breast milk, fortified with  Breast Milk Fortifier (Mead Johnson). BMF contained protein, carbohydrate and minerals but no lipids.    TPN was provided to those with BW<1500 g and who were considered unlikely to tolerate enteral feeds within the first few days of life: A solution of glucose and amino acids (Vamin-N, Pharmacia Ltd.) and Intralipid 20% (Pharmacia Ltd.) as a source of lipids.  Mean breast milk intake 74%, formula 9.6% and TPN 17% during the hospital stay. | Dose:  No ARA and DHA (although on maximum Intralipid intake of 15 ml/kg/d infants would have received 6.4 mg/kg/d of ARA and 5.8 mg/kg/d of DHA)  Formulation:  In low breast milk group (LBM): mainly infant formula, 'Enfalac' formula (Mead Johnson), 'Premature Enfalac' until a weight of 2000 g and then standard 'Enfalac'; lipids as a mixture of vegetable oils, no LCPUFA.  Mean breast milk intake 17.5%, formula 57.5% and TPN 25.1% during the hospital stay. | Duration:  Approx. 12 weeks  Started:  Within the first four days of life  Ended:  At discharge or 40 weeks of PCA | Primary:  None defined (no power calculation)  Visual Acuity:  Visual acuity by the Teller Acuity Card procedure of forced choice of preferential looking at 40 weeks and 3 months  Electrophysiology:  Visual (retinal) function by ERG (at 40 weeks);  Cone (photopic, i.e. light-adapted) and rod (scotopic, i.e. dark-adapted) responses to light flashes;    Enhanced retinal maturation results in shortening of implicit time of response and increase in amplitude of ERG recordings | ERG:  The scotopic b-wave implicit time was significantly longer in infants in the HBM group (P=0.01). No significant differences between the groups in other ERG responses.  Visual Acuity:  No significant difference between the two diet groups at 40 weeks PCA or 3 months of CA. |

^1^ Publications duplicated in the table because reporting both cognitive and visual outcomes

TABLE S6. MICRONUTRIENT & SPECIFIC INGREDIENT STUDIES

| Author, year, country, design | Study population | Intervention diet  (dose and formulation) | Control diet  (dose and formulation) | Intervention/ Observation period | Outcome measures | Effect of the intervention/  Findings of cohort study |
| --- | --- | --- | --- | --- | --- | --- |
| VITAMIN A | | | | | | |
| Ambalavanan et al 2005  USA  RCT | Sample:  ELBW infants receiving respiratory support 24 hours after birth  Age & Weight:  Mean values in Vit A group 769±135 g and 26.7±1.9 weeks;  Mean values in control group 766±139 g and 26.7±1.7 weeks  Randomized:  807; 405 in Vit A and 402 in the control group  Followed up:  579 of the 658 surviving infants (88%) | Dose:  Vit A 5000 IU 3 times per week  Formulation:  Intramuscular injections | Dose:  Sham injections  Formulation:  Intramuscular injections | Duration:  4 weeks  Started:  Immediately after birth  Ended:  A month after birth | Primary:  None defined (no power calculation)  Cognition/ Behavior:  Neurodevelopmental impairment  BSID-II (MDI, PDI) at 18 to 22 months CA | MDI, PDI and NDI  No significant differences between Vit A supplemented and control group in any of the outcome measures |
| IRON | | | | | | |
| Berglund et al 2011  Sweden  RCT | Sample:  Marginally LBW infants, BW 2000-2500 g, SGA term infants and preterm infants with GA<37 weeks; no diseases, no blood transfusions or iron supplementation  Age & Weight:  Mean values 2 mg iron/kg/d group: 2310±130 g, 36.6±2.1 weeks;  Mean values 1 mg iron/kg/d group: 2270±150 g, 36.5±1.8 weeks;  Mean values placebo group: 2290±140 g, 36.5±1.8 weeks  Randomized:  285 infants  Followed up:  218/285 (76%) infants had ABR measured | Dose:  1 mg iron/kg/d  and  2 mg iron/kg/d  Formulation:  Supplemental ferrous succinate drops;  Ferrous succinate mixture (Ferromyn S; Astra Zeneca, Södertälje, Sweden) containing 3.7 mg/ml of iron; | Dose:  0 mg iron/kg/d (placebo)  Formulation:  The placebo mixture by Apoteket Production & Laboratories, Stockholm, and had similar taste and color than iron mixture. | Duration:  4.5 months  Started:  6 weeks of life  Ended:  6 months of life | Primary (Electrophysiology):  CCT at 6 months postnatal age  Electrophysiology:  ABR absolute wave V latency at 6 months postnatal age | CCT  Significantly higher in 2 mg feeding group than in placebo group.  No significant correlation between CCT and iron intake or status; therefore the difference in CCT may not be due to iron.  ABR wave V latencies:  No significant group differences.  No significantly prolonged ABR latencies in iron deficient infants. |
| Berglund et al 2013  Sweden  RCT | Sample:  Marginally LBW (2000– 2500 g) infants, free from chronic diseases, previous blood transfusion or iron supplementation, anemia or other hematologic disorder.  Age & Weight:  Mean values in high iron group: 2300±140 g, 36.4±2.1 weeks;  Mean values in low iron group: 2270±150 g, 36.5±1.8 weeks;  Mean values in control group: 2280±150 g, 36.5±1.7 weeks  Randomized:  285 infants (3 groups, 95 each);  Followed up:  224 (79%);  in addition, 95 healthy full term and no low birth weight infants as control subjects | Dose:  1 mg iron/kg/day, and  2 mg iron/kg/day  Formulation:  Supplemental ferrous succinate drops;  Ferrous succinate mixture (Ferromyn S; Astra Zeneca, Södertälje, Sweden) containing 3.7 mg/ml of iron;  No dietary recommendations apart from general Swedish infant dietary recommendations, i.e. exclusive breast-feeding until age 4 to 6 months. | Dose:  0 mg/kg/day;  no supplemental iron  Formulation:  Supplement placebo drops;  The placebo mixture by Apoteket Production & Laboratories, Stockholm, and had similar taste and color than iron mixture.  No dietary recommendations apart from general Swedish infant dietary recommendations, i.e. exclusive breast-feeding until age 4 to 6 months. | Duration:  4.5 months  Started:  6 weeks of age  Ended:  6 months of life | Primary:  WPPSI-III at 3.5 yrs  Cognition/ Behavior:  CBCL at 3.5 yrs | WPPSI-III  No significant differences between placebo and intervention groups or the healthy controls.  CBCL  The relative risk of having a score above the subclinical cut-off was 4.53 (1.44–14.21; P=.011) for placebo-treated versus iron-supplemented children.  There was a signiﬁcant difference between placebo and the iron-supplemented groups in the CBCL subscales “emotionally reactive” (P=.040) and “attention problems” (P=0.022) and a similar trend in all subscales except “withdrawn.” |
| Steinmacher et al, 2007  Germany  RCT | Sample:  VLBW infants with BW <1301 g in level 3 NICU, free from major anomalies, hemolytic disease, twin-to-twin transfusion syndrome.  Age & Weight:  Mean values in the early iron group: 884±222 g, 27.6±2.2 weeks;  Mean values in the late iron group: 863±215 g, 27.4±2.5 weeks  Randomized:  204; 105 early and 95 late group;  Followed up: 164 of the surviving 194 infants (85%). | Dose:  Early group: 2 mg iron/kg/d as soon as 100 ml/kg/d of enteral feedings were reached.  Formulation:  Ferrous sulfate as enteral supplementation with the milk feeds (either breast or formula). If iron deﬁciency was diagnosed, iron was started at 4 mg/kg per day. | Dose:  Late group: 2 mg iron /kg/d at the age of 61 days of life.  Formulation:  Ferrous sulfate as enteral supplementation with the milk feeds (either breast or formula). If iron deﬁciency was diagnosed, iron was started at 4 mg/kg per day. | Duration:  Not reported  Started:  In early iron group at a median age of 14 days (range: 7– 61 days)  In late iron group at a median age of 61 days (range: 12–74 days)  Ended:  Not reported | Primary:  Iron status and iron deficiency  Cognition/ Behavior:  GMFCS,  LOS-KF 18,  KABC (MPC and achievement scale),  Visual assessment,  CBCL  at median of CA of 5.3 years | GMFCS:  More neurologic abnormalities in the late-iron (25%) than in the early-iron group (12%, P<0.04).  KABC  Suggestive trends toward improved cognitive development with early iron group in the overall MPC, the subscale of simultaneous processing, and the achievement scale.  LOS-KF 18 and CBCL  No difference in the proportion of children with impaired motor coordination or abnormal behavior between early- and late-iron groups.  Impaired visual perception  In children with normal MPC: no difference in the incidence of impaired visual perception between the groups. |
| PROBIOTICS | | | | | | |
| Chou et al 2010  Taiwan  RCT | Sample:  Preterm VLBW infants who began enteral feeding and lived more than 7 days after birth  Age & Weight:  Mean values in probiotics group: 1103±232 g, 28.5±2.3 weeks;  Mean values in control group: 1097±231 g, 28.5±2.3 weeks  Randomized:  367 infants enrolled; 27 died in hospital and 5 after hospital discharge  Followed up:  301/335 (90%) were evaluated at 3yrs; 153 in the probiotics group, 148 in the control group | Dose:  Breast milk with probiotics  Formulation:  Inﬂoran, 125 mg/kg per dose (containing *Lactobacillus acidophilus* 10^9^ colony-forming units [from the American Type Culture Collection in 1973] and *Biﬁdobacteria infantis* 10^9^ colony-forming units [from the American Type Culture Collection in 1973; Swiss Serum and Vaccine Institute, Berne, Switzerland]), twice daily with breast milk. | Dose:  Breast milk without probiotics  Formulation:  Breast milk | Duration:  Approx. 40 days  Started:  1 week after birth  Ended:  At discharge from hospital,  Mean length in the study for probiotics group 46.3±25.9 days and mean length for control group 47.4±24.1 days | Primary:  Death or Neurodevelopmental Impairment (NDI =the presence of one or more of the following: BSID-II MDI < 70, PDI < 70, bilateral blindness, hearing impairment requiring ampliﬁcation (> 55 dB in both ears), or moderate to severe CP (requiring ambulatory assistance))  Cognition/ Behavior:  BSID-II (MDI and PDI) at 3 years CA | NDI  No statistically significant group difference in any of the criteria of NDI.  MDI and PDI  No statistically significant difference between the probiotics and the control group. |
| Romeo et al 2011  Italy  RCT | Sample:  Preterm infants, GA<37 weeks, BW<2500 g, on stable enteral feeding within 72h of birth  Age and Weight:  Mean values in the *L. reuteri* group: 1998.7±439 g, 33.8±1.8 weeks; Mean values in the  *L. rhamnosus* group: 1940.7±590 g, 33.3±1.6 weeks;  Mean values in the control group: 1945.7±465 g, 33.3±2.1 weeks.  Randomized:  249; 83 in the *L. reuteri* group, 83 in the *L. rhamnosus* group and 83 in the control group  Followed up:  249/249 (100%) infants had the HINE at 12 months of CA | Dose:  *L. reuteri* ATCC 55730: 10^8^ cfu (5 drops) once a day  *L. rhamnosus* ATCC 53103: 6x10^9^ cfu (1 capsule) once a day  Formulations:  *L. reuteri* ATCC 55730 drops in an oil formulation  *L. rhamnosus* ATCC 53103 powder in capsules | Dose:  No probiotics  Formulation:  none | Duration:  Not reported  Started:  Within 72 hours after hospitalization (1.42±0.49 days in *L. reuteri* group and 1.40±0.49 in *L. rhamnosus* group)  Ended:  After 6 weeks or at hospital discharge | Primary:  Incidence of enteric fungal colonization  Neurological assessment:  HINE at 12 months of CA  (The scores equal to or above 73 were regarded as optimal, and those below 73 as suboptimal) | HINE  No differences in the incidence of suboptimal scores in the groups treated with probiotics (n=10 with *L. reuteri*, n =13 with *L. rhamnosus*; P>0.05).  However, a significant higher incidence of suboptimal scores (P<0.05) was found for the control group (n=24) compared to both probiotic groups. |
| Sari et al 2012  Turkey  RCT | Sample:  Preterm infants, GA<33 weeks, BW<1500g, enteral feeding  Age and Weight:  Mean values in the probiotic group: 1241±264g,29.7±2.5 weeks;  Mean values in the control group: 1278±273g, 29.8±2.3 weeks  Randomized:  242; 121 in the probiotics group, 121 in the control group;  110 received probiotics, 111 control diet  Followed up:  86/110 (78%) with BSID in the probiotics group, 88/111 (79%) in the control group | Dose:  350.000.000 colony-forming units of probiotic, once a day  Formulation:  *Lactobacillus sporogenes* (DMG ITALIA SRL, Rome, Italy) | Dose:  No probiotics | Duration:  Mean = 35.6±23.7 days (followed up cohort); Mean = 36±22.5 days (probiotics group); Mean = 35.2±24.9 days (control group)  Started:  With the first feed (at the age of 1-2 days)  Ended:  Hospital discharge | Primary:  Neurodevelopmental Impairment  Cognition/ Behavior:  BSID-II (MDI, PDI) at 18 to 22 months CA | MDI and PDI:  No significant difference between the treatment groups.  NDI:  No significant difference between the treatment groups. |
| PREBIOTICS | | | | | | |
| LeCouffe et al 2014  The Netherlands  RCT | Sample:  VLBW infants, BW<1500 g and/or less than 32 weeks GA, free from congenital anomalies and diseases  Age & Weight:  Mean values in the prebiotic group: 1.37±0.4kg, 30.2±1.6 weeks;  Mean values in the control group: 1.26±0.3kg, 29.5±2.0 weeks  Randomized:  114; 55 in the prebiotic, 59 in the placebo group  Followed up:  93 (82)% | Dose:  Maximal 1.5 g/kg/day prebiotic  Formulation:  80% neutral (short-chain galacto-oligosaccharides and long-chain fructo-oligosaccharides) and 20% acidic (peptin-derived acid oligosaccharides). Prebiotic powder added to breast milk or  preterm formula, according to parent choice  Exclusive breast feeding in 56% infants | Dose:  Maximal 1.5 g/kg/day placebo  Formulation:  Maltodextrin powder added to breast milk or preterm formula, according to parent choice  Exclusive breast feeding in 44% infants | Duration:  28 days  Started:  3 days after birth  Ended:  30 days after birth | Primary:  Infectious morbidity  Neuromotor Function:  Neurological assessment according to Touwen et al. (1976) at 0, 3, 6, and 12 months CA and Alberta Infant Motor Scale (AIMS) at 6 and 12 months CA | No significant group differences in any outcome. |
| SPHINGOMYELIN | | | | | | |
| Tanaka et al 2013  Japan  RCT | Sample:  VLBW infants, BW<1500 g, free from congenital anomalies and diseases  Age & Weight:  Mean values in the SM group: 1116±254 g, 29.1±2.1 weeks;  Mean values in the control group: 1100±353 g, 30.1±2.3 weeks  Randomized:  24; 12 in the intervention, 12 in the control group  Followed up:  100% | Dose:  20% sphingomyelin of all phospholipids in formula milk  Formulation:  Infant formula enriched with milk phospholipids.  The content of DHA and ARA in formula similar to the control formula.  During the feeding breast milk was given priority, and shortages were covered using test formulas.  During the first 4 weeks of life around 75% of infants were breast-fed; by the age of 8 weeks around 50% of infants were breast-fed.  Breast feeding = >80% of all milk from breast | Dose:  13% sphingomyelin of all phospholipids in formula milk  Formulation:  Infant formula enriched with egg yolk lecithin phospholipids.  The content of DHA and ARA in formula similar to the intervention formula.  During the feeding breast milk was given priority, and shortages were covered using test formulas.  During the first 4 weeks of life around 75% of infants were breast-fed; by the age of 8 weeks around 50% of infants were breast-fed. | Started:  Within 24 hours of birth | Primary:  None defined (no power calculation)  Cognition/ Behavior:  BSID-II (MDI, PDI and BRS) at 6, 12, 18 months CA;  Fagan test at 3, 6, 9 and 12 months CA;  Free play sustained attention test of Colombo at 18 months CA;  Memory for location test at 18 months CA  Electrophysiology:  VEP at 3, 6, 9 and 12 months CA | VEP:  No significant difference between the groups at any time point (3, 6, 9, 12 months).  Fagan test:  Mean scores were significantly higher in the SM group than in the control group at 12 months.  BSID-II (MDI and PDI):  No significant difference between the groups at any time point (6, 12 and 18 months).  BSID-II (BRS):  The orientation and emotional scores at 6, 12, and 18 months and the motor quality and total scores at 12 and 18 months were significantly higher in the SM than in the control group.  Sustained attention test:  Scores were significantly higher in the SM group compared to the control group (18 months).  Memory location test:  No significant group differences at 18 months. |
